# Supplementary material for: A systematic review and meta-analysis of the global prevalence and determinants of COVID-19 vaccine acceptance and uptake in people living with HIV
Source: Nat Hum Behav. 2023 Oct 30;8(1):100–14. doi: 10.1038/s41562-023-01733-3 (PMC10810755; doi:10.1038/s41562-023-01733-3)
Supplement: Supplementary file 1 — Supplementary Sections A–H. [file 41562_2023_1733_MOESM1_ESM.pdf]

nature human behaviour

Article

https://doi.org/10.1038/s41562-023-01733-3

A systematic review and meta-analysis of the global prevalence and determinants of COVID-19 vaccine acceptance and uptake in people living with HIV

In the format provided by the authors and unedited

SECTION A

Supplementary Table 1: Summary of the included studies

**Author & publication year**	**Country**	**Study design**	**Sampling method**	**Study setting**	**Study period**	**Sample size [T; M (%)]**	**Age: mean (SD) or median [IQR]**	**Intention A (%); U (%)**	**Factors associated with vaccine acceptance, hesitancy, and uptake**
Su et al[57]/2022	China	CS	SRS (PS)	Five cities (Chongzuo, Guigang, Laibin, Qinzhou, Yulin) in Guangxi	May 7 to June 1, 2021	903; 578 (64.0)	NA	A=658 (72.9); U=NA	Ethnicity (Zhuang versus Han), the higher level of education and believing that COVID-19 vaccination does not affect ART efficacy were associated with higher acceptance. The perception that COVID-19 vaccination is unsafe and less effective among PLHIV are associated with lower acceptance.
Buckley et al[58]/2022	US	CS	CoS (NPS)	Urban primary care clinic, New York City	March to May, 2021	223; NA	NA	A=167 (74.89); U=79 (35.43)	HIV control (undetectable VL less than 200), and age 35 to 65, and greater than 65 years old versus less than 35 years old were significantly associated with greater vaccine acceptance. Identifying as Black (vs White) is associated with lower odds of acceptance.
Zheng et al[59]/2021	China	CS	CoS (NPS)	Mainland China (Online)	February 2021	1295; 1295 (100)	29.3 [25.2–34.0]	A=NA; U=113 (8.7)	Lower COVID-19 hesitancy was associated with high ART adherence, paying attention to information about the COVID-19 vaccine (often, and sometimes), preference toward domestic vaccines, perceived moderate and moderately severe or severe impact of SARS-CoV-2 on immunity, waiting for vaccination programs

									organized at the workplace and unawareness of where to get COVID-19 vaccine. A higher degree of vaccine hesitancy was positively correlated with concern about the efficacy and side effects of the COVID-19 vaccine
Qi et al[60]/2021	China	CS	SbS (NPS)	Nationtion wide (Online)	May 1 to June 20, 2021	350; 335 (95.7)	36.01 (9.64)	A=280 (80); U=153 (43.7)	NA
Liu et al[33]/2021	China	CS	CoS (NPS)	Beijing Ditan Hospital, Capital Medical University	July 20 to August 20, 2021	378; 374 (98.9)	35 (8)	A=NA; U=219 (57.5)	NA
Wu et al[61]/2022	China	CS	CoS (NPS)	Wuchang district CDC (Online)	April 4 to April 18, 2021	556; 515 (92.6)	37.59 (11.45)	A=338 (60.8); U=NA	PLHIV with comorbid illnesses, those who believe that their HIV would be worse if they were infected with SARS-CoV-2, and those who believe that their ART would be affected by COVID-19 pandemic were more willing to accept COVID-19 vaccination. Higher monthly income, and being heterosexual (vs. homosexual) were associated with lower willingness to accept COVID-19 vaccination.
Vallée et al[62]/2021	France	CS	NPS	Foch Hospital, Suresnes; (Online)	January, 2021	237; 181 (76.7)	53 (NA)	A=169 (71.3); U=NA	Participant’s concerns about their health, the requirement of mandatory COVID-19 vaccination, and presence of chronic disease(s) were independently associated with a higher likelihood of vaccine acceptance. Conversely,

									participants with concerns about the serious side effects of COVID-19 vaccines, and those who believe that they are already naturally protected against COVID-19 infection were more likely to be COVID-19 vaccine-hesitant.
Menza et al[63] /2022	US	NR	NPS	Oregon	May 10, 2021	8022; 7095 (88.4)	NA	A=NA; U=4943 (61.6)	Older age, CD4 count or viral load measurement in 2020, participation in AIDS Drug Assistance Program (ADAP), and receiving Flu vaccination were associated with increased uptake. Being Hispanic/Latinx, male homosexual, injection drug user, and rural resident are associated with lower uptake.
Ekstrand et al[64]/2021	India	NR	NPS	Kamtaka state; Telephone interview	January 18 and February 19, 2021	438; (212 61.6)	39.5 (8.8)	A=270 (61.6)	Fear of vaccine side effects, having little/no confidence that vaccine is safe, having little/no confidence that vaccine protects against COVID-19, number of sources on vaccine information, and not knowing about anyone who died of COVID-19 are associated with increased odds of hesitancy.
Fulda et al[65]/2022	Multicontinent (Brazil, Botswana, Canada, Haiti, India, Peru, South Africa, Spain, Thailand, Uganda, the United	NR (secondary data analysis)	PS	Brazil (n = 1042), Botswana (n = 273), Canada (n = 123), Haiti (n = 136), India (n = 469), Peru (n = 142),	January 2021 to July 2021	6952	NR	U (Global)= 4519 (65); [United States (72), Peru (69), and Brazil (63), South Africa	Participants who received the COVID-19 vaccine were more likely to come from high-income countries; be white, male, or older; and have a high body mass index, higher atherosclerotic cardiovascular disease (ASCVD) risk score, and longer duration of ART.

	States, and Zimbabwe)			South Africa (n = 527), Spain (n = 198), Thailand (n = 582), Uganda (n = 175), the United States (n = 3162), and Zimbabwe (n = 123)				(18), Uganda (3), and Haiti (0); others=NA]	
Shrestha et al[66] /2022	US	CS	CoS (NPS)	Nationwide (Online)	December 6 2020 to January 8 2021	1030; 924 (89.7)	53.0 [60.0 −41.0]	NA	Being Black, single, politically conservative, concerned about side-effects, concerned about safety, and worried that the vaccine will not be effective are associated with a higher likelihood of hesitancy. Participants with liberal political orientation and those who were male, White, and university graduates and had a CD4 count of over 200 cells/mm3 had a lower likelihood of hesitancy.
Al-Yammahi et al[67]/2021	Ireland	RCR	NA	Beaumont Hospital **(**Onsite**)**	April 2021	40; 20 (53)	NA	A=28 (70.0); U=NA	NA
Yang et al[68]/2022	China	CS	CoS (NPS)	North (Tianjin, Beijing),	January and	2740; 2588 (94.5)	838 (30.6) are 18-	A=NA; U=170 (6.2)	Being among those prioritized to receive vaccination, receiving advice from staff supporting COVID-19 vaccination, being

				Northeast (Shenyang, Hohhot), East (Nanjing), (Nanning, Guangzhou, Shenzhen). Online	February 2021		29 years		exposed to groups supporting COVID-19 vaccination among PLHIV on the internet/social media, being cohabited/married with an opposite-sex partner, and having positive attitudes toward COVID-19 vaccination, were associated with a higher likelihood of vaccine uptake. Having negative attitudes toward COVID-19 vaccination and lacking a full-time job were negatively associated with vaccine uptake.
Mesfin et al[69]/2021	Ethiopia	CS	SyS (PS)	Butajira General Hospital (Onsite)	March 1 to April 28, 2021	398; 236 (59)	32.15 (5.1)	A=134 (33.7); U=NA	Having a chronic illness, being male, and having good knowledge about COVID-19 practice were associated with higher odds of acceptance.
Kaida et al[32]/2022	Canada	CS	NR (PS)	British Columbia (Online)	August 20-March 1, 2021	69; 3 (4.3)	49.9 (11.4)	A=45 (65.2); U=NA	Factors associated with intention to receive the COVID-19 vaccine included being older, reporting one or more chronic health condition, and being strongly influenced by direct and indirect social norms that encourage vaccination. Lack of confidence in the COVID-19 vaccine was associated with a lower likelihood of intention to vaccinate.
Iliyasu et al[70] /2022	Nigeria	CS	SyS (PS)	Aminu Kano Teaching Hospital (Onsite)	March 2021	344; 153 (44.5)	39.2 (9.68)	A=159 (46.2); U=NA	Being non-Muslim, having high-risk perception, not being worried about vaccine-related infertility rumors and believing that antiretroviral drugs protect against COVID-19 were associated with a higher likelihood of acceptance. Not being concerned about the potential effects of COVID-19-HIV co-infection was associated with a decreased likelihood of acceptance.

Chai et al[71] /2022	China	CS	ClS (PS)	Five metropolitan cities (Online)	January 2021 and February 2021	1735; 1638 (94.41)	NA	A=1013 (58.3); U=NA	NA
Jaiswal et al[72] /2022	US	CS	SRS (PS)	US/US territories (Online)	March and May 2021	496; 226 (45.6)	50.08 (0.61)	A=429 (86.5); U= 319 (64.3)	Having at least a bachelor's degree is associated with a higher likelihood of uptake. While higher levels of COVID-19 vaccine hesitancy scores are associated with a lower likelihood of willingness to accept the vaccine.
Jones et al[73] /2021	US	CS	CoS (NPS)	Miami	August to November 2020.	94; 23 (60.5)	54.4 (11.3)	A=51 (54.3); U=NA	Participants who agreed or strongly agreed that vaccines are important for health, those who agreed or strongly agreed that vaccines are effective, those who agreed or strongly agreed that vaccine-related information is trustworthy and reliable, those who agreed or strongly agreed with following doctors' recommendations regarding vaccines, and those who agreed or strongly agreed that getting vaccinated protects them from the disease had higher odds of willingness to accept the vaccine.
Zhao et al[74] /2021	China	CS	CoS (NPS)	Nationwide (Online)	7 and 23 February 2021	1618; 1541 (95.2)	NA	A=NA; U= 527 (32.6)	PLHIV with a higher level of education, those who received pneumonia or influenza vaccine in the past 3 years, those engaged in occupations with a high COVID-19 infection risk, those who believed in COVID-19 vaccine's effectiveness, and those who received COVID-19 vaccine information from the media were more likely to be vaccinated. Being concerned with adverse reactions, disclosure of participant's HIV status, and vaccine's adverse effect on HIV progression,

									as well as the presence of comorbidities, being unmarried and older age were associated with a lower likelihood of being vaccinated
Huang et al[75] /2021	China	CS	CoS (NPS)	Nationwide (Online)	January and February 2021	2570; 2431 (94.6)	NR	A=1470 (57.2); U=NA	Factors associated with a higher likelihood of acceptance included, positive attitude to COVID-19 vaccine, perceived support from valued contacts, source of information on COVID-19 vaccination (including doctors, staff of organizations offering services to PLHIV, family members and friends, and PLHIV peers). A lower likelihood of acceptance was associated with negative attitudes toward COVID-19 vaccination.
Govere-Hwenje et al[76]/2022	South Africa	CS	SRS (PS)	KwaZulu-Natal province	September 2020 and January 2021	213; 60 (28)	35 [29-43]	A=121 (57); U=NA	Older age was associated with the likelihood of acceptance. On the other hand, higher COVID-19-related mistrust and having social media as the source of information about COVID-19 were associated with a lower likelihood of acceptance.
Ortiz-Mart´ınez et al[77]/2022	Latin America	CS	CoS (NPS)	Online	February to May 2021	528; 506 (95.83)	30 (27-36)	A=460 (87.12); U=NA	NA.
Davtyan et al[78]/2022	US	CS	NPS	Coachella Valley, California (online)	September 2020 and February 2021	114; 81 (95)	62.22 (7.20)	A=86 (75); U=NA	Female and unemployed PLHIV had a lower likelihood of acceptance.
Mohamed et al[79] /2022	Egypt, Tunisia and Saudi Arabia	CS	PQS (NPS)	Egypt 227 (42%), Saudi Arabia 184	March 2021 and August 2021	540; 389 (72)	NA	A= 349 (64.6); U= 104 (19.3)	Factors associated with a higher likelihood of acceptance included being less worried to contract COVID-19 after vaccination, the belief the vaccination prevents COVID-19, and doctor's

				(34.4%), Tunisia 127 (23.5%). Online					advice to vaccinate. While residing in rural settings, the belief that ART prevents COVID-19, skepticism about the vaccine effectiveness, and having a CD4 count assessed within the previous year were associated with a lower likelihood of acceptance.
Kabir et al[80] /2022	Nigeria	CS	CoS (NPS)	Six ART clinics (2 from Kano state, and four from Yobe state)	October 11 2021 February 24 2022	763; 171 (38.18)	39.76 (10.75)	A=382 (50.1); U=103 (13.5)	Older age, male sex, unemployed, attending ART a town-located ART clinic, perceived high COVID-19 susceptibility and having a history of adverse effects of ART drug were associated with a lower likelihood of hesitancy. Conversely, compared to those attending General Hospital Gashua, participants attending the ART clinic at Infectious Disease Hospital Kano. Murtala Muhammad Specialist Hospital Kano, and Yobe State Specialist Hospital Damaturu, all located in metropolitan cities, had significantly higher odds of hesitancy.
Donohue et al[81]/2021	Ireland	CS	NA	Galway hospital (Onsite)	December 10 2020 to April 1 2021	51; 35 (68.6)	NA	A=48 (94.1); U=NA	NA
Holt et al[82]/2022	Australia	CS	NPS	Nationwide (Online)	April–June 2021	95; NA	NA	A=77 (81.1); U=42 (44.2)	NA
Muhindo et al[83]/2022	Uganda	CS	NPS	Six ART clinics (Komamb	January to April 2022	767; 282 (37)	Women:33 [28–	A=703 (91.7);	Attainment of secondary education, older age being, belief in the benefit of vaccination, belief in the safety of COVID-19 vaccines for PLHIV

				oga, Kisenyi, Kiswa, Kitebi, Kawaala, and Kasangati) in Kampala (Onsite)			40]; men: 40 [34–47]	U=534 (70.80)	and their ease of accessibility were associated with a higher likelihood of acceptance. Also, the perceived benefit of the vaccination for PLHIV, belief in the safety of the vaccine in PLHIV, ease of access, being unemployed, being female, and being tested before for COVID-19 are associated with a higher likelihood of uptake.
Swendeman et al[84]/2022	US	CS	NPS	Los Angeles and New Orleans (Online)	October 2020	52; NA	NA	A=32 (61.5); U=NA	
Wickersham et al[85]/2022	US	CS	NPS	Nationwide (Online)	December 6 2020 to January 8 2021	1030; 924 (89.7)	50.7 (12.5)	A=858 (83.8); U=NA	Being black, politically conservative, low income were associated with a lower likelihood of acceptance. On the other hand, being vaccinated annually against influenza, and being politically liberal were associated with a higher willingness to accept the vaccine.
Prestage et al[86]/2022	Australia	CS	CoS (NPS)	Nationwide (Online)	January 17 2021 to June 22 2021	57; NA	NA	A=NA; U=35 (61.4);	NA
Siewe Fodjo et al[87]/2021	Multicontinent	CS	NPS	Multinational (Online)	July 20-November 30 2020	247; NA	44.5 (13.2)	A=188 (76.1); U=NA	NA

Folayan et al[88]/2022	Nigeria	CS	SbS (NPS)	Adamawa, Akwa Ibom, Anambra, Benue, Kaduna, Lagos, Enugu, Gombe, Nassarawa and Niger (Online/Onsite)	June to October 2021	2024; NA	NA	A=1621 (80.1); U=NA	NA
Bogart et al[89]/2022	US	CS	CoS (NPS)	Los Angeles County, California (Onsite)	May to July, 2020	101; 88 (87)	50.3 (11.5)	A=69 (78.0); U=NA	NA
Lyons et al[90]/2023	Trinidad & Tobago	CS	CoS (NPS)	Medical Research Foundation of Trinidad and Tobago (MRFTT) (Onsite)	August and September 2021	272; 135 (49.6)	41 (13.0)	A=163 (59.9); U=NA	Bein females, and having a mixed ethnicity were associated with lower acceptance. On the other hand, Indo-Trinidadians, non-heterosexuals, and being confident in the COVID-19 vaccine were associated with higher acceptance.
Madzima et al[91]/2022	Zimbabwe	CS	StS (PS)	Nationwide (Onsite)	October 2020 to	480; 142 (32.49)	NA	A=NA; U=280 (64.07)	NA

					November 2021.				
Chaudhuri et al[92]/2022	India	CS	SnS (NPS)	Chittoor district of Andhra Pradesh (Online/on site)	Septembe r to October 2021	247; 106 (42.9)	40 (11)	A=218 (88.26); U=195 (78.95)	NA
Bert et al[93]/2022	Italy	CS	NPS	Amedeo di Savoia Hospital in Turin (Onsite)	November 2021 to April 2022	160; 133 (83.1)	49.97 (11.82)	A=149 (93.13); U=137 (86.2)	NA
Shallangwa et al[94]/2023	Nigeria	CS	NPS	State Specialist Hospital Maiduguri (Onsite)	January 4 to February 25 2022	344; 102 (29.7)	NA	A=115 (33.4); U=88 (26.6)	No significant relationship
Mchawa et al[95]/2023	Malawi	CS	CoS (NPS)	Mpemba health center in Traditional Authority Somba, in Blantyre district (Onsite)	May to Septembe r 2022	341; 142 (41.6	NA	A=150 (43.99); U=NA	Having higher levels of knowledge, good attitude and trust in the COVID-19 vaccination were negatively associated with hesitancy.

Sun et al[96]/2022	China	CS	NPS	PLA General Hospital (Onsite)	June to December 2021	169; NA	NA	A=102 (60.36); U=NA	Age of the participants vaccine safety were associated with intention to vaccinate against COVID-19.
Singh et al[97]/2023	India	CS	CsS (NPS)	Northe India (Onsite)	May and June 2021	300; 180 (59.33)	40	A=60 (20.0); U=NA	NA
Lv et al[98]/2023	China	CS	NPS	Mainland China (Online)	January 2022 to March 2022	1424; 1295 (90.9)	NA	A=1316 (92.42); U=1258 (88.34)	Being older, having lower academic level, having comorbidity, having lower CD4+ count, having higher levels of anxiety and despair, and high risk perception were associated with higher levels of COVID-19 vaccine hesitancy. Also, lower vaccine uptake were associated with having lower level of education, lower CD4 count, and having higher levels of anxiety and depression.
Ogaz et al[99]/2023	UK	CS	NPS	Nationwid e (Online)	November to December 2021	120; NA	NA	A=NA;U= 114 (95.0)	NA
Rosenthal et al[100]/2023	US	RC	NR	New York (Oline RCR)	Through March 2022	101,205 ; 70,636 (69.8%)	NA	A=NA; U=72950 (72.08)	NA
Hechter et al[101]/2023	US	RC	NR	California, Colorado, Washingto , Denver (Online RCR)	December 1 to December 31 2021	22,063; NA	NA	A=NA; U= 19,966 (90.5)	Being male, being Asian, and being vaccinated against influenza in the previous two years were associated with higher COVID-19 vaccination. While PLHIV having a CD4 count <200, and those who have failed to achieve viral suppression had lower vaccination.

Baker et al[102]/2023	US	PC	NR	Maryland (Online RCR)	April 6 2021 to February 22 2022	960; NA	NA	A=NA; U=670 (70.0)	Being male, belonging to Black race, and HIV viral suppression (<400) were associated with higher COVID-19 vaccination completion. While being over 50 years was associated with lower completion.
Wicaksana et al[103]/2023	Indonesia	CS	SRS (PS)	Jakarta (Cipto Mangunkusumo Hospital, Dharmais National Cancer Hospital, and Kramat 128 Hospital) (Online and Onsite)	September to December 2021	470; 358 (76.2)	NA	A=355; U=NA	Determinants of intention to vaccinate include instrumental attitude, perceived behavioral control, and subjective norm.
Strathdee et al[104]/2023	US	Cohort	NPS	San Diego, California (Onsite)	October 2020 to September 2021 and through March 15, 2022	15; NA	NA	A=NA; U=8	NA

Javanbakht et al[105]/2023	US	Cohort	CoS (NPS)	Baltimore, Chicago, Los Angeles, Miami, and Vancouver (Online)	May 2021 to January 2022	777; NA	NA	A=683; U=677	NA
Costiniuk et al[106]/2023	Canada	CS	NPS	Nationwide (Online)	February to May 2022	246; 189 (76.8)	46.8 (14.0)	A=NA; U=219 (84.6)	Age per ten-year increase and higher level of education were associated with vaccination.
Cummings et al[107]/2023	Sierra Leone	CS	CoS (NPS)	Connaught Hospital in Freetown (Onsite)	April to June 2022	490; 140 (28.6)	38 (32-49)	A=NA; U=85	PLHIV who are Muslim and those residing in urban areas had higher COVID-19 vaccine hesitancy. While a past history of COVID-19 testing was associated with lower hesitancy.
Tunta et al[108]/2023	Ethiopia	CS	CsS (NPS)	Woldia comprehensive specialized hospital located, Northern Ethiopia. (Onsite)	February 15 to March 15, 2022	332; 128 (38.55)	NA	A=220 (66.3); U=NA	Having poor knowledge and negative attitude toward COVID-19 was significantly associated with vaccine refusal. And rural residence was associated with lower likelihood of willingness to vaccinate against the COVID-19.

Keys: ART, Antiretroviral therapy; CC, Case-Control; ClS, Clustered sampling; CoS, Convenient sampling; CsS, Consecutive Sampling; NA, Not available; NPS, Non-probability sampling; NS, Not Reported; PC, Prospective Cohort; PS, Probability sampling; PQS, Purposive quota sampling; RC, Retrospective Cohort; RCR, Retrospective charts review; SnS, Snowball sampling; SRS, Simple random sampling; StS, Stratified sampling; SyS, Systematic sampling.

References (Numbered as within the main manuscript file)

32. Kaida A, Brotto LA, Murray MCM, et al. Intention to Receive a COVID-19 Vaccine by HIV Status Among a Population-Based Sample of Women and Gender Diverse Individuals in British Columbia, Canada. *AIDS Behav*. 2022;26(7):2242-2255. doi:10.1007/s10461-022-03577-w
33. Liu Y, Han J, Li X, et al. COVID-19 Vaccination in People Living with HIV (PLWH) in China: A Cross Sectional Study of Vaccine Hesitancy, Safety, and Immunogenicity. *Vaccines*. 2021;9(12):1458. doi:10.3390/vaccines9121458
57. Su J, Jia Z, Wang X, et al. Acceptance of COVID-19 vaccination and influencing factors among people living with HIV in Guangxi, China: a cross-sectional survey. *BMC Infect Dis*. 2022;22(1):471. doi:10.1186/s12879-022-07452-w
58. Buckley M, Shayani K, Spier D, Stefanov D, Ahmadi L. Attitudes Towards COVID-19 Vaccination in People Living with HIV (PLWHIV) at an Urban Primary Care Clinic. Published online May 23, 2022. Accessed June 16, 2022. https://papers.ssrn.com/abstract=4113173
59. Zheng W, Sun Y, Li H, et al. COVID-19 vaccine uptake and hesitancy among HIV-infected men who have sex with men in mainland China: a cross-sectional survey. *Hum Vaccines Immunother*. 2021;17(12):4971-4981. doi:10.1080/21645515.2021.1996152
60. Qi L, Yang L, Ge J, Yu L, Li X. COVID-19 Vaccination Behavior of People Living with HIV: The Mediating Role of Perceived Risk and Vaccination Intention. *Vaccines*. 2021;9(11):1288. doi:10.3390/vaccines9111288
61. Wu S, Ming F, Xing Z, et al. COVID-19 Vaccination Willingness Among People Living With HIV in Wuhan, China. *Front Public Health*. 2022;10:883453. doi:10.3389/fpubh.2022.883453
62. Vallée A, Fourn E, Majerholc C, Touche P, Zucman D. COVID-19 Vaccine Hesitancy among French People Living with HIV. *Vaccines*. 2021;9(4):302. doi:10.3390/vaccines9040302
63. Menza TW, Capizzi J, Zlot AI, Barber M, Bush L. COVID-19 Vaccine Uptake Among People Living with HIV. *AIDS Behav*. 2022;26(7):2224-2228. doi:10.1007/s10461-021-03570-9
64. Ekstrand ML, Heylen E, Gandhi M, Steward WT, Pereira M, Srinivasan K. COVID-19 Vaccine Hesitancy Among PLWH in South India: Implications for Vaccination Campaigns. *J Acquir Immune Defic Syndr 1999*. 2021;88(5):421-425. doi:10.1097/QAI.0000000000002803
65. Fulda ES, Fitch KV, Overton ET, et al. COVID-19 Vaccination Rates in a Global HIV Cohort. *J Infect Dis*. 2022;225(4):603-607. doi:10.1093/infdis/jiab575
66. Shrestha R, Meyer JP, Shenoi S, et al. COVID-19 Vaccine Hesitancy and Associated Factors among People with HIV in the United States: Findings from a National Survey. Vaccines 2022, 10, 424. Published online 2022.
67. Al-Yammahi A, Reynolds B, Crawford R, et al. Covid-19 Vaccine Uptake in People Living with HIV. :2.
68. Yang J, Yu M, Fu G, et al. COVID-19 Vaccination Uptake Among a Nationwide Sample of People Living With HIV During the Early Phase of Vaccine Rollout in China. *Front Med*. 2022;9:822680. doi:10.3389/fmed.2022.822680
69. Mesfin Y, Argaw M, Geze S, Zewdu BT. Factors Associated with Intention to Receive COVID-19 Vaccine Among HIV Positive

Patients Attending ART Clinic in Southwest Ethiopia. *Patient Prefer Adherence*. 2021;15:2731-2738. doi:10.2147/PPA.S342801
70. Iliyasu Z, Kwaku AA, Umar AA, et al. Predictors of COVID-19 Vaccine Acceptability among Patients Living with HIV in Northern Nigeria: A Mixed Methods Study. *Curr HIV Res*. 2022;20(1):82-90. doi:10.2174/1570162X19666211217093223
71. Chai R, Yang J, Zhang X, et al. Reasons for COVID-19 Vaccine Hesitancy Among Chinese People Living With HIV/AIDS: Structural Equation Modeling Analysis. *JMIR Public Health Surveill*. 2022;8(6):e33995. doi:10.2196/33995
72. Jaiswal J, Krause KD, Martino RJ, et al. SARS-CoV-2 Vaccination Hesitancy and Behaviors in a National Sample of People Living with HIV. *AIDS Patient Care STDs*. 2022;36(1):34-44. doi:10.1089/apc.2021.0144
73. Jones DL, Salazar AS, Rodriguez VJ, et al. Severe Acute Respiratory Syndrome Coronavirus 2: Vaccine Hesitancy Among Underrepresented Racial and Ethnic Groups With HIV in Miami, Florida. *Open Forum Infect Dis*. 2021;8(6):ofab154. doi:10.1093/ofid/ofab154
74. Zhao H, Wang H, Li H, et al. Uptake and adverse reactions of COVID-19 vaccination among people living with HIV in China: a case–control study. *Hum Vaccines Immunother*. 2021;17(12):4964-4970. doi:10.1080/21645515.2021.1991183
75. Huang X, Yu M, Fu G, et al. Willingness to Receive COVID-19 Vaccination Among People Living With HIV and AIDS in China: Nationwide Cross-sectional Online Survey. *JMIR Public Health Surveill*. 2021;7(10):e31125. doi:10.2196/31125
76. Govere-Hwenje S, Jarolimova J, Yan J, et al. Willingness to accept COVID-19 vaccination among people living with HIV in a high HIV prevalence community. *BMC Public Health*. 2022;22(1):1239. doi:10.1186/s12889-022-13623-w
77. Ortiz-Martínez Y, López-López MÁ, Ruiz-González CE, et al. Willingness to receive COVID-19 vaccination in people living with HIV/AIDS from Latin America. *Int J STD AIDS*. 2022;33(7):652-659. doi:10.1177/09564624221091752
78. Davtyan M, Frederick T, Taylor J, Christensen C, Brown BJ, Nguyen AL. Determinants of COVID-19 vaccine acceptability among older adults living with HIV. *Medicine (Baltimore)*. 2022;101(31):e29907. doi:10.1097/MD.0000000000029907
79. Mohamed R, White TM, Lazarus JV, et al. COVID-19 vaccine acceptance and associated factors among people living with HIV in the Middle East and North Africa region. *South Afr J HIV Med*. 2022;23(1):1391. doi:10.4102/sajhivmed.v23i1.1391
80. Kabir Sulaiman S, Sale Musa M, Isma'il Tsiga-Ahmed F, et al. COVID-19 vaccine hesitancy among people living with HIV in a low-resource setting: A multi-center study of prevalence, correlates and reasons. *Vaccine*. 2023;41(15):2476-2484. doi:10.1016/j.vaccine.2023.02.056
81. Donohue S, Boyle N, Holmes A. A Survey of the Potential Acceptance of a SARS-CoV-2 Vaccine Amongst People Living with HIV. *Ir Med J*. 114(9):P477.
82. Holt M, MacGibbon J, Bavinton B, et al. COVID-19 Vaccination Uptake and Hesitancy in a National Sample of Australian Gay and Bisexual Men. *AIDS Behav*. 2022;26(8):2531-2538. doi:10.1007/s10461-022-03603-x
83. Muhindo R, Okoboi S, Kiragga A, King R, Arinaitwe WJ, Castelnuovo B. COVID-19 vaccine acceptability, and uptake among people living with HIV in Uganda. *PLOS ONE*. 2022;17(12):e0278692. doi:10.1371/journal.pone.0278692
84. Swendeman D, Norwood P, Saleska J, et al. Vaccine Attitudes and COVID-19 Vaccine Intentions and Prevention Behaviors among Young People At-Risk for and Living with HIV in Los Angeles and New Orleans. *Vaccines*. 2022;10(3):413.

doi:10.3390/vaccines10030413
85. Wickersham JA, Meyer JP, Shenoi S, et al. Willingness to Be Vaccinated Against COVID-19 Among People With HIV in the United States: Results From a National Survey. *Front Med*. 2022;9:886936. doi:10.3389/fmed.2022.886936
86. Prestage G, Storer D, Jin F, et al. COVID-19 Vaccine Uptake and Its Impacts in a Cohort of Gay and Bisexual Men in Australia. *AIDS Behav*. 2022;26(8):2692-2702. doi:10.1007/s10461-022-03611-x
87. Siewe Fodjo JN, Faria de Moura Villela E, Van Hees S, Vanholder P, Reyntiens P, Colebunders R. Follow-Up Survey of the Impact of COVID-19 on People Living with HIV during the Second Semester of the Pandemic. *Int J Environ Res Public Health*. 2021;18(9):4635. doi:10.3390/ijerph18094635
88. Folayan MO, Arije O, Enemo A, et al. Associations between COVID-19 vaccine hesitancy and the experience of violence among women and girls living with and at risk of HIV in Nigeria. *Afr J AIDS Res*. 2022;0(0):1-11. doi:10.2989/16085906.2022.2118615
89. Bogart LM, Ojikutu BO, Tyagi K, et al. COVID-19 Related Medical Mistrust, Health Impacts, and Potential Vaccine Hesitancy Among Black Americans Living With HIV. *J Acquir Immune Defic Syndr 1999*. 2021;86(2):200-207. doi:10.1097/QAI.0000000000002570
90. Lyons N, Bhagwandeen B, Edwards J. Factors Affecting COVID-19 Vaccination Intentions among Patients Attending a Large HIV Treatment Clinic in Trinidad Using Constructs of the Health Belief Model. *Vaccines*. 2023;11(1):4. doi:10.3390/vaccines11010004
91. Madzima B, Makoni T, Mugurungi O, et al. The impact of the COVID-19 pandemic on people living with HIV in Zimbabwe. *Afr J AIDS Res*. 2022;21(2):194-200. doi:10.2989/16085906.2022.2103004
92. Chaudhuri. Vaccination against COVID-19 among people living with HIV: A cross-sectional survey in Chittoor District of Andhra Pradesh, India. Accessed June 16, 2022. https://www.cmijournal.org/article.asp?issn=0973-4651;year=2022;volume=20;issue=1;spage=32;epage=36;aulast=Chaudhuri
93. Bert F, Pivi A, Russotto A, et al. COVID-19 Vaccination among HIV+ Patients: An Italian Cross-Sectional Survey. *Vaccines*. 2022;10(9):1438. doi:10.3390/vaccines10091438
94. Shallangwa MM, Musa SS, Iwenya HC, Manirambona E, Iii DELP, Tukur BM. Assessment of COVID-19 vaccine hesitancy among people living with HIV/AIDS: a single-centered study. *PAMJ - One Health*. 2023;10(2). doi:10.11604/pamj-oh.2023.10.2.37945
95. Mchawa C, Zhang SS, Zhang WX, et al. Assessing SARS-CoV-2 vaccine hesitancy among the people living with and without HIV from May to September 2022 in Blantyre, Malawi. *Hum Vaccines Immunother*. 2023;0(0):2222052. doi:10.1080/21645515.2023.2222052
96. Sun YB. Awareness towards COVID-19 prevention and factors related to the willingness to receive COVID-19 vaccine among people living with HIV/AIDS. *Zhongguo Bingdubing Zazhi Chin J Viral Dis*. Published online 2022:349-349.
97. Singh AD, Upal N, Oberoi S, et al. Awareness and Hesitancy of COVID-19 and other Vaccines among People Living with HIV/AIDS Attending Antiretroviral Therapy (ART) Center in North India. *Indian J Community Med*. 2023;48(3):492.

doi:10.4103/ijcm.ijcm_901_22
98. Lv X, Zhao C, Song B, et al. COVID-19 vaccination in people living with HIV and AIDS (PLWHA) in China: A cross-sectional study. *Hum Vaccines Immunother*. 2023;0(0):2151798. doi:10.1080/21645515.2022.2151798
99. Ogaz D, Allen H, Reid D, et al. COVID-19 infection and vaccination uptake in men and gender-diverse people who have sex with men in the UK: analyses of a large, online community cross-sectional survey (RiiSH-COVID) undertaken November–December 2021. *BMC Public Health*. 2023;23(1):829. doi:10.1186/s12889-023-15779-5
100. Rosenthal EM, Patterson W, Chicoine J, et al. COVID-19 Vaccination and Hospitalization Among Persons Living With Diagnosed HIV in New York State. *J Acquir Immune Defic Syndr 1999*. 2023;93(2):92-100. doi:10.1097/QAI.0000000000003177
101. Hechter R, Qian L, Amy Liu IL, et al. COVID-19 Vaccine Coverage Among People With HIV in the Vaccine Safety Datalink. *Top Antivir Med*. Published online 2023:406-407.
102. Baker P, Cepeda J, Schluth C, et al. Time-to-Completion of Covid-19 Vaccination Primary Series Varies by HIV Viral Load Status Among People Who Inject Drugs in Baltimore, Maryland. Published online May 16, 2023. doi:10.2139/ssrn.4423541
103. Wicaksana B, Yunihastuti E, Shatri H, et al. Predicting Intention to Receive COVID-19 Vaccination in People Living with HIV using an Integrated Behavior Model. *Vaccines*. 2023;11(2):296. doi:10.3390/vaccines11020296
104. Strathdee SA, Abramovitz D, Vera CF, et al. Predictors of COVID-19 vaccine uptake among people who inject drugs. *Vaccine*. 2023;41(12):1916-1924. doi:10.1016/j.vaccine.2023.01.042
105. Javanbakht M, Khan L, Mustanski B, et al. Substance use and other factors associated with COVID-19 vaccine uptake among people at risk for or living with HIV: Findings from the C3PNO consortium. *Prev Med Rep*. 2023;35:102300. doi:10.1016/j.pmedr.2023.102300
106. Costiniuk CT, Singer J, Needham J, et al. Understanding COVID-19 Vaccine Confidence in People Living with HIV: A pan-Canadian Survey. *AIDS Behav*. 2023;27(8):2669-2680. doi:10.1007/s10461-023-03991-8
107. Cummings PE, Lakoh S, Yendewa SA, et al. Understanding COVID-19 Vaccine Uptake and Hesitancy Among People With HIV in Freetown, Sierra Leone: A Cross-sectional Study. Published online May 16, 2023:2023.05.11.23289882. doi:10.1101/2023.05.11.23289882
108. Tunta A, Shiferaw M, Mesele H, Kassahun W, Yehualaw F, Abera A. Willingness to take COVID-19 Vaccination among People Living with HIV/AIDS on Anti-Retroviral Therapy and Associated Factors in Woldia Comprehensive Specialized Hospital, Woldia, EthiopiaTanzania. *Int J Sex Transm Dis*. 2023;1(1):28-39.

SECTION B

Results of meta-analysis of COVID-19 vaccine acceptance and uptake using all studies

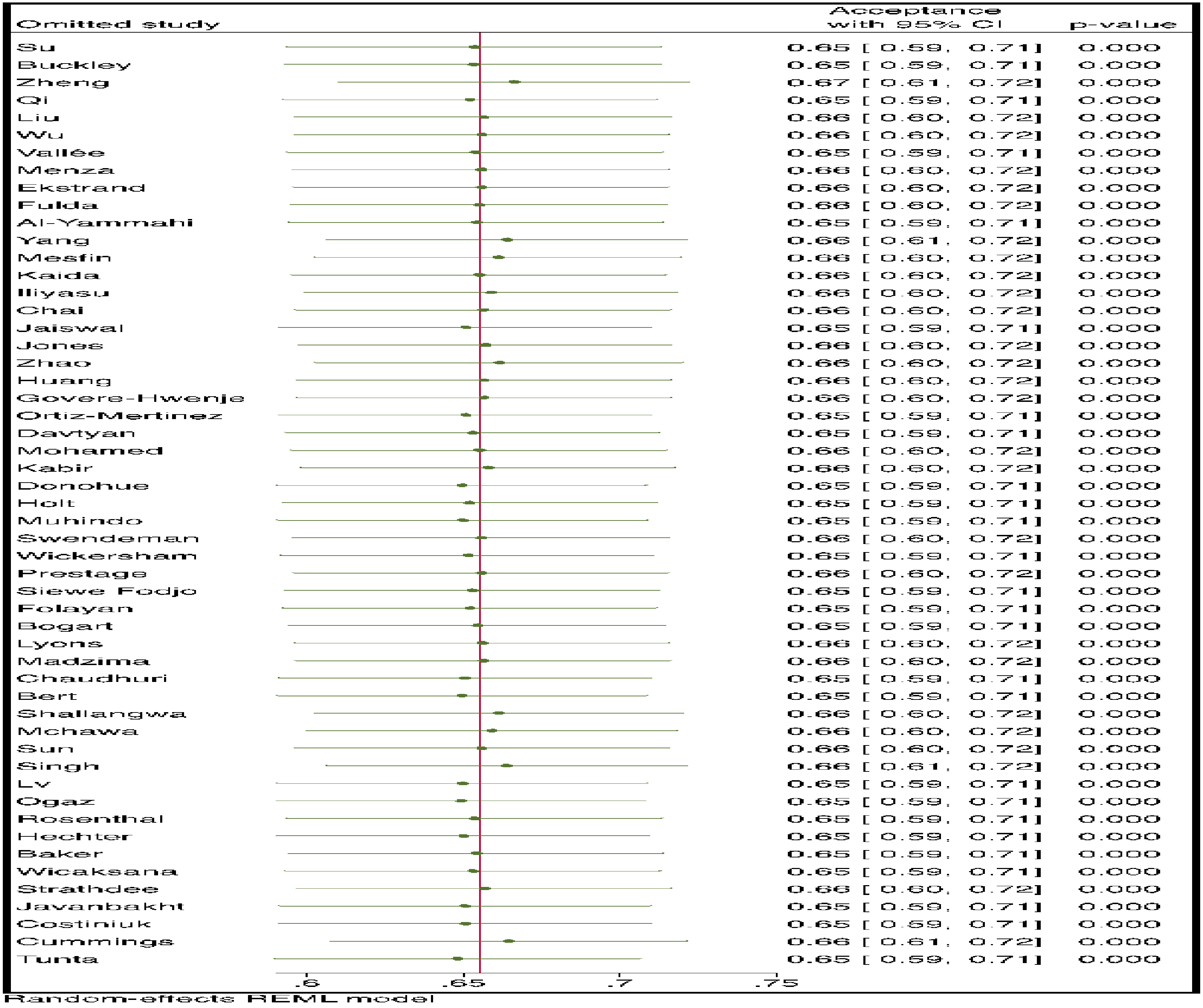
Omitted study
Acceptance with 95% CI
p-value
Su 0.65 [0.59, 0.71] 0.000
Buckley 0.65 [0.59, 0.71] 0.000
Zheng 0.67 [0.61, 0.72] 0.000
Qi 0.65 [0.59, 0.71] 0.000
Liu 0.66 [0.60, 0.72] 0.000
Wu 0.66 [0.60, 0.72] 0.000
Vallée 0.65 [0.59, 0.71] 0.000
Menza 0.66 [0.60, 0.72] 0.000
Ekstrand 0.66 [0.60, 0.72] 0.000
Fulda 0.66 [0.60, 0.72] 0.000
Al-Yammahi 0.65 [0.59, 0.71] 0.000
Yang 0.66 [0.61, 0.72] 0.000
Mesfin 0.66 [0.60, 0.72] 0.000
Kaida 0.66 [0.60, 0.72] 0.000
Iliyasu 0.66 [0.60, 0.72] 0.000
Chai 0.66 [0.60, 0.72] 0.000
Jaiswal 0.65 [0.59, 0.71] 0.000
Jones 0.66 [0.60, 0.72] 0.000
Zhao 0.66 [0.60, 0.72] 0.000
Huang 0.66 [0.60, 0.72] 0.000
Govere-Hwenje 0.66 [0.60, 0.72] 0.000
Ortiz-Mertinez 0.65 [0.59, 0.71] 0.000
Davtyan 0.65 [0.59, 0.71] 0.000
Mohamed 0.66 [0.60, 0.72] 0.000
Kabir 0.66 [0.60, 0.72] 0.000
Donohue 0.65 [0.59, 0.71] 0.000
Holt 0.65 [0.59, 0.71] 0.000
Muhindo 0.65 [0.59, 0.71] 0.000
Swendeman 0.66 [0.60, 0.72] 0.000
Wickersham 0.65 [0.59, 0.71] 0.000
Prestage 0.66 [0.60, 0.72] 0.000
Siewe Fodjo 0.65 [0.59, 0.71] 0.000
Folayan 0.65 [0.59, 0.71] 0.000
Bogart 0.65 [0.59, 0.71] 0.000
Lyons 0.66 [0.60, 0.72] 0.000
Madzima 0.66 [0.60, 0.72] 0.000
Chaudhuri 0.65 [0.59, 0.71] 0.000
Bert 0.65 [0.59, 0.71] 0.000
Shallangwa 0.66 [0.60, 0.72] 0.000
Mchawa 0.66 [0.60, 0.72] 0.000
Sun 0.66 [0.60, 0.72] 0.000
Singh 0.66 [0.61, 0.72] 0.000
Lv 0.65 [0.59, 0.71] 0.000
Ogaz 0.65 [0.59, 0.71] 0.000
Rosenthal 0.65 [0.59, 0.71] 0.000
Hechter 0.65 [0.59, 0.71] 0.000
Baker 0.65 [0.59, 0.71] 0.000
Wicaksana 0.65 [0.59, 0.71] 0.000
Strathdee 0.66 [0.60, 0.72] 0.000
Javanbakht 0.65 [0.59, 0.71] 0.000
Costiniuk 0.65 [0.59, 0.71] 0.000
Cummings 0.66 [0.61, 0.72] 0.000
Tunta 0.65 [0.59, 0.71] 0.000
.6
.65
.7
.75
Random-effects REML model

Supplementary Figure. 1: Forest plot of sensitivity analyses for each study reporting the acceptance rate of the COVID-19 vaccine among PLHIV using random effects model meta-analysis. Each solid square represents the effect size of each characteristic, while the ends of the adjoining horizontal lines represent lower (left) and upper (right) confidence intervals. (n = 53 studies, N = 166,455 participants).

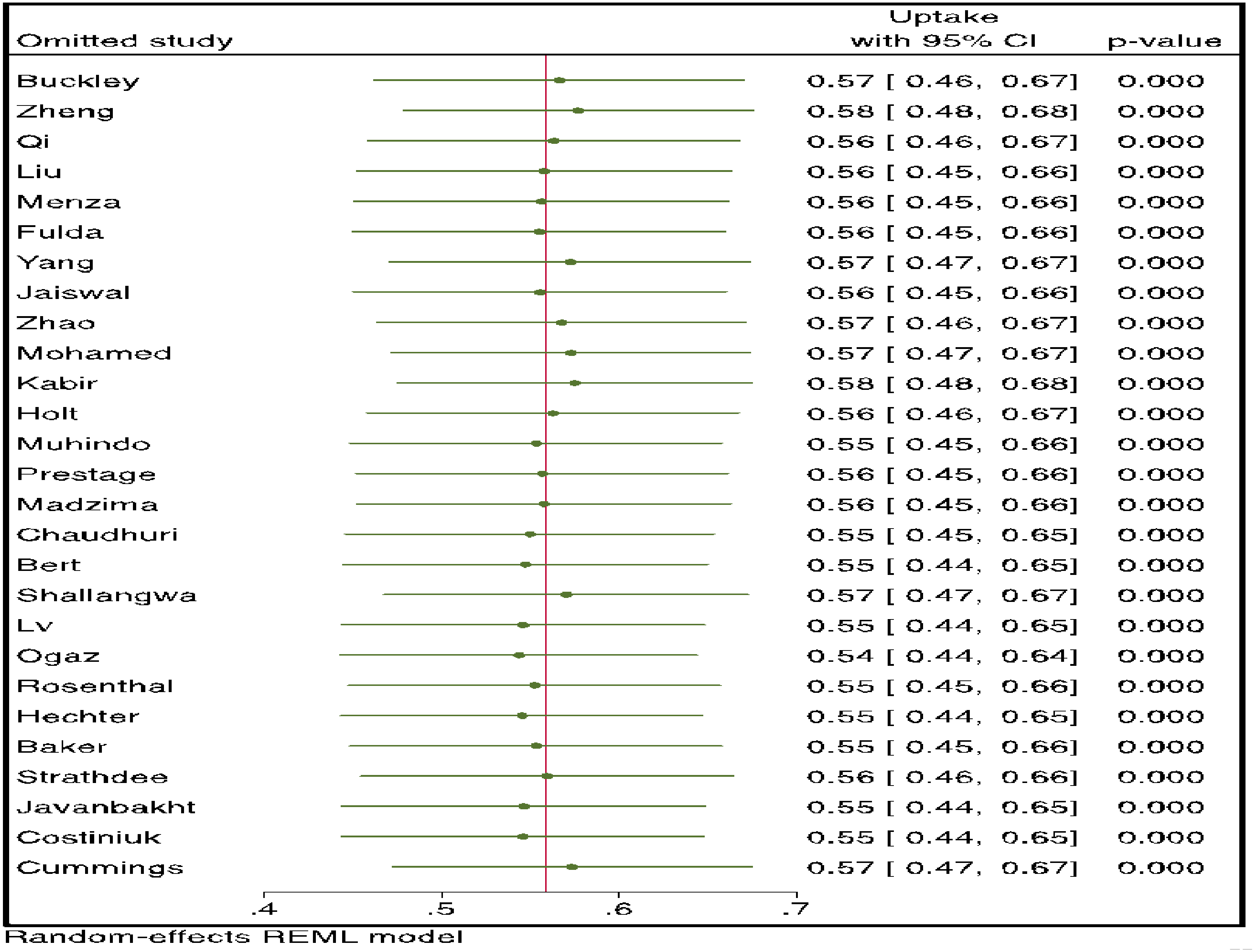

Omitted study
Uptake with 95% CI
p-value
Buckley 0.57 [0.46, 0.67] 0.000
Zheng 0.58 [0.48, 0.68] 0.000
Qi 0.56 [0.46, 0.67] 0.000
Liu 0.56 [0.45, 0.66] 0.000
Menza 0.56 [0.45, 0.66] 0.000
Fulda 0.56 [0.45, 0.66] 0.000
Yang 0.57 [0.47, 0.67] 0.000
Jaiswal 0.56 [0.45, 0.66] 0.000
Zhao 0.57 [0.46, 0.67] 0.000
Mohamed 0.57 [0.47, 0.67] 0.000
Kabir 0.58 [0.48, 0.68] 0.000
Holt 0.56 [0.46, 0.67] 0.000
Muhindo 0.55 [0.45, 0.66] 0.000
Prestage 0.56 [0.45, 0.66] 0.000
Madzima 0.56 [0.45, 0.66] 0.000
Chaudhuri 0.55 [0.45, 0.65] 0.000
Bert 0.55 [0.44, 0.65] 0.000
Shallangwa 0.57 [0.47, 0.67] 0.000
Lv 0.55 [0.44, 0.65] 0.000
Ogaz 0.54 [0.44, 0.64] 0.000
Rosenthal 0.55 [0.45, 0.66] 0.000
Hechter 0.55 [0.44, 0.65] 0.000
Baker 0.55 [0.45, 0.66] 0.000
Strathdee 0.56 [0.46, 0.66] 0.000
Javanbakht 0.55 [0.44, 0.65] 0.000
Costiniuk 0.55 [0.44, 0.65] 0.000
Cummings 0.57 [0.47, 0.67] 0.000
.4
.5
.6
.7
Random-effects REML model

Supplementary Figure 2: Forest plot of sensitivity analyses for each study reporting the uptake rate of the COVID-19 vaccine among PLHIV using random effects model meta-analysis. Each solid square represents the effect size of each characteristic, while the ends of the adjoining horizontal lines represent lower (left) and upper (right) confidence intervals (n = 27 studies, N = 150,926 participants).

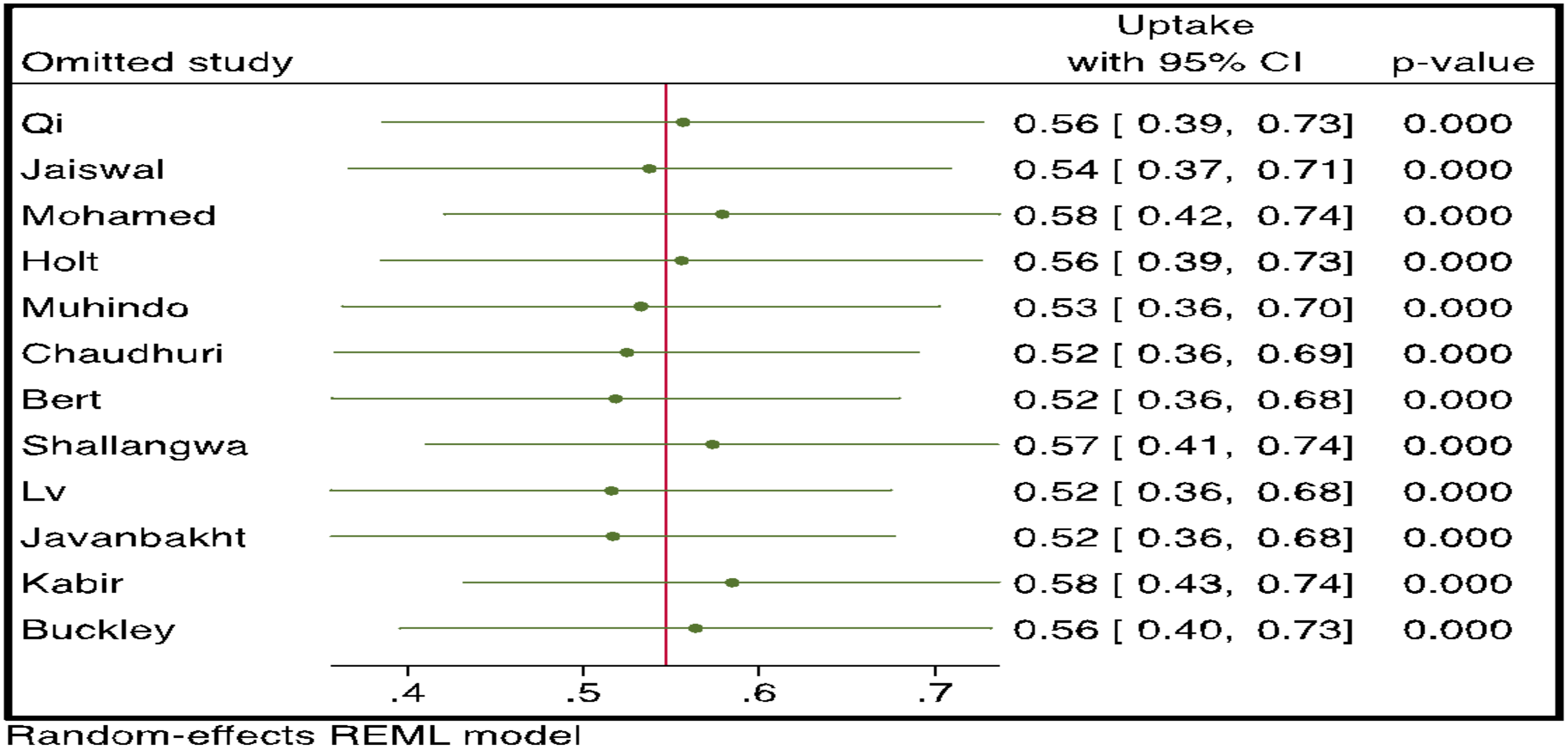

Supplementary Figure 3: Forest plot of sensitivity analyses for each study reporting the uptake rate of the COVID-19 vaccine among PLHIV who indicated acceptance using random effects model meta-analysis. Each solid square represents the effect

size of each characteristic, while the ends of the adjoining horizontal lines represent lower (left) and upper (right) confidence intervals (n = 13 studies, N = 6,564 participants).

.

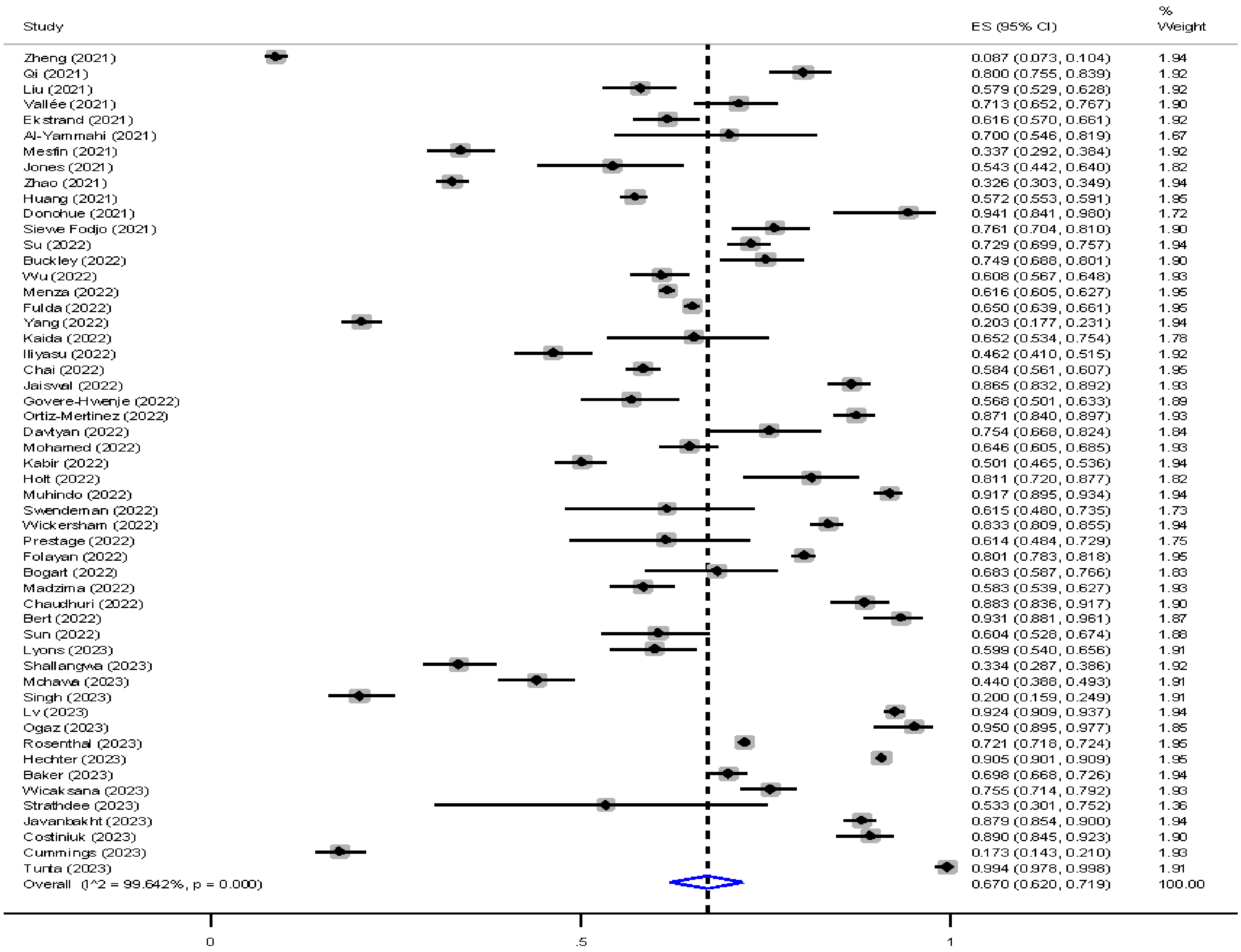

COVID-19 Vaccine Acceptance Among PLHIV (Globally)

Supplementary Figure 4: Forest plots of the results of random-effects model meta-analysis of the prevalence (%) of COVID-19 vaccine acceptance globally (n = 53 studies, N = 166,455 participants) pooled using inverse variance weights. Each black-colored solid square represents the effect size of each characteristic, while the ends of the adjoining horizontal lines represent lower (left) and upper (right) confidence intervals. The blue-colored hollow diamond at the bottom denotes the overall estimated effect size and the 95% CI. All statistics were based on a two-sided t-test. ES, Effect Size.

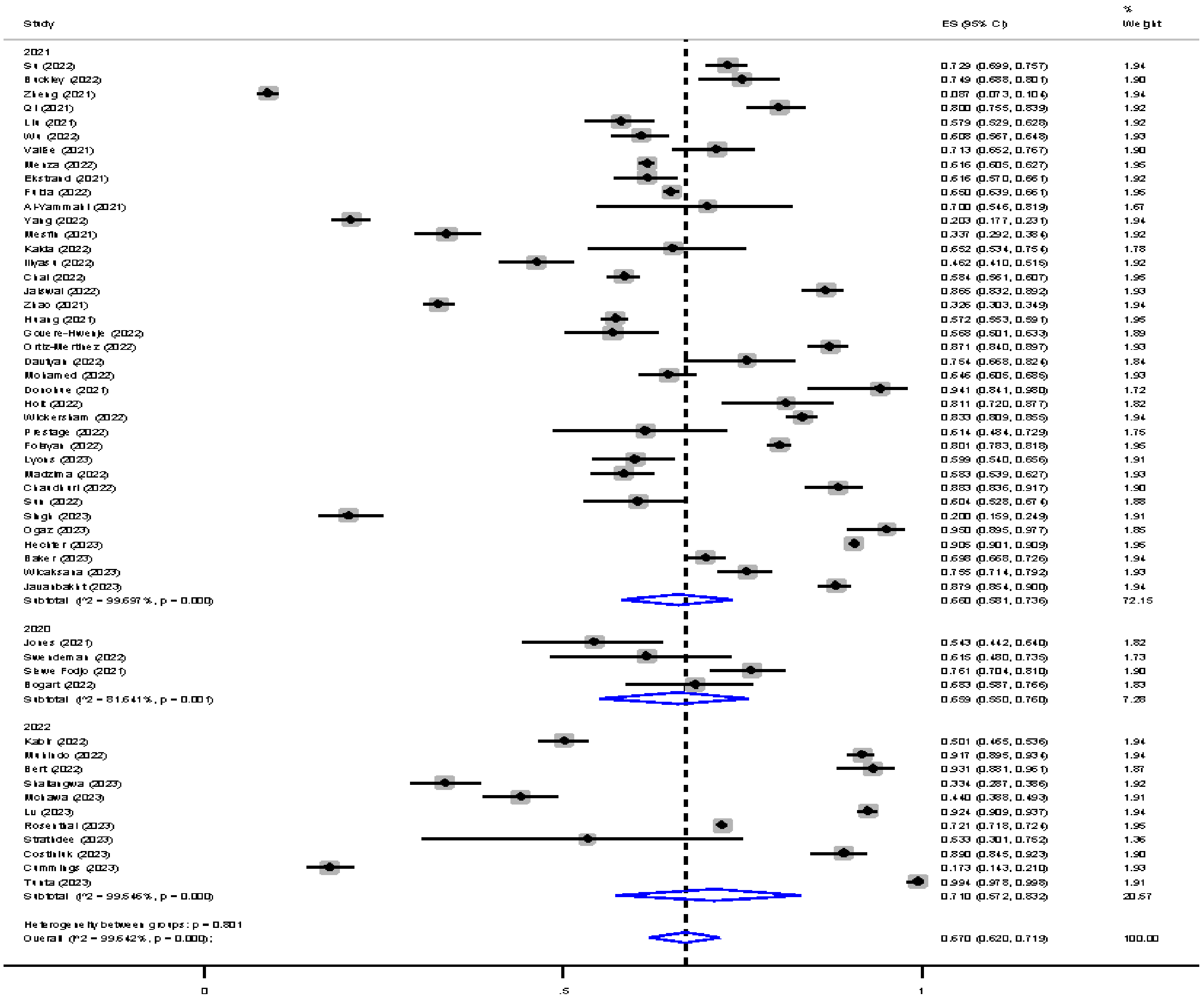

COVID-19 Vaccine Acceptance Among PLHIV (By Study Year)

Supplementary Figure 5: Forest plots of the results of random-effects model meta-analysis of the prevalence (%) of COVID-19 vaccine acceptance according to the study (n = 53 studies, N = 166,455 participants) pooled using inverse variance weights. Each black-colored solid square represents the effect size of each characteristic, while the ends of the adjoining horizontal lines represent lower (left) and upper (right) confidence intervals. The blue-colored hollow diamond at the bottom denotes the overall estimated effect size and the 95% CI. All statistics were based on a two-sided t-test. ES, Effect Size.

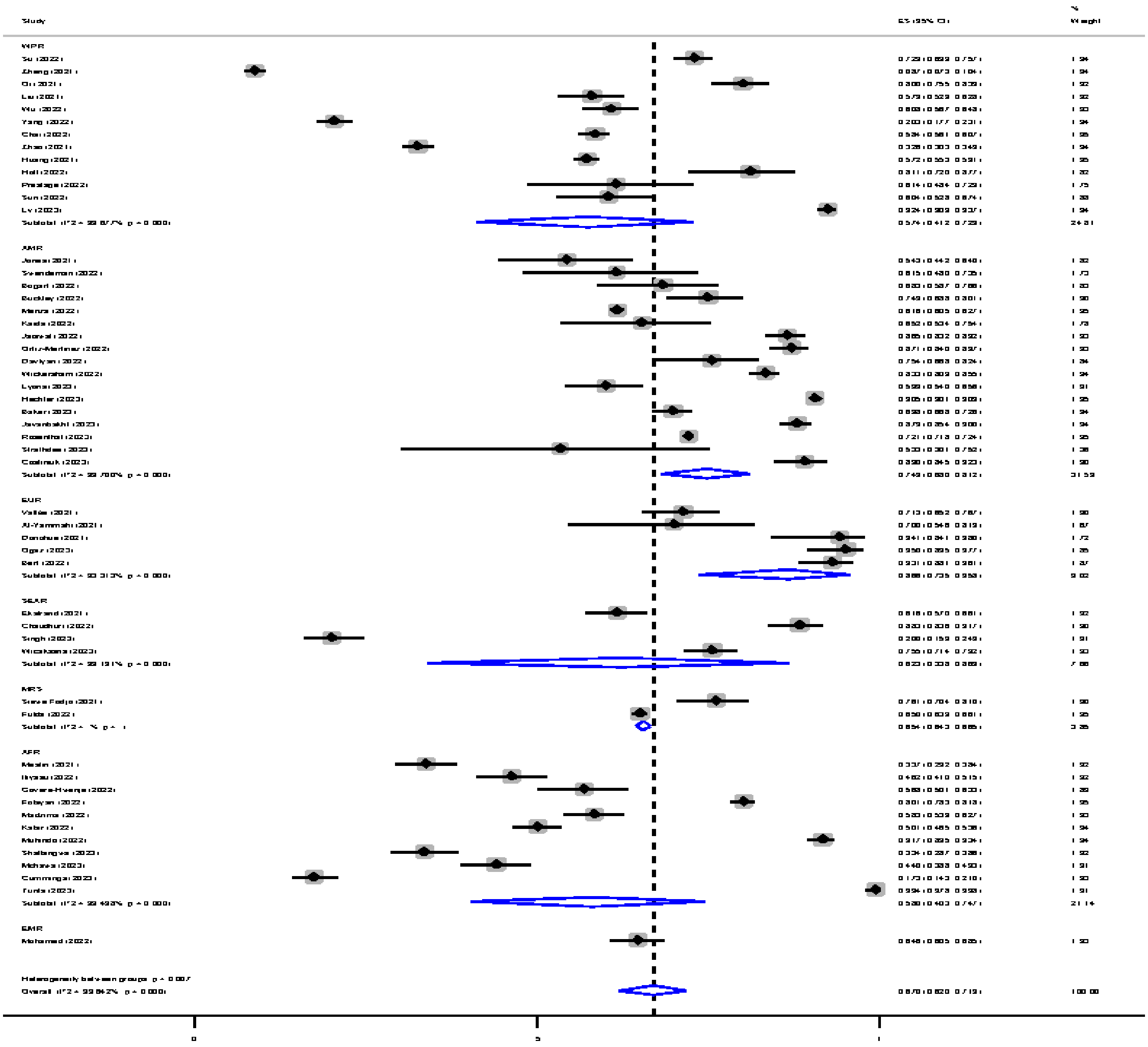
Study
ES (95% CI)
% Weight
WPR
AMR
EUR
SEAR
AFR
EMR

Supplementary Figure 6: Forest plots of the results of random-effects model meta-analysis of the prevalence (%) of COVID-19 vaccine acceptance according to the WHO region (n = 53 studies, N = 166,455 participants) pooled using inverse variance weights. Each black-colored solid square represents the effect size of each characteristic, while the ends of the adjoining horizontal lines represent lower (left) and upper (right) confidence intervals. The blue-colored hollow diamond at the bottom denotes the overall estimated effect size and the 95% CI. All statistics were based on a two-sided t-test. ES, Effect Size.

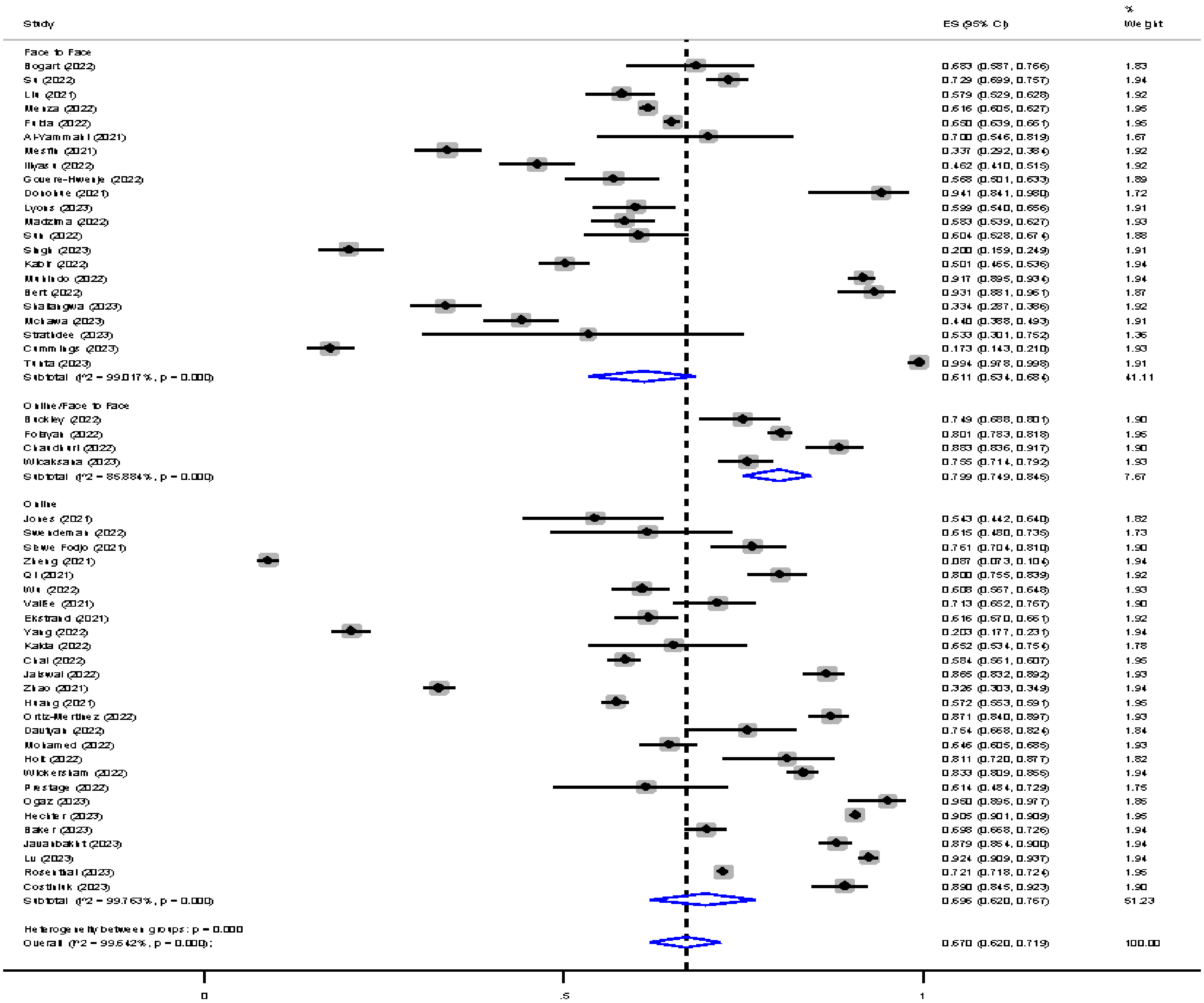

COVID-19 Vaccine Acceptance Among PLHIV (By Data Collection Method)

Supplementary Figure 7: Forest plots of the results of random-effects model meta-analysis of the prevalence (%) of COVID-19 vaccine acceptance according to the data collection method employed (n = 53 studies, N = 166,455 participants) pooled using inverse variance weights. Each black-colored solid square represents the effect size of each characteristic, while the ends of the adjoining horizontal lines represent lower (left) and upper (right) confidence intervals. The blue-colored hollow diamond at the bottom denotes the overall estimated effect size and the 95% CI. All statistics were based on a two-sided t-test. ES, Effect Size.

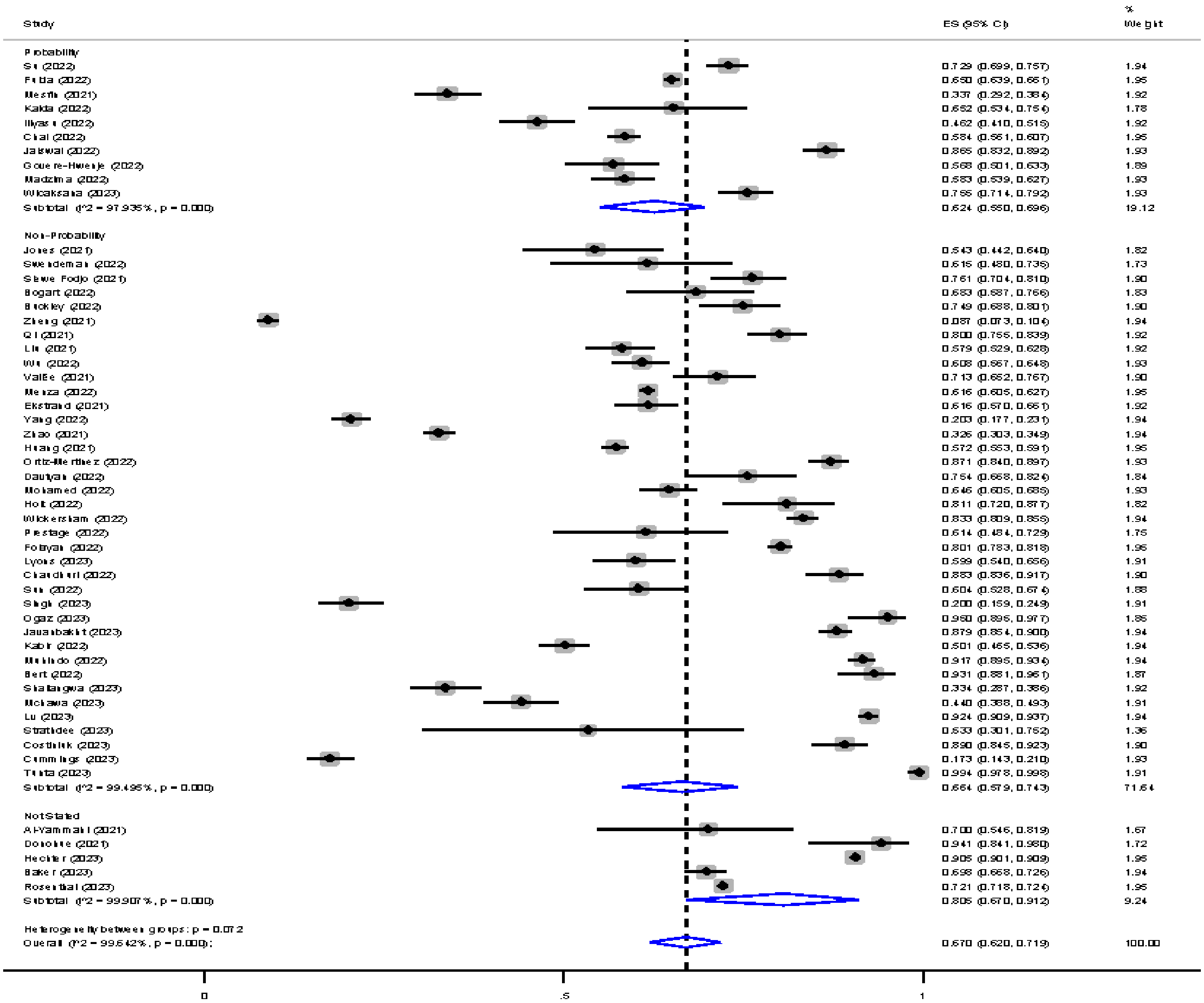

COVID-19 Vaccine Acceptance Among PLHIV (By Sampling Method)

Supplementary Figure 8: Forest plots of the results of random-effects model meta-analysis of the prevalence (%) of COVID-19 vaccine acceptance according to the sampling method employed (n = 53 studies, N = 166,455 participants) pooled using inverse variance weights. Each black-colored solid square represents the effect size of each characteristic, while the ends of the adjoining horizontal lines represent lower (left) and upper (right) confidence intervals. The blue-colored hollow diamond at the bottom denotes the overall estimated effect size and the 95% CI. All statistics were based on a two-sided t-test. ES, Effect Size.

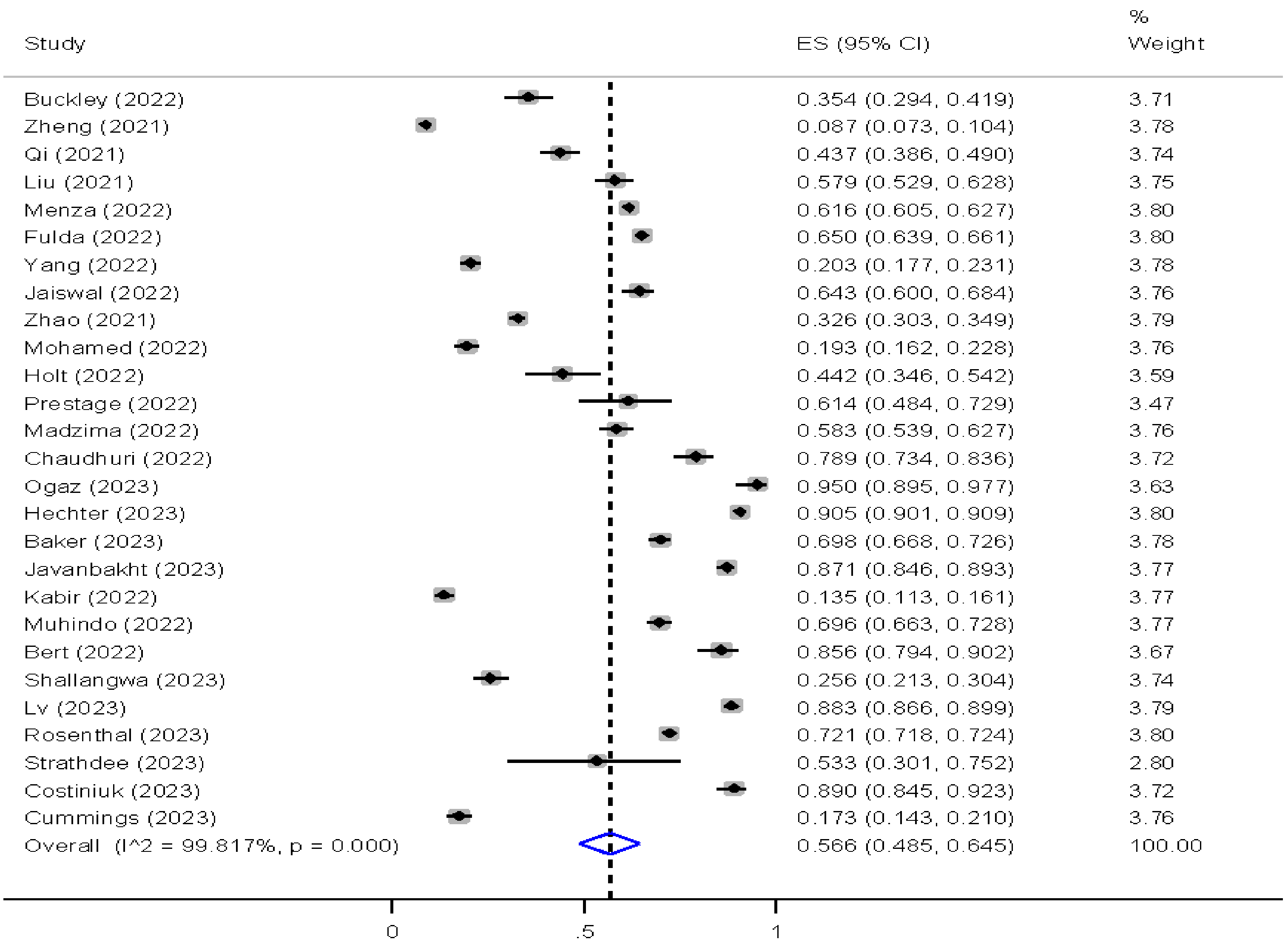

COVID-19 Vaccine Uptake Among PLHIV (Globally)

Supplementary Figure 9: Forest plots of the results of random-effects model meta-analysis of the prevalence (%) of COVID-19 vaccine uptake among PLHIV globally (n = 27 studies, N = 150,926 participants) pooled using inverse variance weights. Each black-colored solid square represents the effect size of each characteristic, while the ends of the adjoining horizontal lines represent lower (left) and upper (right) confidence intervals. The blue-colored hollow diamond at the bottom denotes the overall estimated effect size and the 95% CI. All statistics were based on a two-sided t-test. ES, Effect Size.

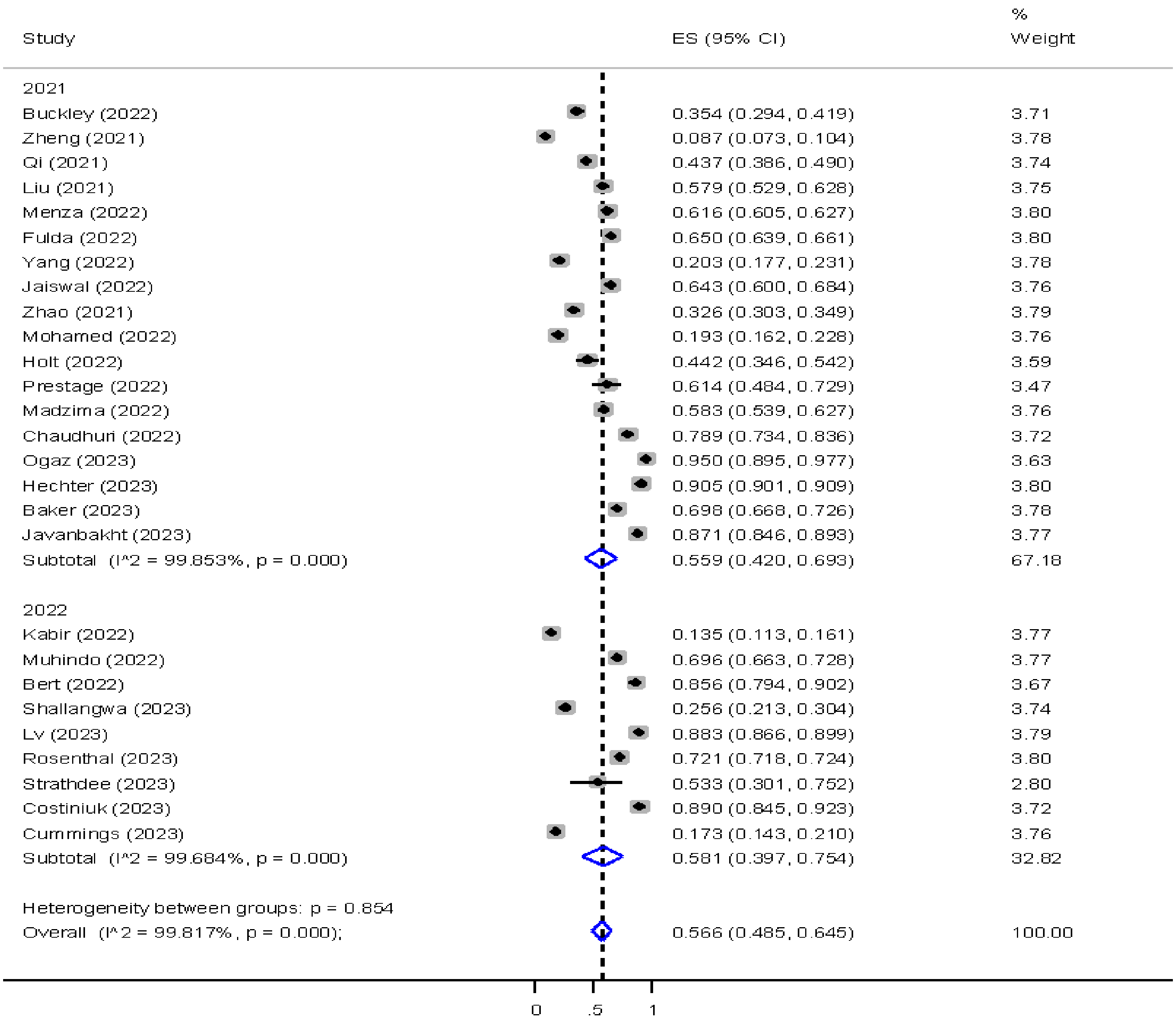

COVID-19 Vaccine Uptake Among PLHIV (By Study Year)

Supplementary Figure 10: Forest plots of the results of random-effects model meta-analysis of the prevalence (%) of COVID-19 vaccine uptake among PLHIV according to the study year (n = 27 studies, N = 150,926 participants) pooled using inverse variance weights. Each black-colored solid square represents the effect size of each characteristic, while the ends of the adjoining horizontal lines represent lower (left) and upper (right) confidence intervals. The blue-colored hollow diamond at the bottom denotes the overall estimated effect size and the 95% CI. All statistics were based on a two-sided t-test. ES, Effect Size.

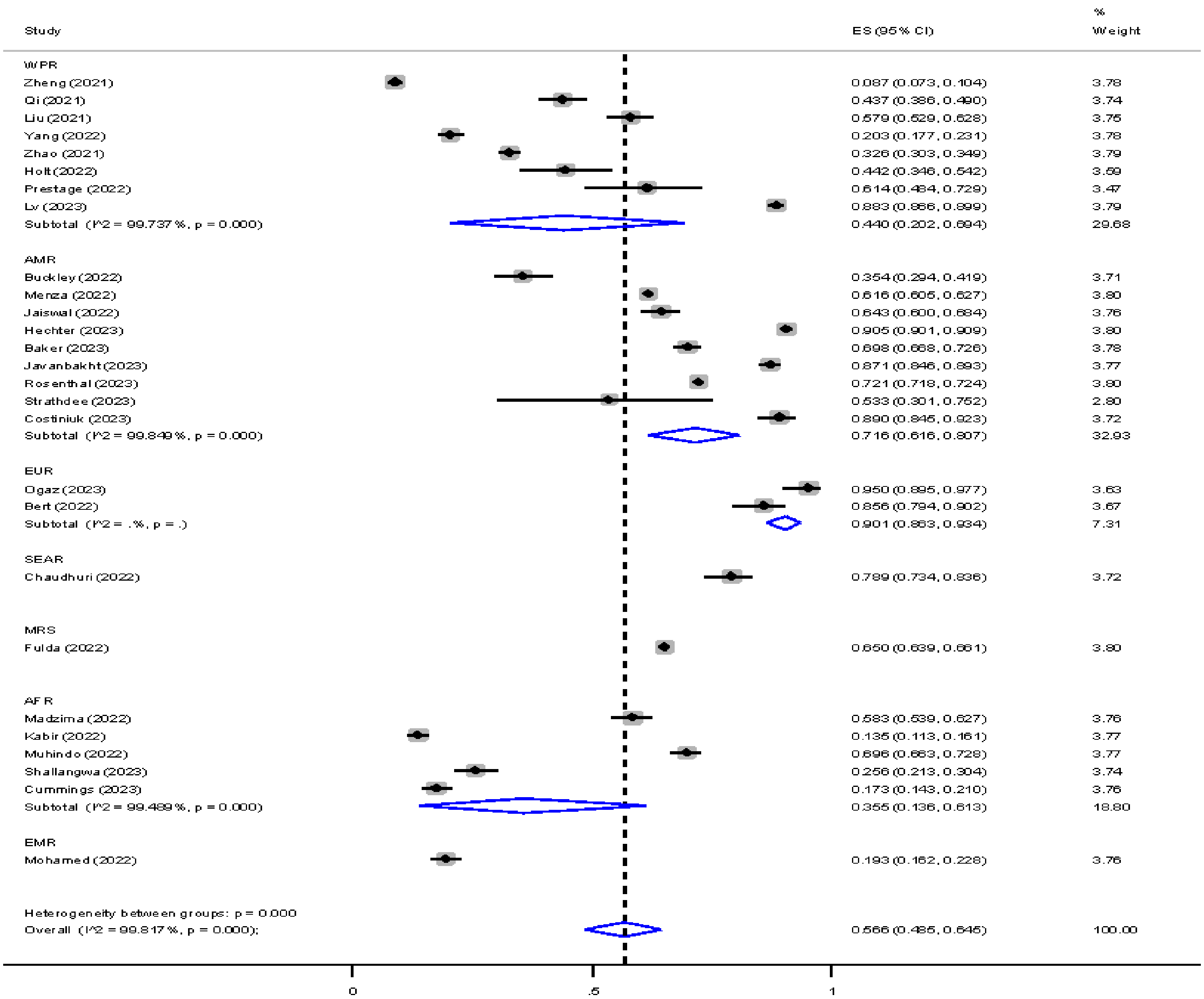

COVID-19 Vaccine uptake Among PLHIV (By WHO Region)

Supplementary Figure 11: Forest plots of the results of random-effects model meta-analysis of the prevalence (%) of COVID-19 vaccine uptake among PLHIV according to the WHO region (n = 27 studies, N = 150,926 participants) pooled using inverse variance weights. Each black-colored solid square represents the effect size of each characteristic, while the ends of the adjoining horizontal lines represent lower (left) and upper (right) confidence intervals. The blue-colored hollow diamond at the bottom denotes the overall estimated effect size and the 95% CI. All statistics were based on a two-sided t-test. ES, Effect Size.

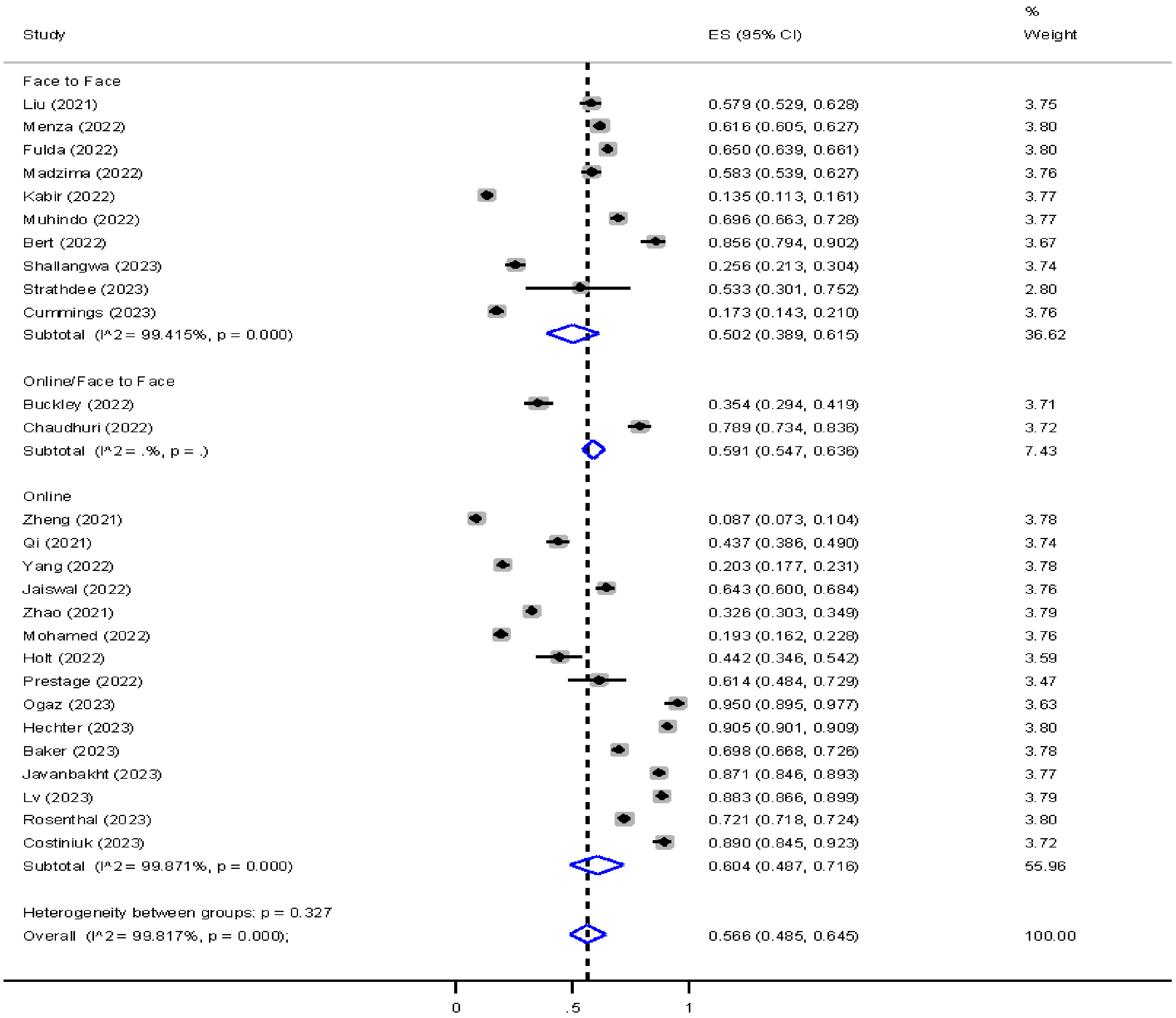

COVID-19 Vaccine uptake Among PLHIV (By Data Collection Method)

Supplementary Figure 12: Forest plots of the results of random-effects model meta-analysis of the prevalence (%) of COVID-19 vaccine uptake among PLHIV according to the data collection method (n = 27 studies, N = 150,926 participants) pooled using inverse variance weights. Each black-colored solid square represents the effect size of each characteristic, while the ends of the adjoining horizontal lines represent lower (left) and upper (right) confidence intervals. The blue-colored hollow diamond at the bottom denotes the overall estimated effect size and the 95% CI. All statistics were based on a two-sided t-test. ES, Effect Size.

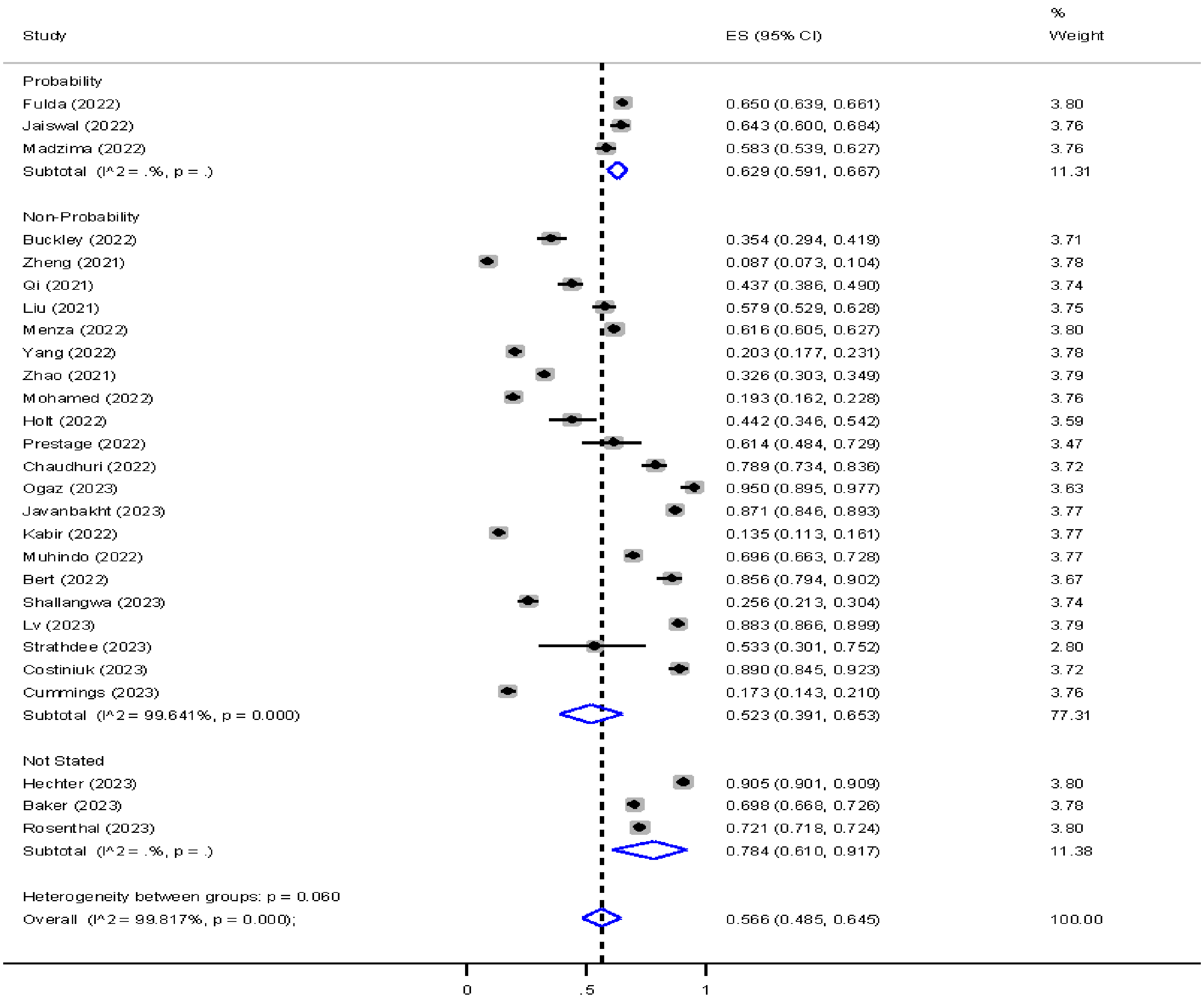

COVID-19 Vaccine Uptake Among PLHIV (By Sampling Method)

Supplementary Figure 13: Forest plots of the results of random-effects model meta-analysis of the prevalence (%) of COVID-19 vaccine uptake among PLHIV according to the sampling method employed (n = 27 studies, N = 150,926 participants) pooled using inverse variance weights. Each black-colored solid square represents the effect size of each characteristic, while the ends of the adjoining horizontal lines represent lower (left) and upper (right) confidence intervals. The blue-colored hollow diamond at the bottom denotes the overall estimated effect size and the 95% CI. All statistics were based on a two-sided t-test. ES, Effect Size.

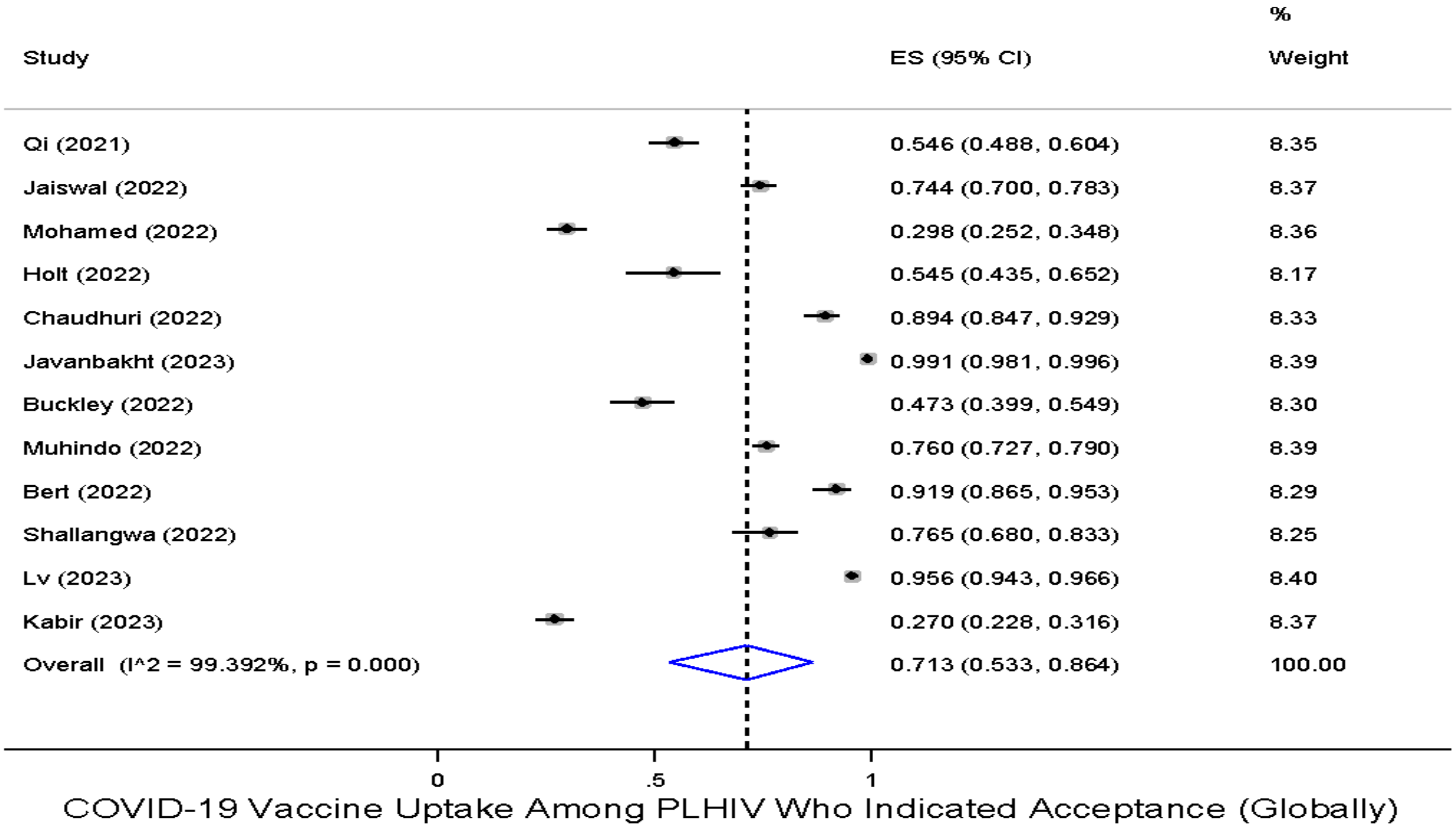

Supplementary Figure 14: Forest plots of the results of random-effects model meta-analysis of the prevalence (%) of COVID-19 vaccine uptake among PLHIV who indicated acceptance globally (n = 13 studies, N = 6,186 participants) pooled using inverse variance weights. Each black-colored solid square represents the effect size of each characteristic, while the ends of the adjoining horizontal lines represent lower (left) and upper (right) confidence intervals. The blue-colored hollow diamond at the bottom denotes the overall estimated effect size and the 95% CI. All statistics were based on a two-sided t-test. ES, Effect Size.

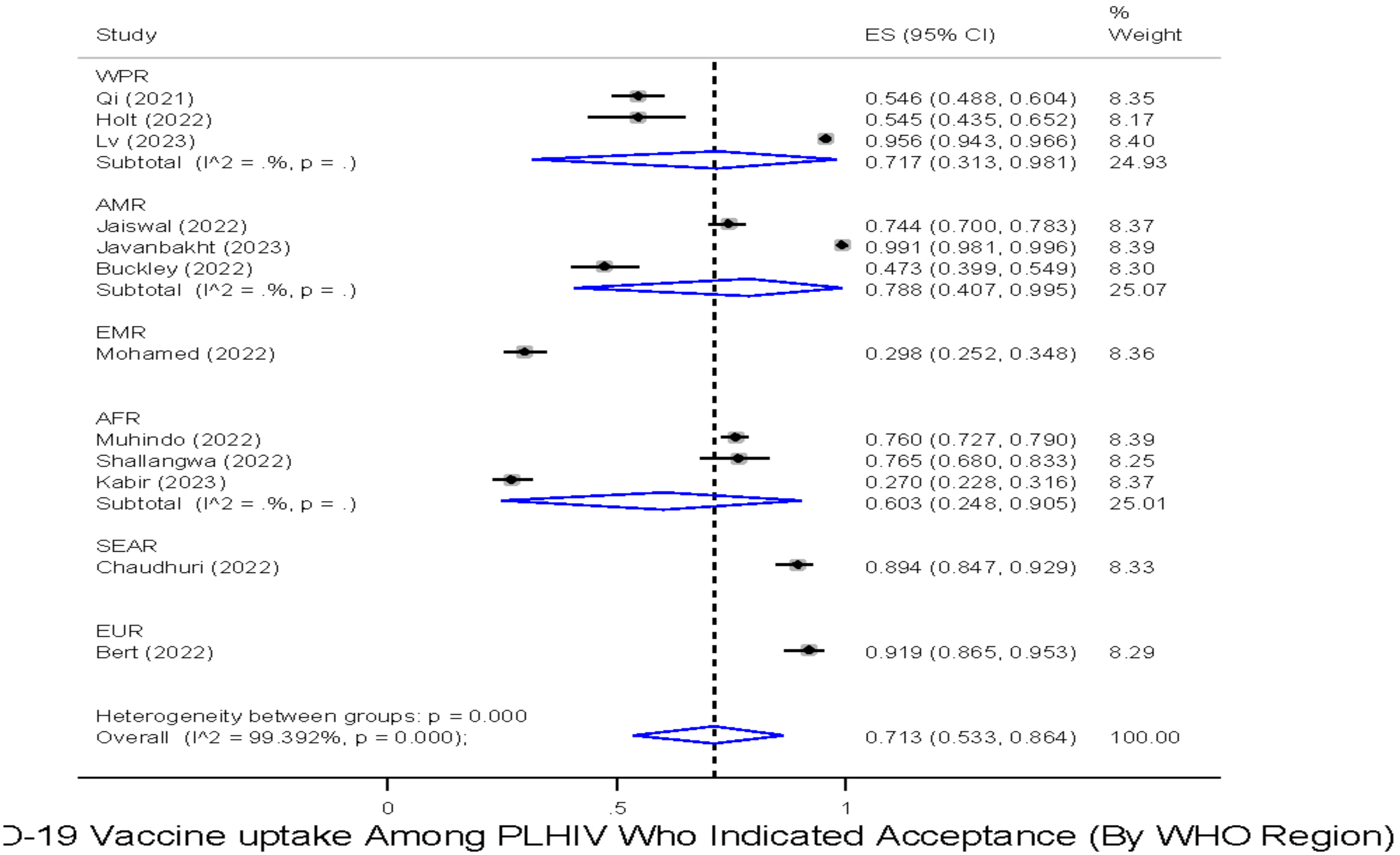

Supplementary Figure 15: Forest plots of the results of random-effects model meta-analysis of the prevalence (%) of COVID-19 vaccine uptake among PLHIV who indicated acceptance according to the WHO region (n = 13 studies, N = 6,186 participants) pooled using inverse variance weights. Each black-colored solid square represents the effect size of each

characteristic, while the ends of the adjoining horizontal lines represent lower (left) and upper (right) confidence intervals. The blue-colored hollow diamond at the bottom denotes the overall estimated effect size and the 95% CI. All statistics were based on a two-sided t-test. ES, Effect Size.

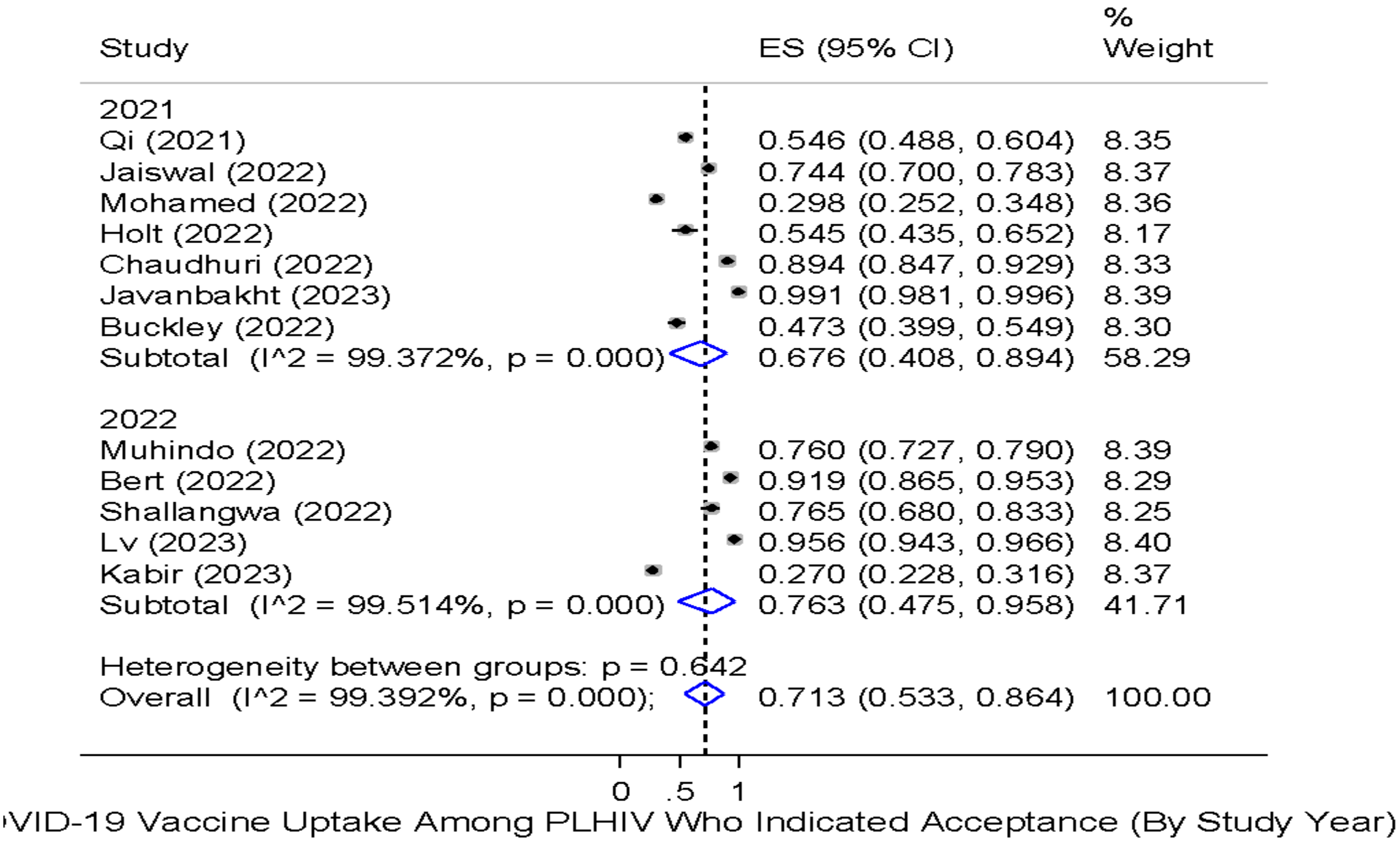

Supplementary Figure 16: Forest plots of the results of random-effects model meta-analysis of the prevalence (%) of COVID-19 vaccine uptake among PLHIV who indicated acceptance according to the study year (n = 13 studies, N = 6,186 participants) pooled using inverse variance weights. Each black-colored solid square represents the effect size of each characteristic, while

the ends of the adjoining horizontal lines represent lower (left) and upper (right) confidence intervals. The blue-colored hollow diamond at the bottom denotes the overall estimated effect size and the 95% CI. All statistics were based on a two-sided t-test. ES, Effect Size.

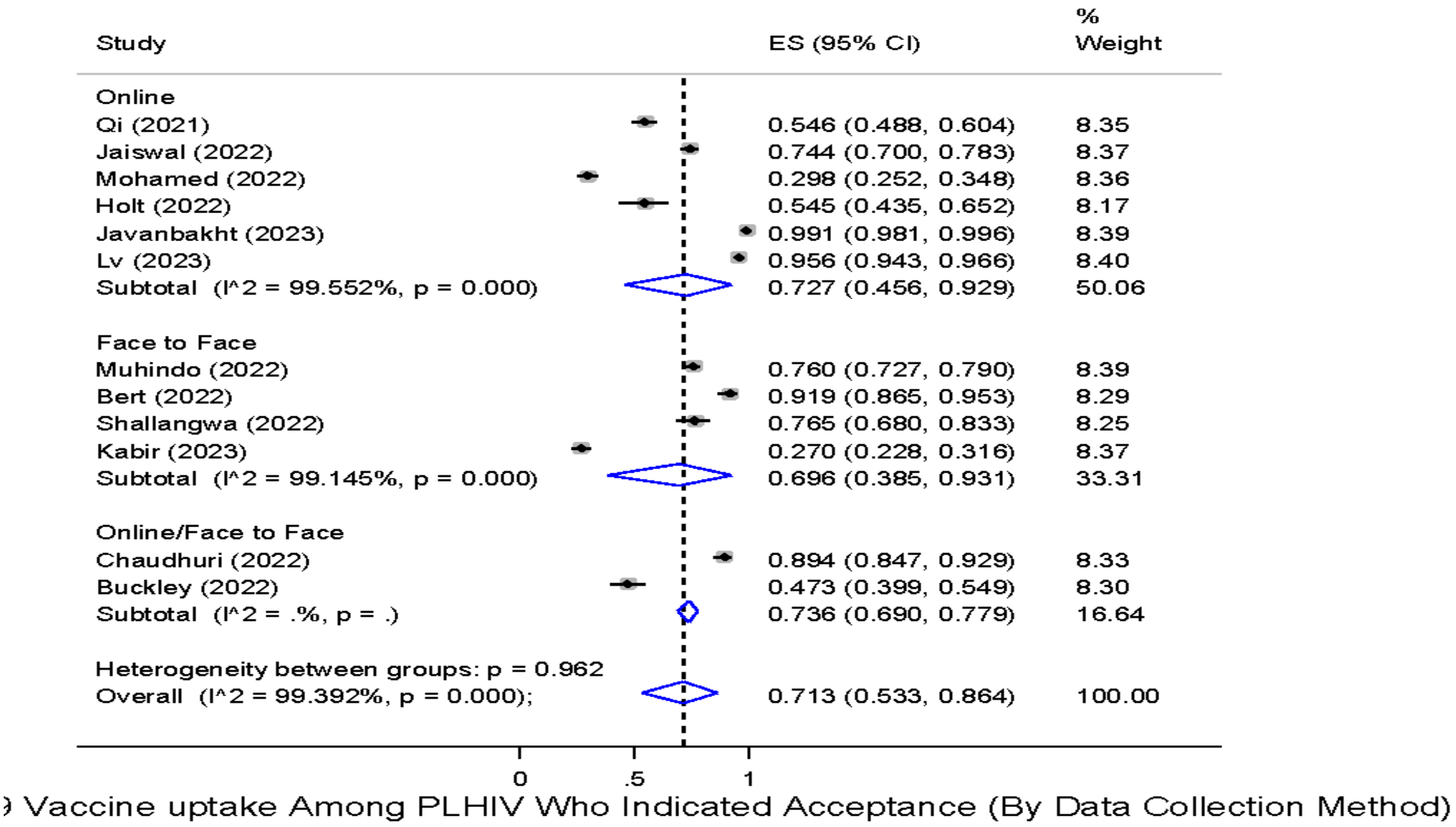

Supplementary Figure 17: Forest plots of the results of random-effects model meta-analysis of the prevalence (%) of COVID-19 vaccine uptake among PLHIV who indicated acceptance according to the data collection method employed (n = 13 studies, N = 6,186 participants) pooled using inverse variance weights. Each black-colored solid square represents the effect size of each characteristic, while the ends of the adjoining horizontal lines represent lower (left) and upper (right) confidence intervals. The blue-colored hollow diamond at the bottom denotes the overall estimated effect size and the 95% CI. All statistics were based on a two-sided t-test. ES, Effect Size.

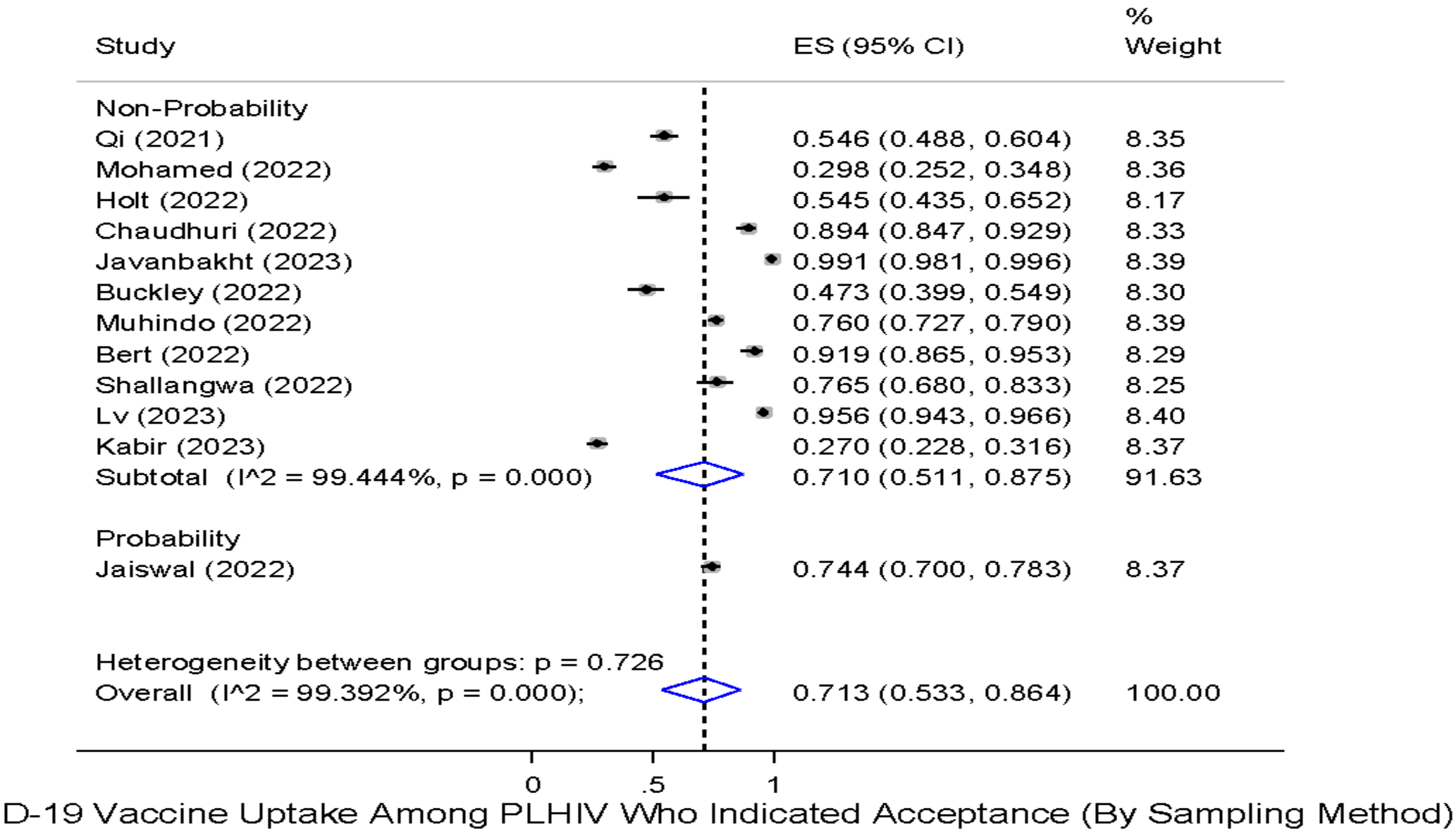

Supplementary Figure 18: Forest plots of the results of random-effects model meta-analysis of the prevalence (%) of COVID-19 vaccine uptake among PLHIV who indicated acceptance according to the sampling method employed (n = 13 studies, N = 6,186 participants) pooled using inverse variance weights. Each black-colored solid square represents the effect size of each

characteristic, while the ends of the adjoining horizontal lines represent lower (left) and upper (right) confidence intervals. The blue-colored hollow diamond at the bottom denotes the overall estimated effect size and the 95% CI. All statistics were based on a two-sided t-test. ES, Effect Size.

SECTION C

Results of the meta-analysis of COVID-19 vaccine acceptance and uptake using peer-reviewed studies only

Fig a: Begg's funnel plot of included studies

Figure b: Egger graph of included studies (P = 0.102)

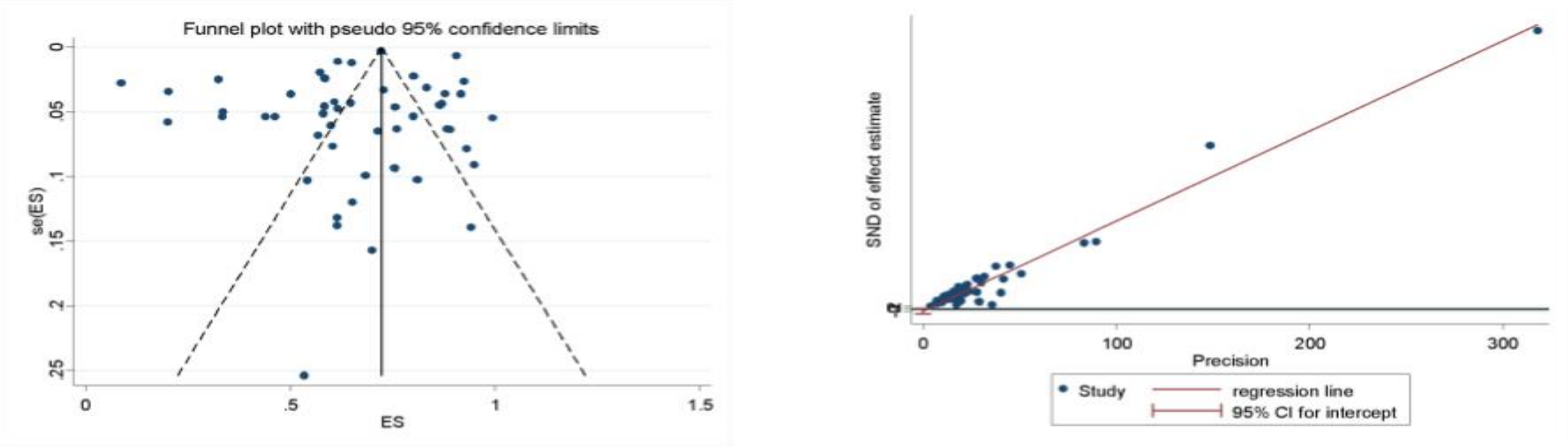

Supplementary Figure 19: Publication bias assessment of studies reporting COVID-19 vaccine acceptance rate

Figure c: Begg's funnel plot of included studies

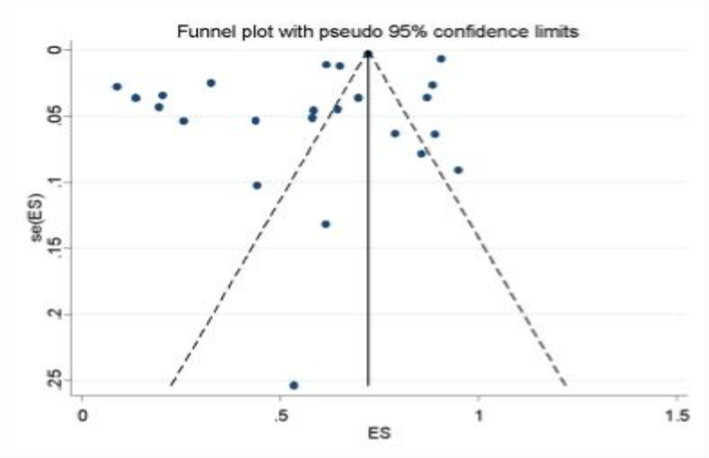

Figure d: Egger graph of included studies (P = 0.059)

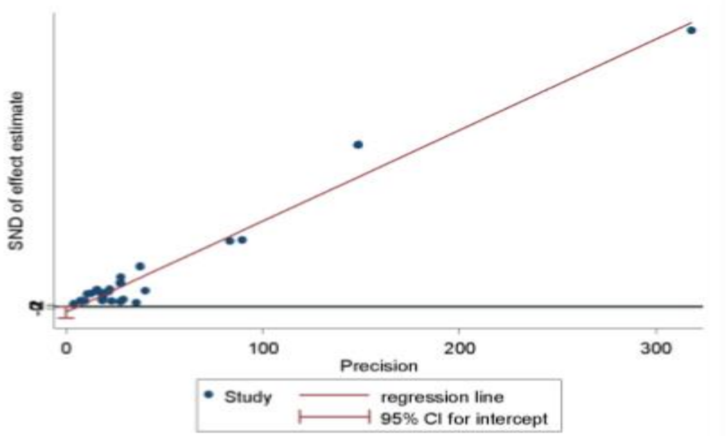

Supplementary Figure 20: Publication bias assessment of studies reporting COVID-19 vaccine uptake rate

Figure e: Begg's funnel plot of included studies

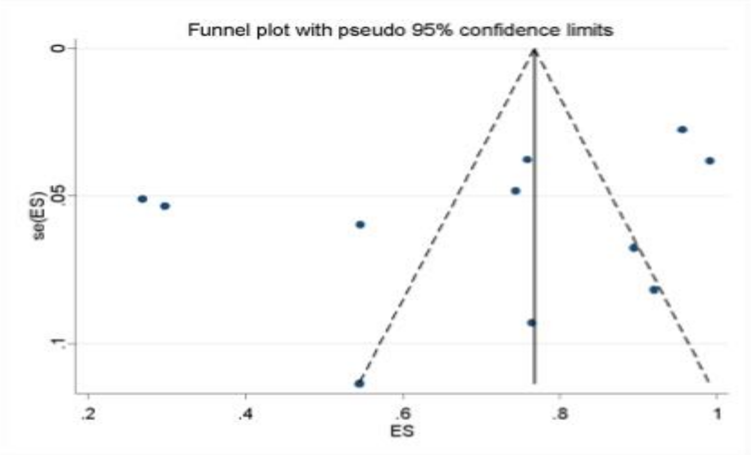

Figure f: Egger graph of included studies (P = 0.203)

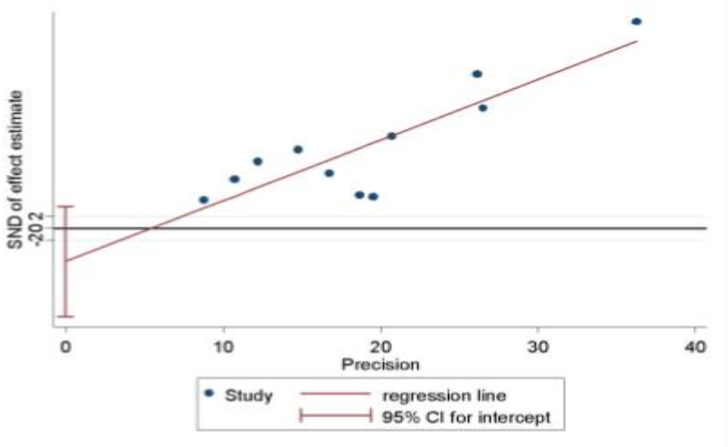

Supplementary Figure 21: Assessment of publication bias among studies reporting each of the three outcomes. All the statistical tests were two-sided. ES, Effect Size. a, Begg's funnel plot of included studies reporting acceptance rate (n = 50 studies). **b,** Egger graph of included studies reporting acceptance rate (n = 50 studies). **c,** Begg's funnel plot of included studies reporting uptake rate (n = 24 studies). **d,** Egger graph of included studies reporting uptake rate (n = 24 studies). **e,** Begg's funnel plot of studies reporting uptake rate in PLHIV who indicated acceptance (n = 12 studies). **f,** Egger graph of studies reporting uptake among accepting group (n = 12 studies).

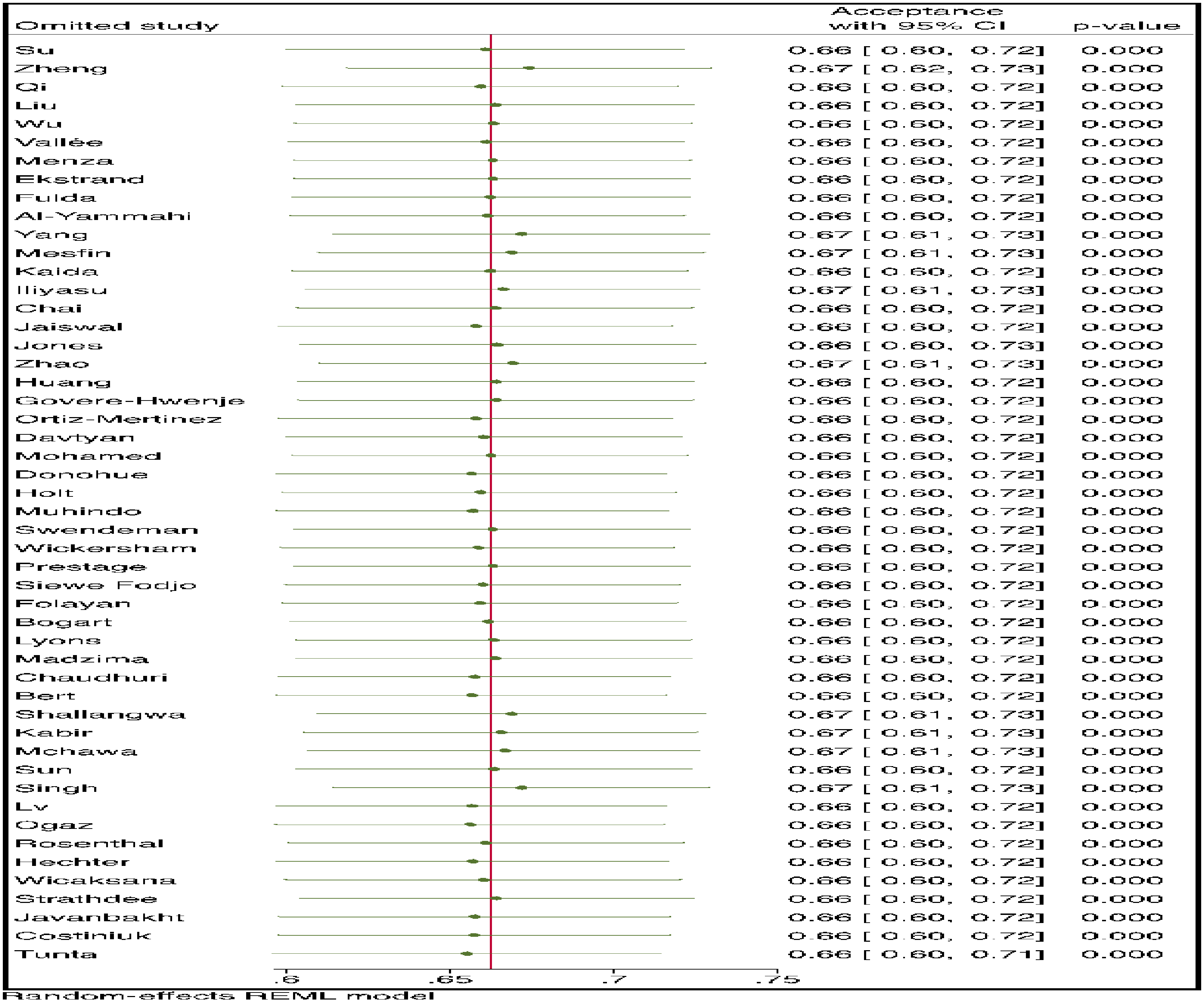

Random-effects REML model

Supplementary Figure 22: Forest plot of sensitivity analyses for each study reporting the acceptance rate of the COVID-19 vaccine among PLHIV using random effects model meta-analysis. Each solid square represents the effect size of each characteristic, while the ends of the adjoining horizontal lines represent lower (left) and upper (right) confidence intervals. (n = 50 studies).

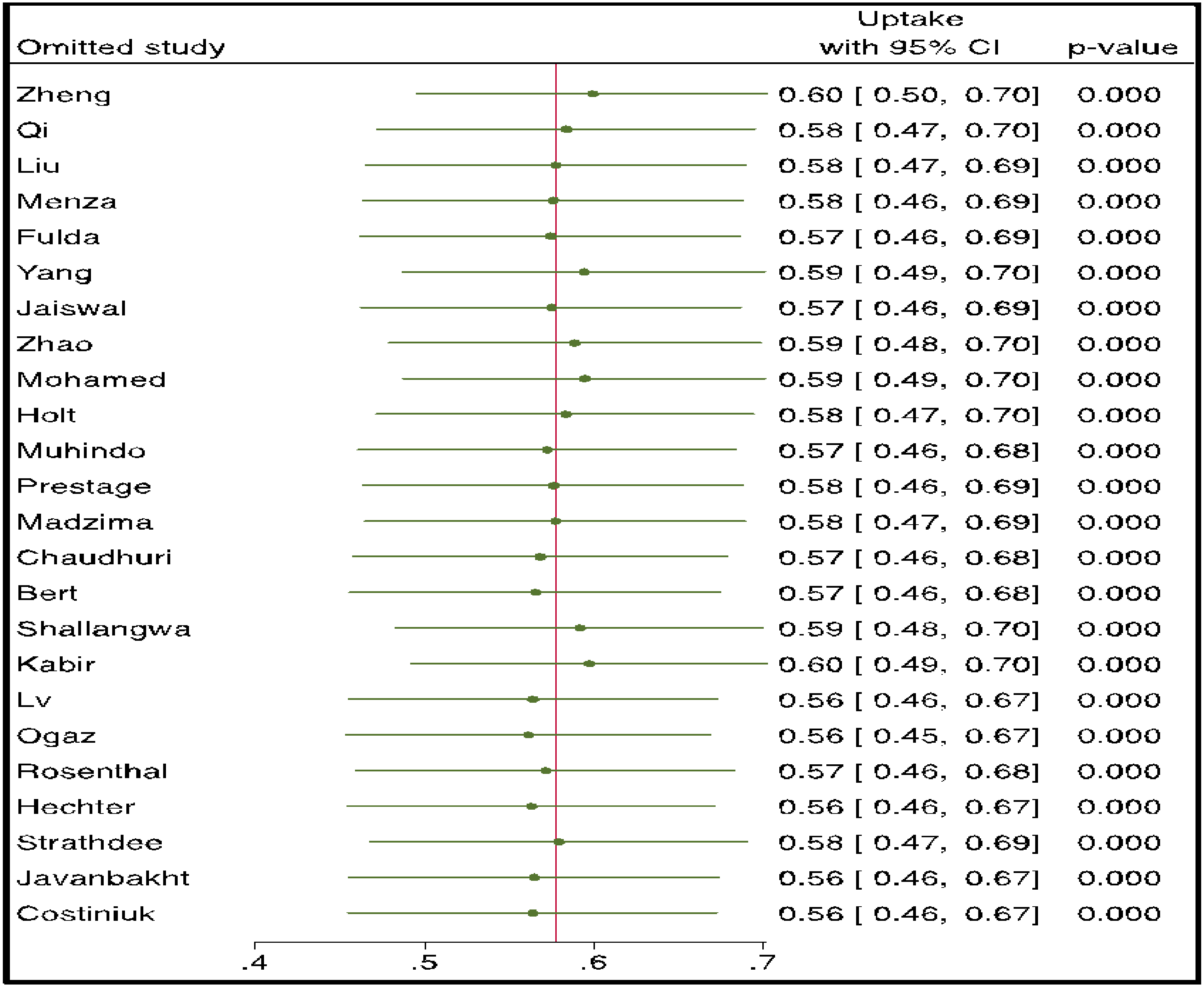

Random-effects REML model

7

Supplementary Figure 23: Forest plot of sensitivity analyses for each study reporting the uptake rate of the COVID-19 vaccine among PLHIV using random effects model meta-analysis. Each solid square represents the effect size of each characteristic, while the ends of the adjoining horizontal lines represent lower (left) and upper (right) confidence intervals. (n = 24 studies).

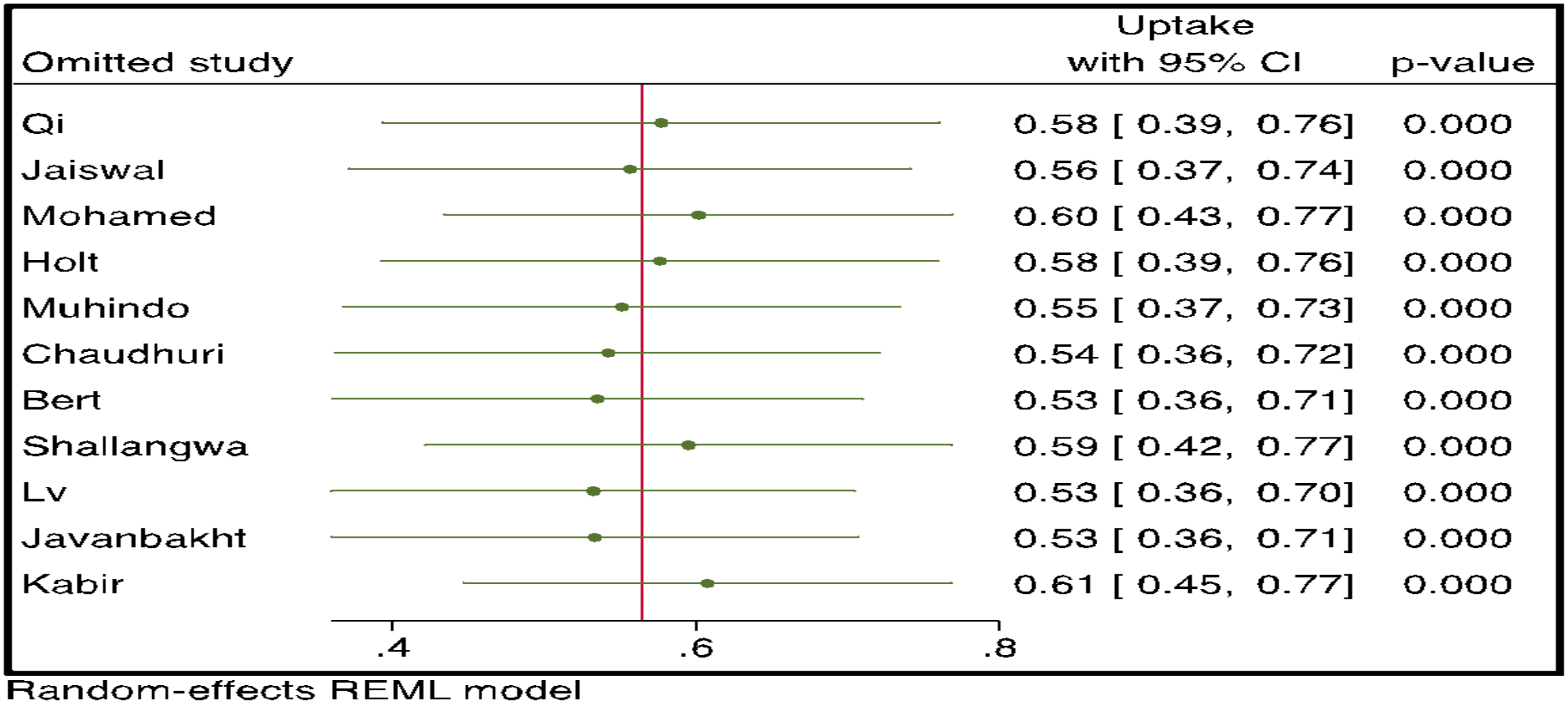

Supplementary Figure 24: Forest plot of sensitivity analyses for each study reporting the uptake rate of the COVID-19 vaccine among PLHIV who indicated acceptance using random effects model meta-analysis. Each solid square represents the effect size of each characteristic, while the ends of the adjoining horizontal lines represent lower (left) and upper (right) confidence intervals. (n = 12 studies).

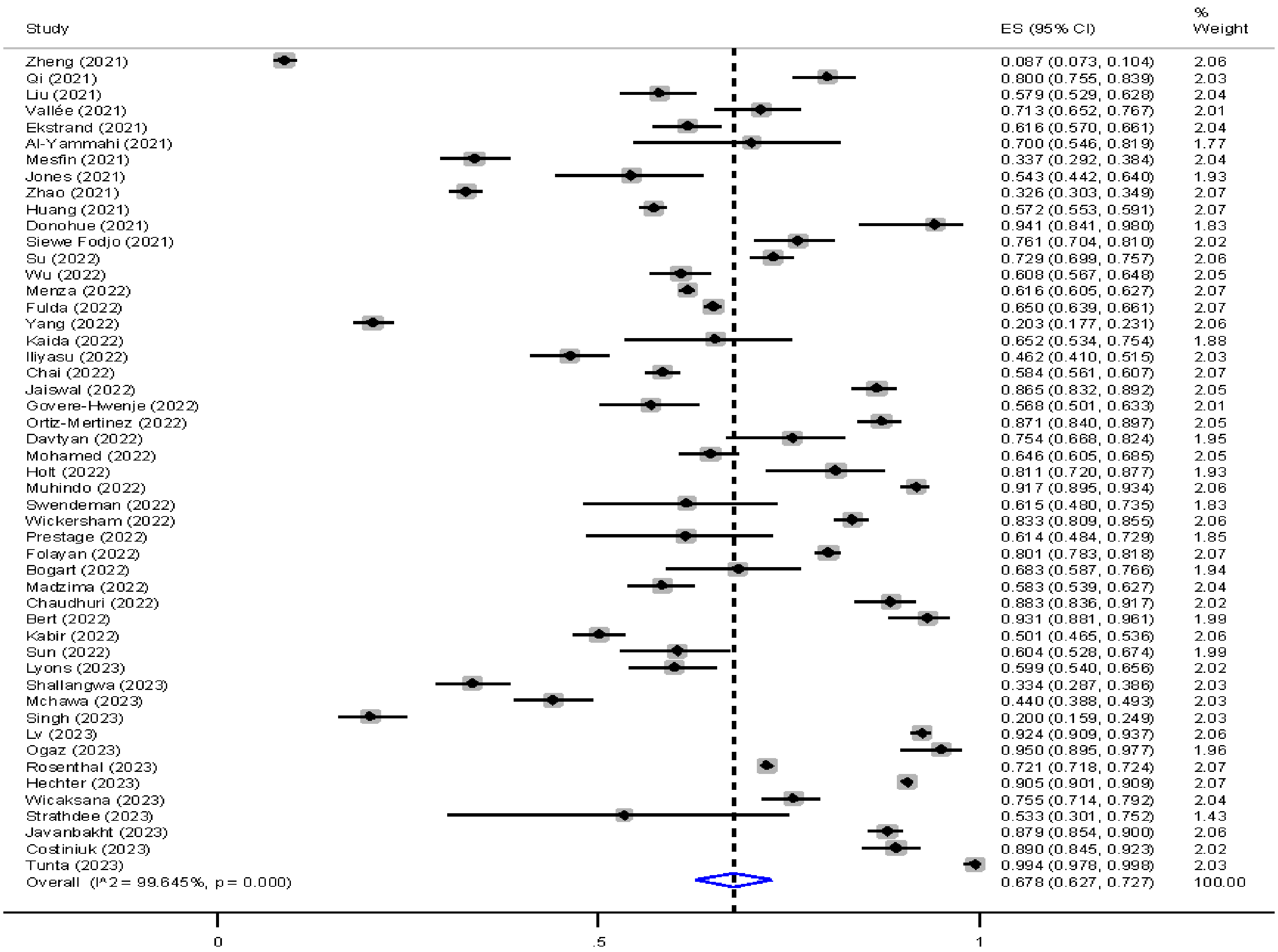

COVID-19 Vaccine Acceptance Among PLHIV (Globally)

Supplementary Figure 25: Forest plots of the results of random-effects model meta-analysis of the prevalence (%) of COVID-19 vaccine acceptance globally (n = 50 studies) pooled using inverse variance weights. Each black-colored solid square represents the effect size of each characteristic, while the ends of the adjoining horizontal lines represent lower (left) and upper (right) confidence intervals. The blue-colored hollow diamond at the bottom denotes the overall estimated effect size and the 95% CI. All statistics were based on a two-sided t-test. ES, Effect Size.

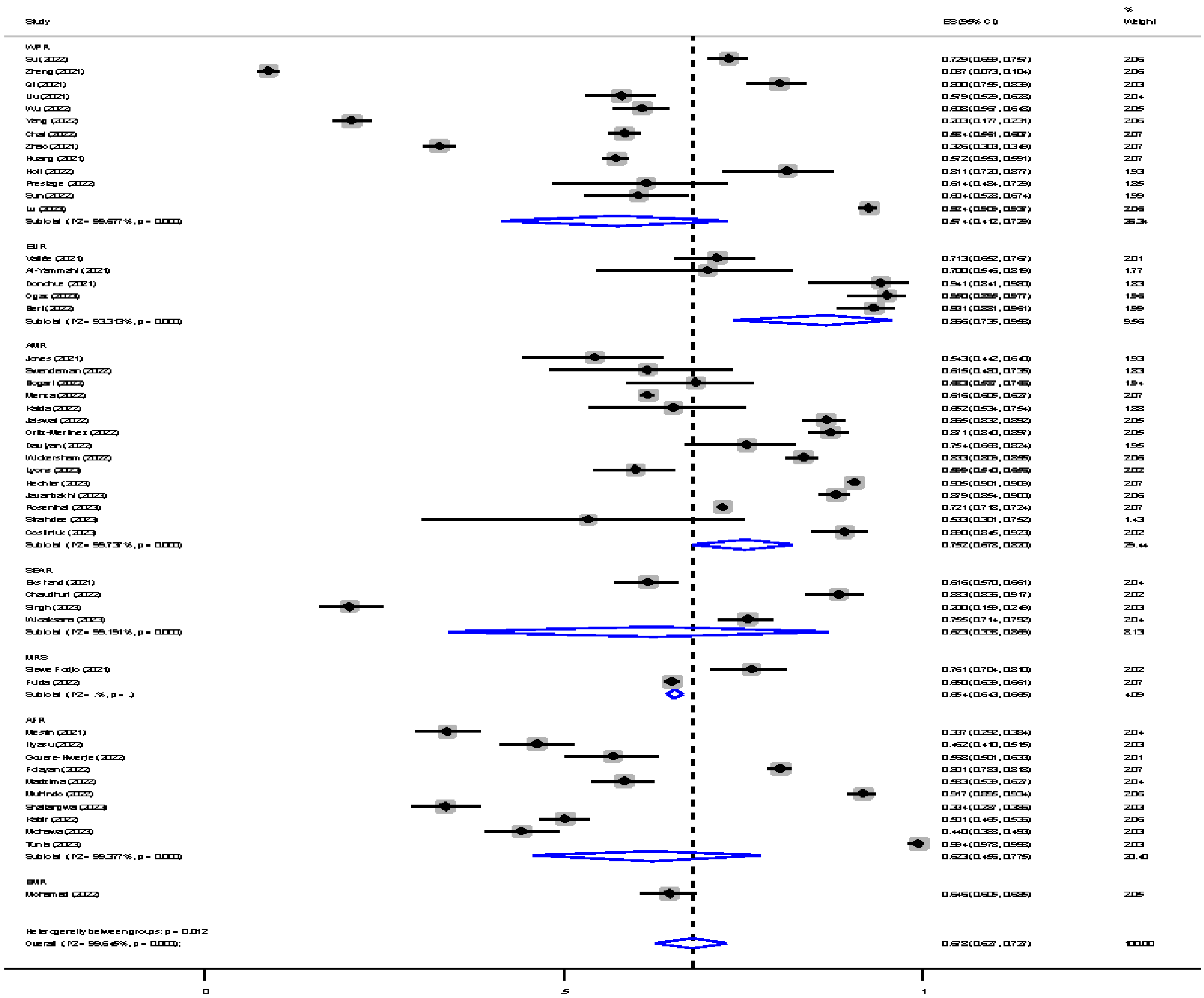

COVID-19 Vaccine Acceptance Among PLHIV (By WHO Region)

Supplementary Figure 26: Forest plots of the results of random-effects model meta-analysis of the prevalence (%) of COVID-19 vaccine acceptance according to the WHO region (n = 50 studies) pooled using inverse variance weights. Each black-colored solid square represents the effect size of each characteristic, while the ends of the adjoining horizontal lines represent lower (left) and upper (right) confidence intervals. The blue-colored hollow diamond at the bottom denotes the overall estimated effect size and the 95% CI. All statistics were based on a two-sided t-test. ES, Effect Size.

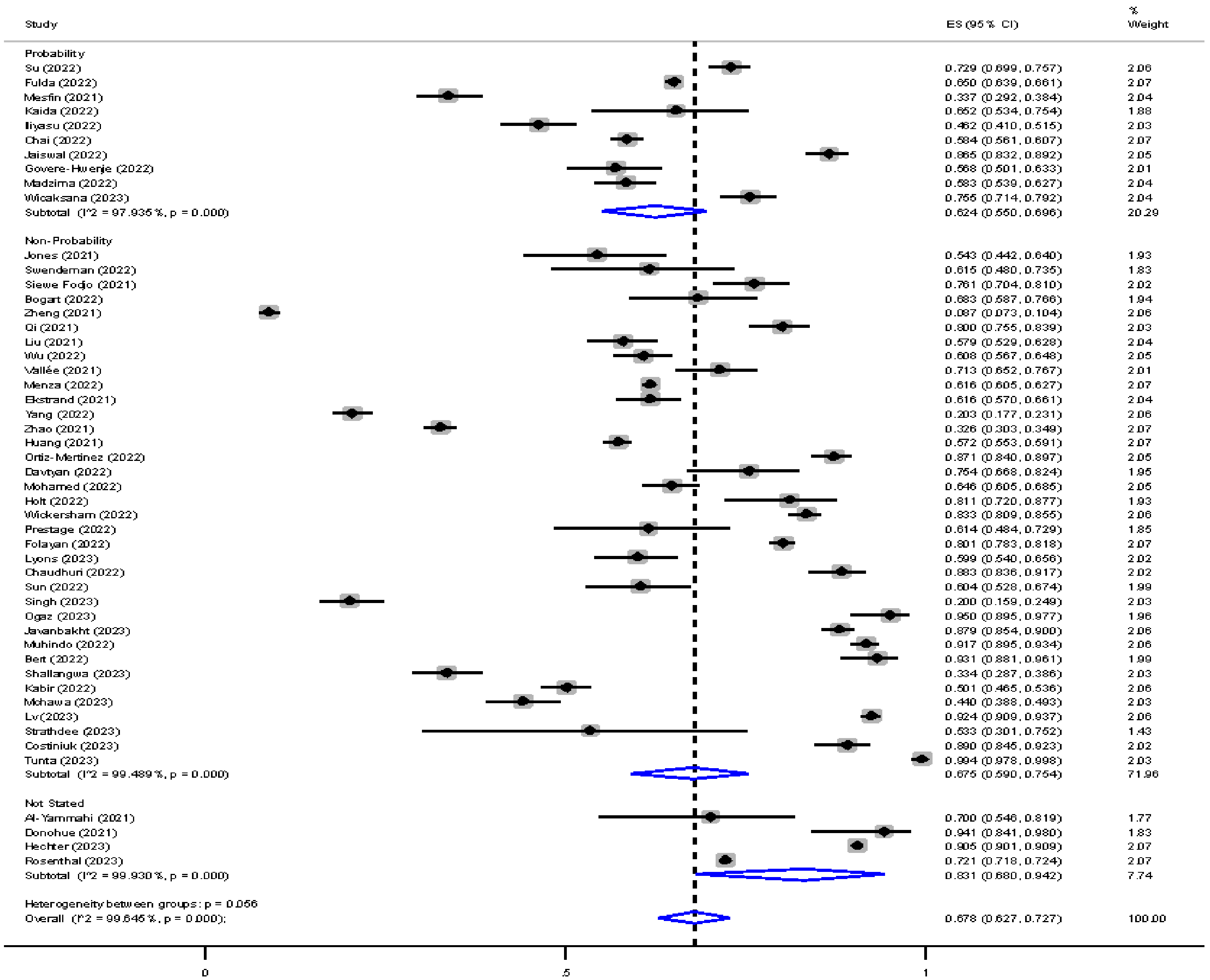

COVID-19 Vaccine Acceptance Among PLHIV (By Sampling Method)

Supplementary Figure 27: Forest plots of the results of random-effects model meta-analysis of the prevalence (%) of COVID-19 vaccine acceptance according to the sampling method employed (n = 50 studies) pooled using inverse variance weights. Each black-colored solid square represents the effect size of each characteristic, while the ends of the adjoining horizontal lines represent lower (left) and upper (right) confidence intervals. The blue-colored hollow diamond at the bottom denotes the overall estimated effect size and the 95% CI. All statistics were based on a two-sided t-test. ES, Effect Size.

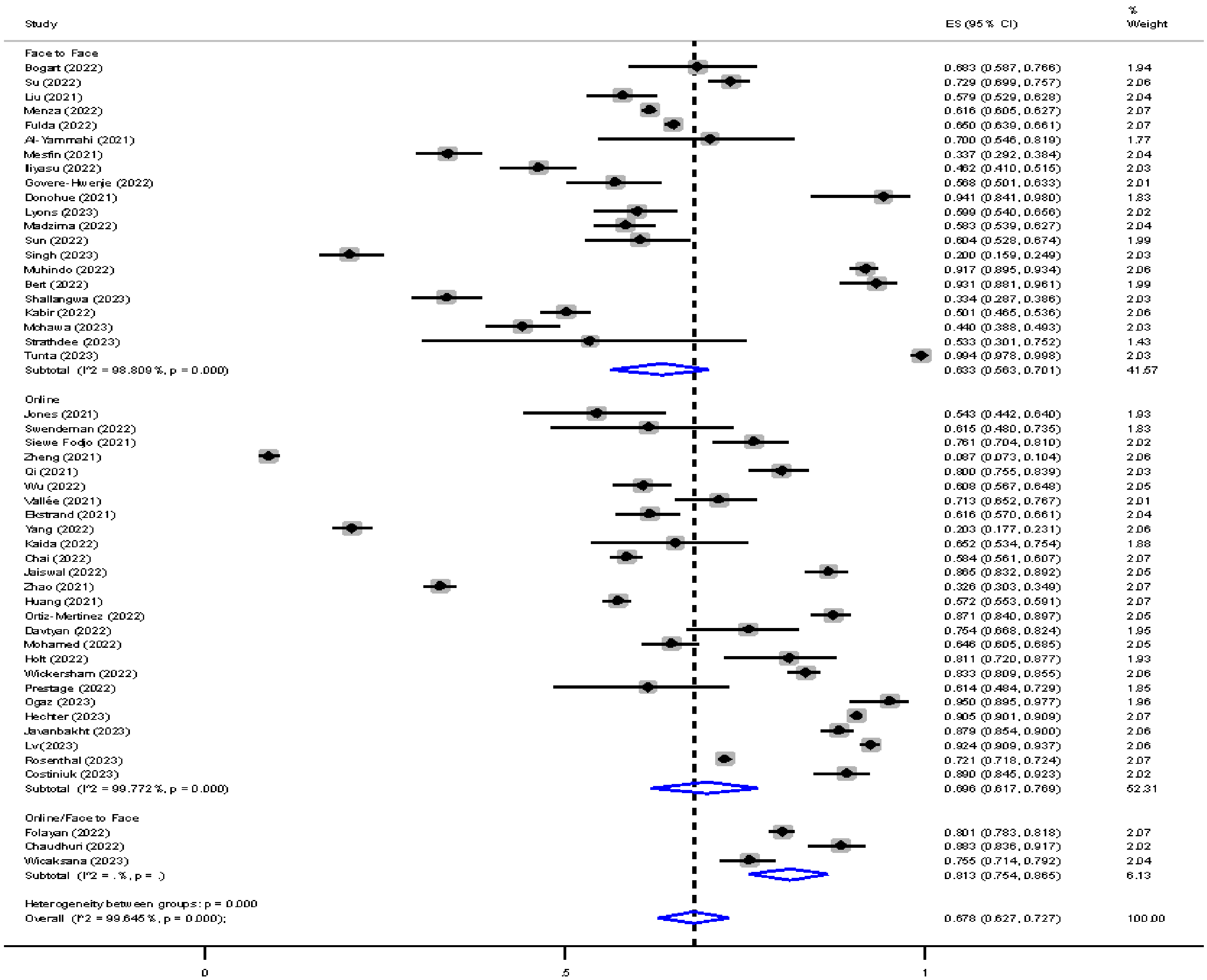

COVID-19 Vaccine Acceptance Among PLHIV (By Data Collection Method)[66]

Supplementary Figure 28: Forest plots of the results of random-effects model meta-analysis of the prevalence (%) of COVID-19 vaccine acceptance according to the data collection method (n = 50 studies) pooled using inverse variance weights. Each black-colored solid square represents the effect size of each characteristic, while the ends of the adjoining horizontal lines represent lower (left) and upper (right) confidence intervals. The blue-colored hollow diamond at the bottom denotes the overall estimated effect size and the 95% CI. All statistics were based on a two-sided t-test. ES, Effect Size.

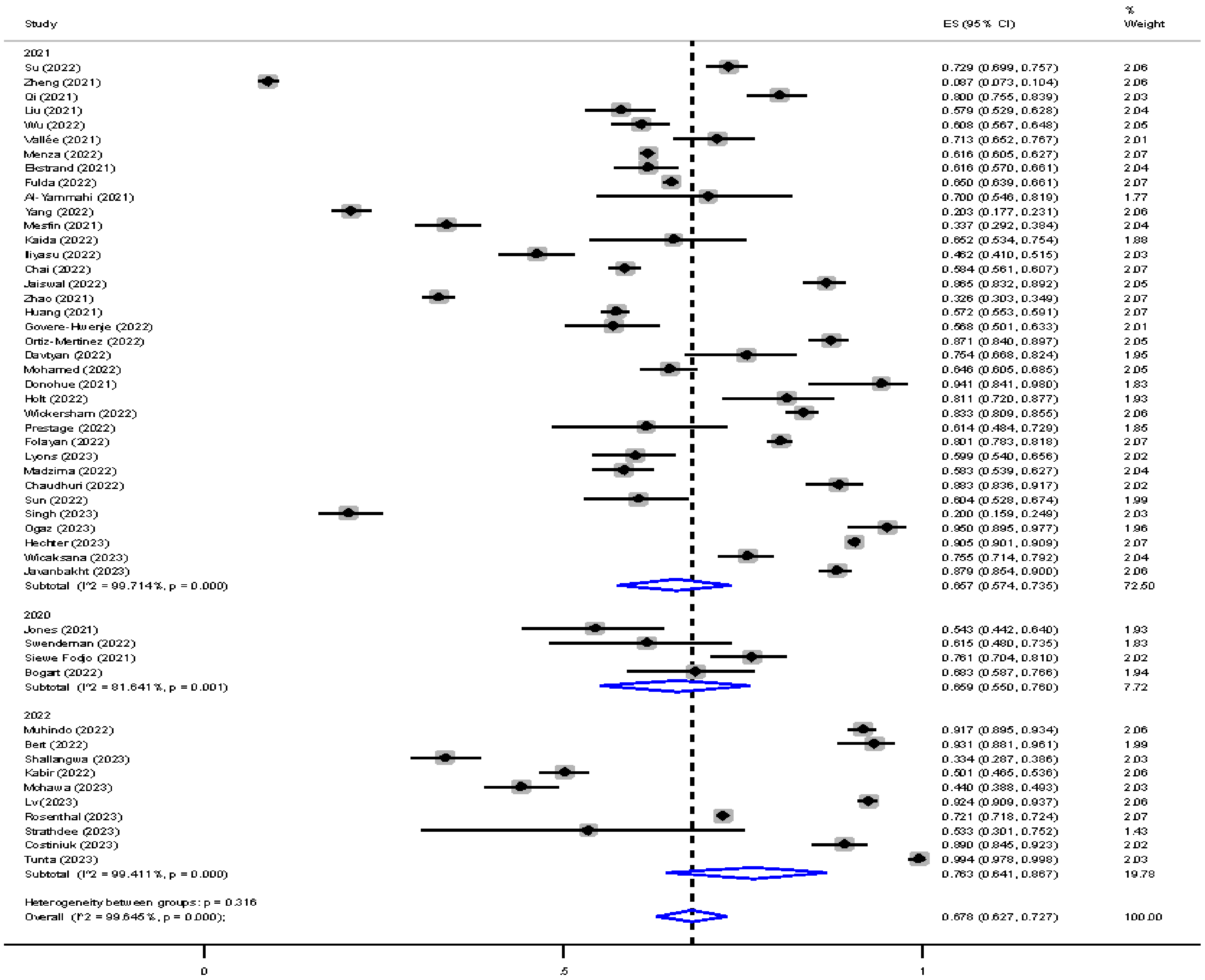

COVID-19 Vaccine Acceptance Among PLHIV (By Study Year)

Supplementary Figure 29: Forest plots of the results of random-effects model meta-analysis of the prevalence (%) of COVID-19 vaccine acceptance according to the study year (n = 50 studies) pooled using inverse variance weights. Each black-colored solid square represents the effect size of each characteristic, while the ends of the adjoining horizontal lines represent lower (left) and upper (right) confidence intervals. The blue-colored hollow diamond at the bottom denotes the overall estimated effect size and the 95% CI. All statistics were based on a two-sided t-test. ES, Effect Size.

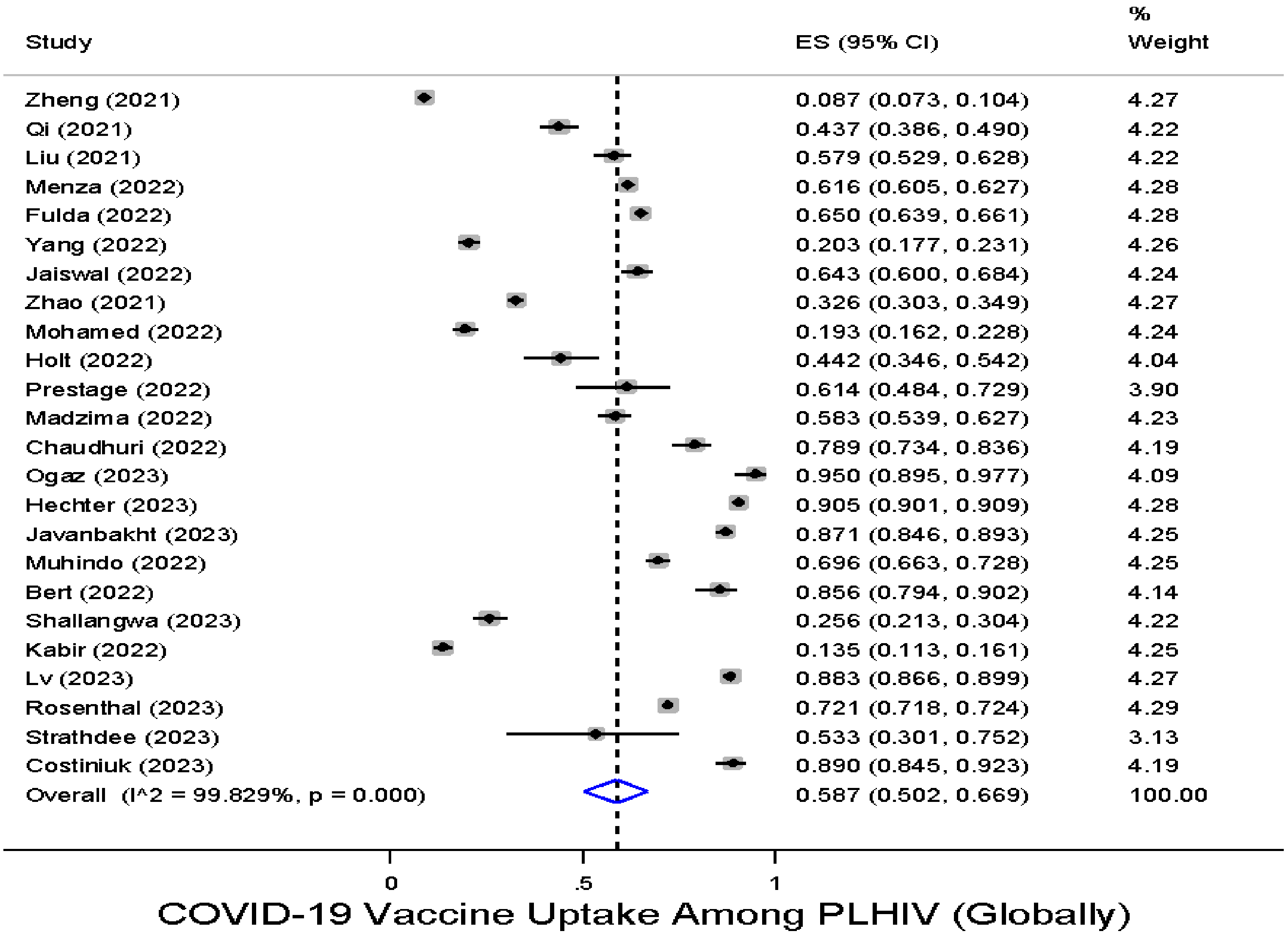
Study
ES (95% CI)
% Weight
Zheng (2021) 0.087 (0.073, 0.104) 4.27
Qi (2021) 0.437 (0.386, 0.490) 4.22
Liu (2021) 0.579 (0.529, 0.628) 4.22
Menza (2022) 0.616 (0.605, 0.627) 4.28
Fulda (2022) 0.650 (0.639, 0.661) 4.28
Yang (2022) 0.203 (0.177, 0.231) 4.26
Jaiswal (2022) 0.643 (0.600, 0.684) 4.24
Zhao (2021) 0.326 (0.303, 0.349) 4.27
Mohamed (2022) 0.193 (0.162, 0.228) 4.24
Holt (2022) 0.442 (0.346, 0.542) 4.04
Prestage (2022) 0.614 (0.484, 0.729) 3.90
Madzima (2022) 0.583 (0.539, 0.627) 4.23
Chaudhuri (2022) 0.789 (0.734, 0.836) 4.19
Ogaz (2023) 0.950 (0.895, 0.977) 4.09
Hechter (2023) 0.905 (0.901, 0.909) 4.28
Javanbakht (2023) 0.871 (0.846, 0.893) 4.25
Muhindo (2022) 0.696 (0.663, 0.728) 4.25
Bert (2022) 0.856 (0.794, 0.902) 4.14
Shallangwa (2023) 0.256 (0.213, 0.304) 4.22
Kabir (2022) 0.135 (0.113, 0.161) 4.25
Lv (2023) 0.883 (0.866, 0.899) 4.27
Rosenthal (2023) 0.721 (0.718, 0.724) 4.29
Strathdee (2023) 0.533 (0.301, 0.752) 3.13
Costiniuk (2023) 0.890 (0.845, 0.923) 4.19
Overall (I^2 = 99.829%, p = 0.000) 0.587 (0.502, 0.669) 100.00
0
.5
1
COVID-19 Vaccine Uptake Among PLHIV (Globally)

Supplementary Figure 30: Forest plots of the results of random-effects model meta-analysis of the prevalence (%) of COVID-19 vaccine uptake among PLHIV globally (n = 24 studies) pooled using inverse variance weights. Each black-colored solid square represents the effect size of each characteristic, while the ends of the adjoining horizontal lines represent lower (left) and upper (right) confidence intervals. The blue-colored hollow diamond at the bottom denotes the overall estimated effect size and the 95% CI. All statistics were based on a two-sided t-test. ES, Effect Size.

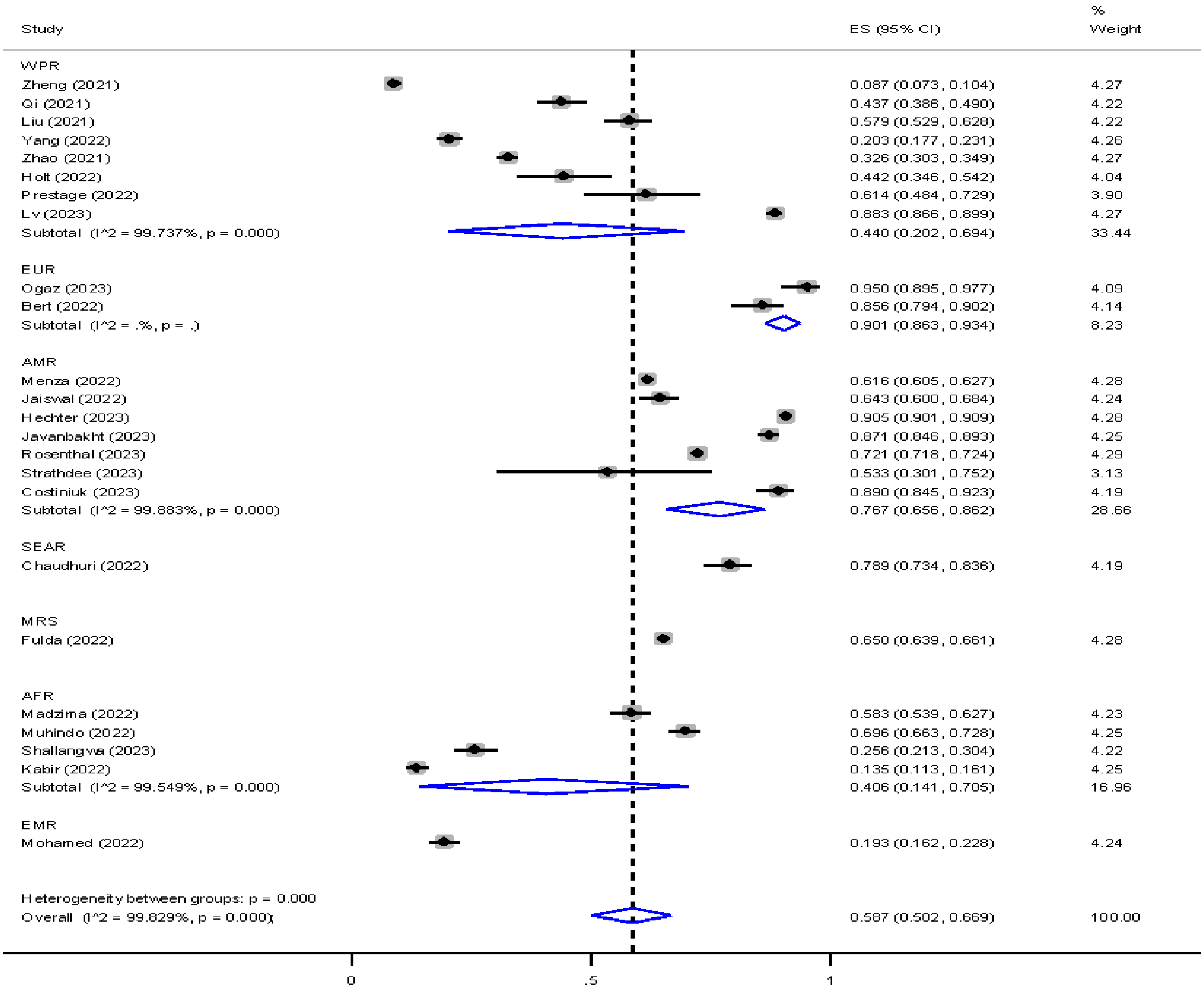

COVID-19 Vaccine uptake Among PLHIV (By WHO Region)

Supplementary Figure 31: Forest plots of the results of random-effects model meta-analysis of the prevalence (%) of COVID-19 vaccine uptake among PLHIV according to the WHO region (n = 24 studies) pooled using inverse variance weights. Each black-colored solid square represents the effect size of each characteristic, while the ends of the adjoining horizontal lines represent lower (left) and upper (right) confidence intervals. The blue-colored hollow diamond at the bottom denotes the overall estimated effect size and the 95% CI. All statistics were based on a two-sided t-test. ES, Effect Size.

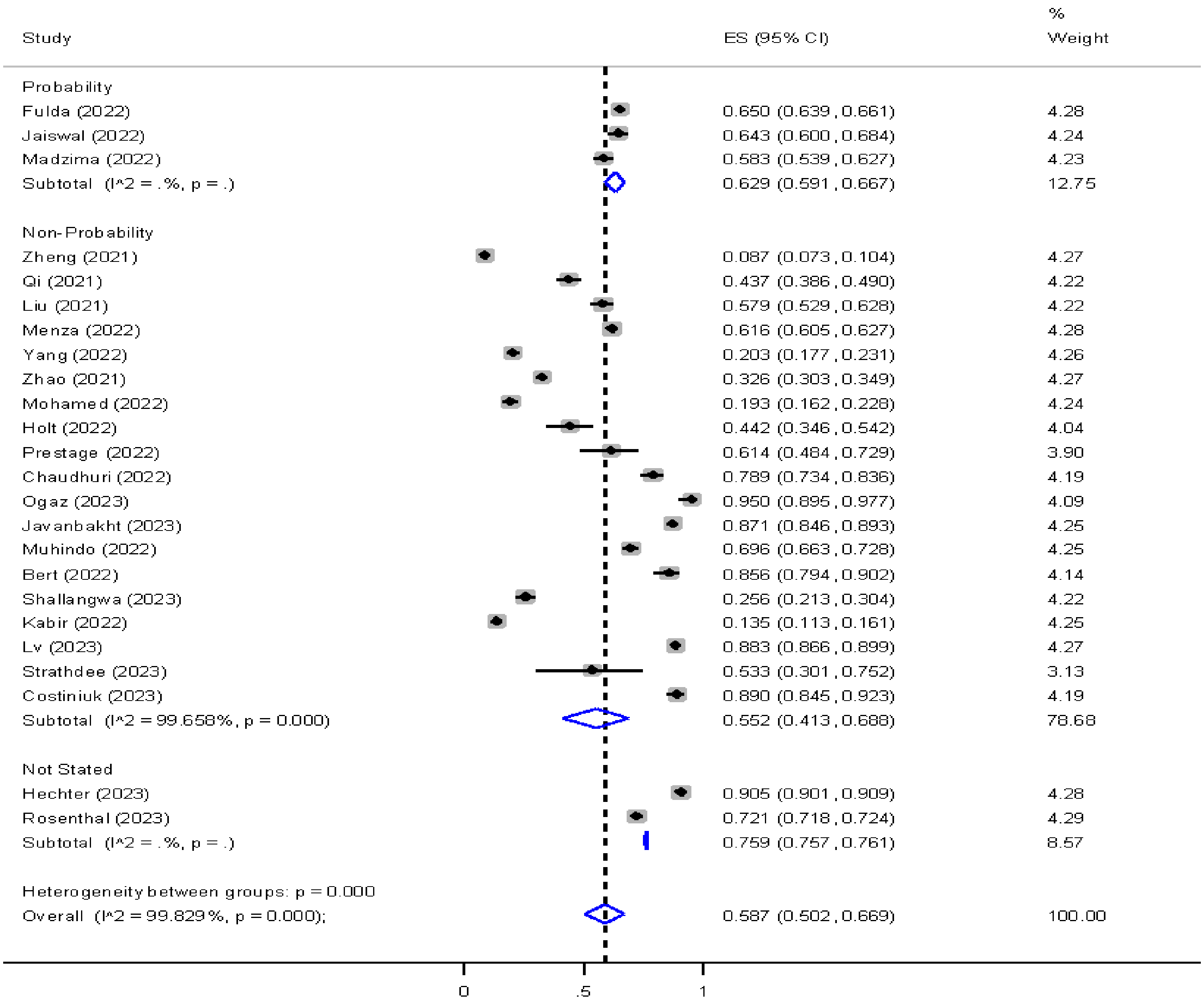

COVID-19 Vaccine Uptake Among PLHIV (By Sampling Method)

Supplementary Figure 32: Forest plots of the results of random-effects model meta-analysis of the prevalence (%) of COVID-19 vaccine uptake among PLHIV according to the sampling method (n = 24 studies) pooled using inverse variance weights. Each black-colored solid square represents the effect size of each characteristic, while the ends of the adjoining horizontal lines represent lower (left) and upper (right) confidence intervals. The blue-colored hollow diamond at the bottom denotes the overall estimated effect size and the 95% CI. All statistics were based on a two-sided t-test. ES, Effect Size.

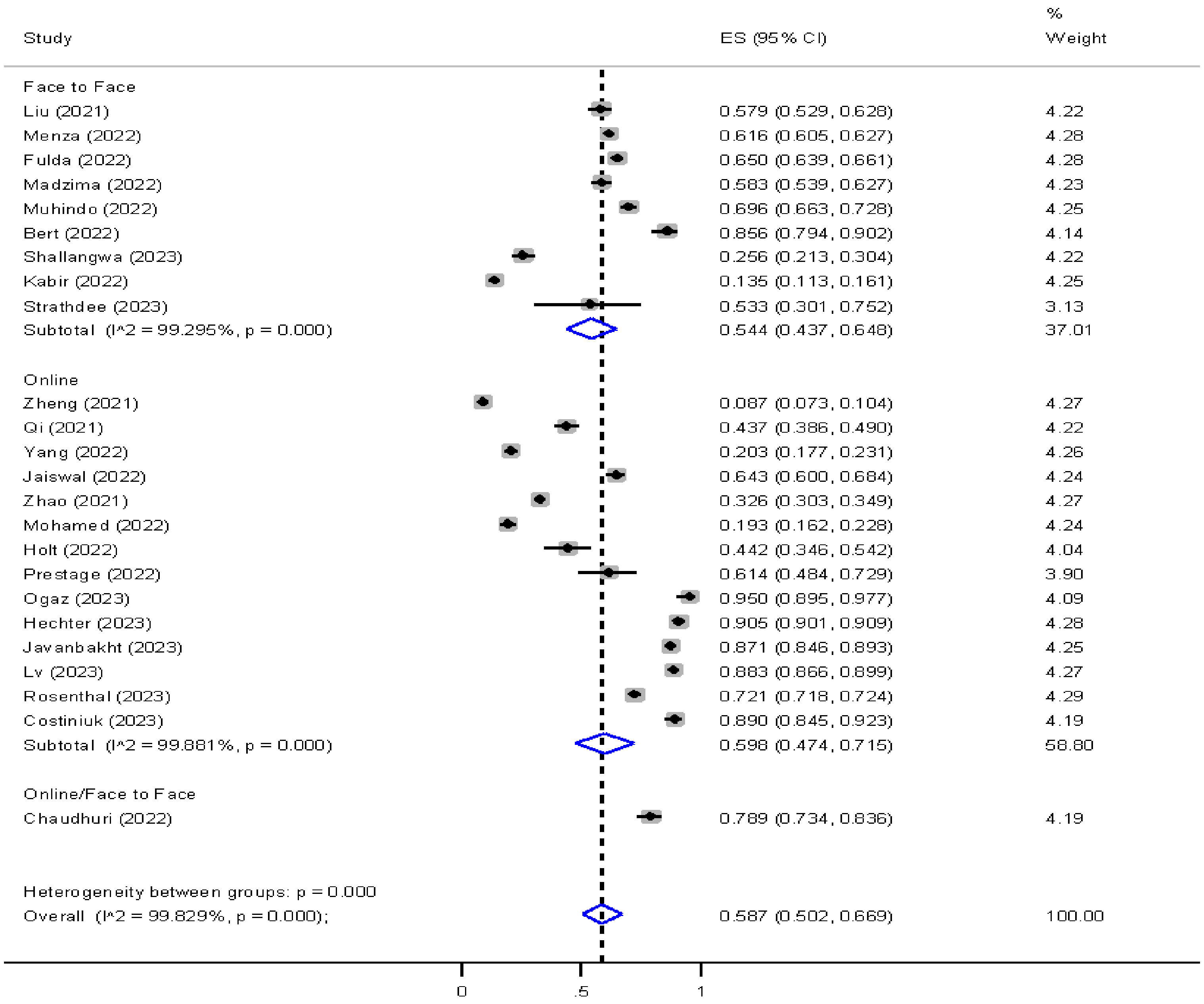

COVID-19 Vaccine uptake Among PLHIV (By Data Collection Method)

Supplementary Figure 33: Forest plots of the results of random-effects model meta-analysis of the prevalence (%) of COVID-19 vaccine uptake among PLHIV according to the data collection method (n = 24 studies) pooled using inverse variance weights. Each black-colored solid square represents the effect size of each characteristic, while the ends of the adjoining horizontal lines represent lower (left) and upper (right) confidence intervals. The blue-colored hollow diamond at the bottom denotes the overall estimated effect size and the 95% CI. All statistics were based on a two-sided t-test. ES, Effect Size.

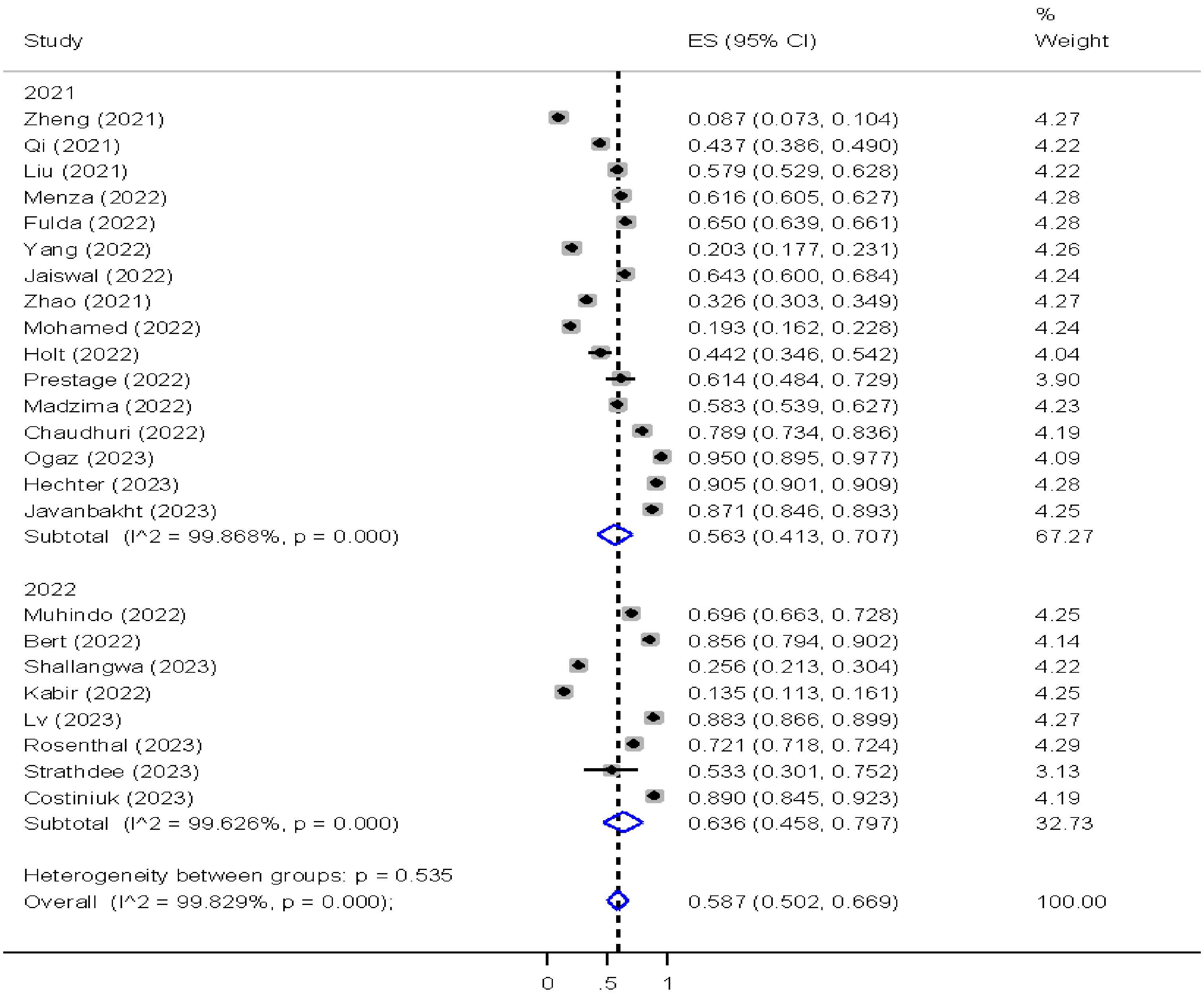

COVID-19 Vaccine Uptake Among PLHIV (By Study Year)

Supplementary Figure 34: Forest plots of the results of random-effects model meta-analysis of the prevalence (%) of COVID-19 vaccine uptake among PLHIV according to the study year (n = 24 studies) pooled using inverse variance weights. Each black-colored solid square represents the effect size of each characteristic, while the ends of the adjoining horizontal lines represent lower (left) and upper (right) confidence intervals. The blue-colored hollow diamond at the bottom denotes the overall estimated effect size and the 95% CI. All statistics were based on a two-sided t-test. ES, Effect Size.

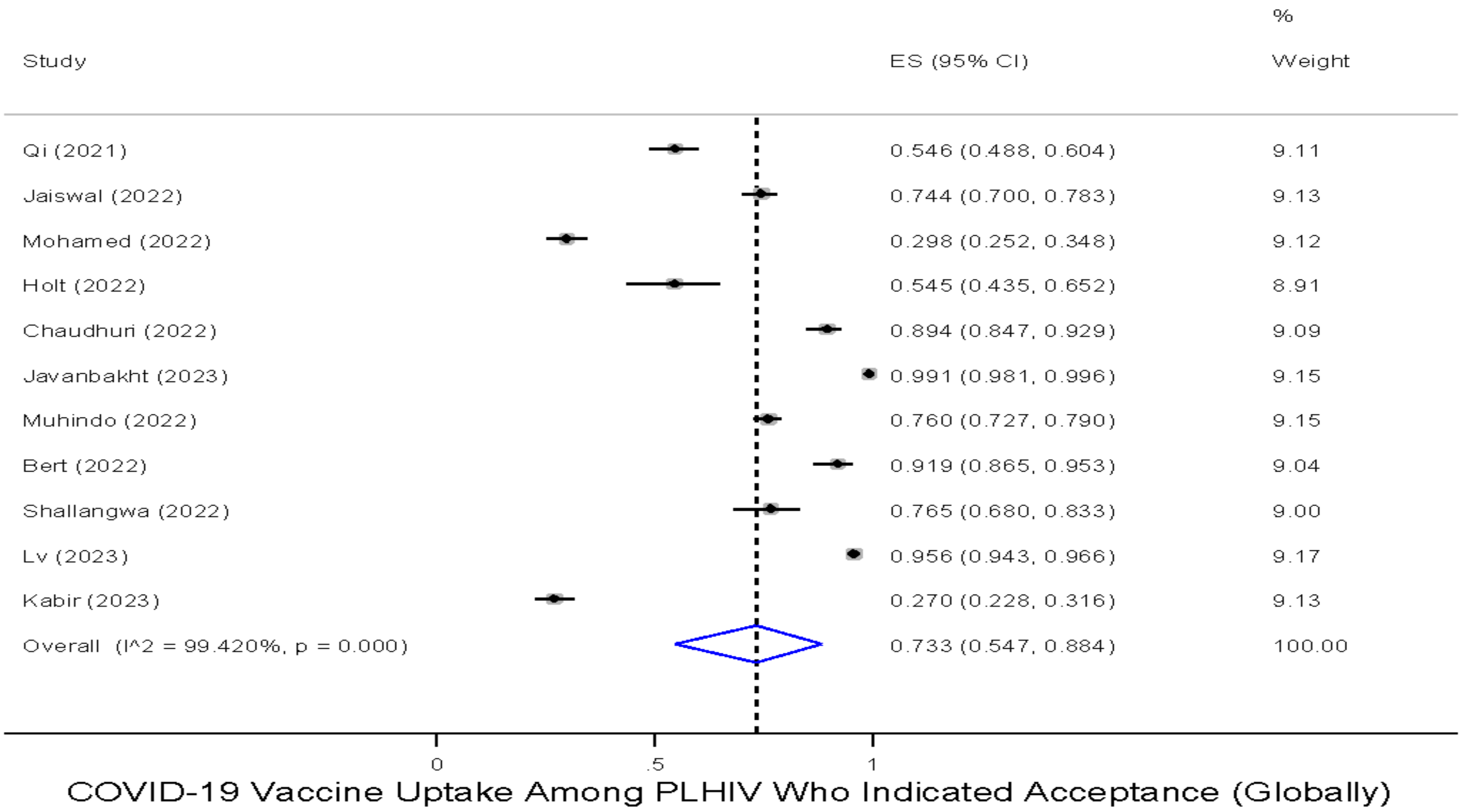

Supplementary Figure 35: Forest plots of the results of random-effects model meta-analysis of the prevalence (%) of COVID-19 vaccine uptake among PLHIV who indicated acceptance globally (n = 11 studies) pooled using inverse variance weights. Each black-colored solid square represents the effect size of each characteristic, while the ends of the adjoining horizontal lines represent lower (left) and upper (right) confidence intervals. The blue-colored hollow diamond at the bottom denotes the overall estimated effect size and the 95% CI. All statistics were based on a two-sided t-test. ES, Effect Size.

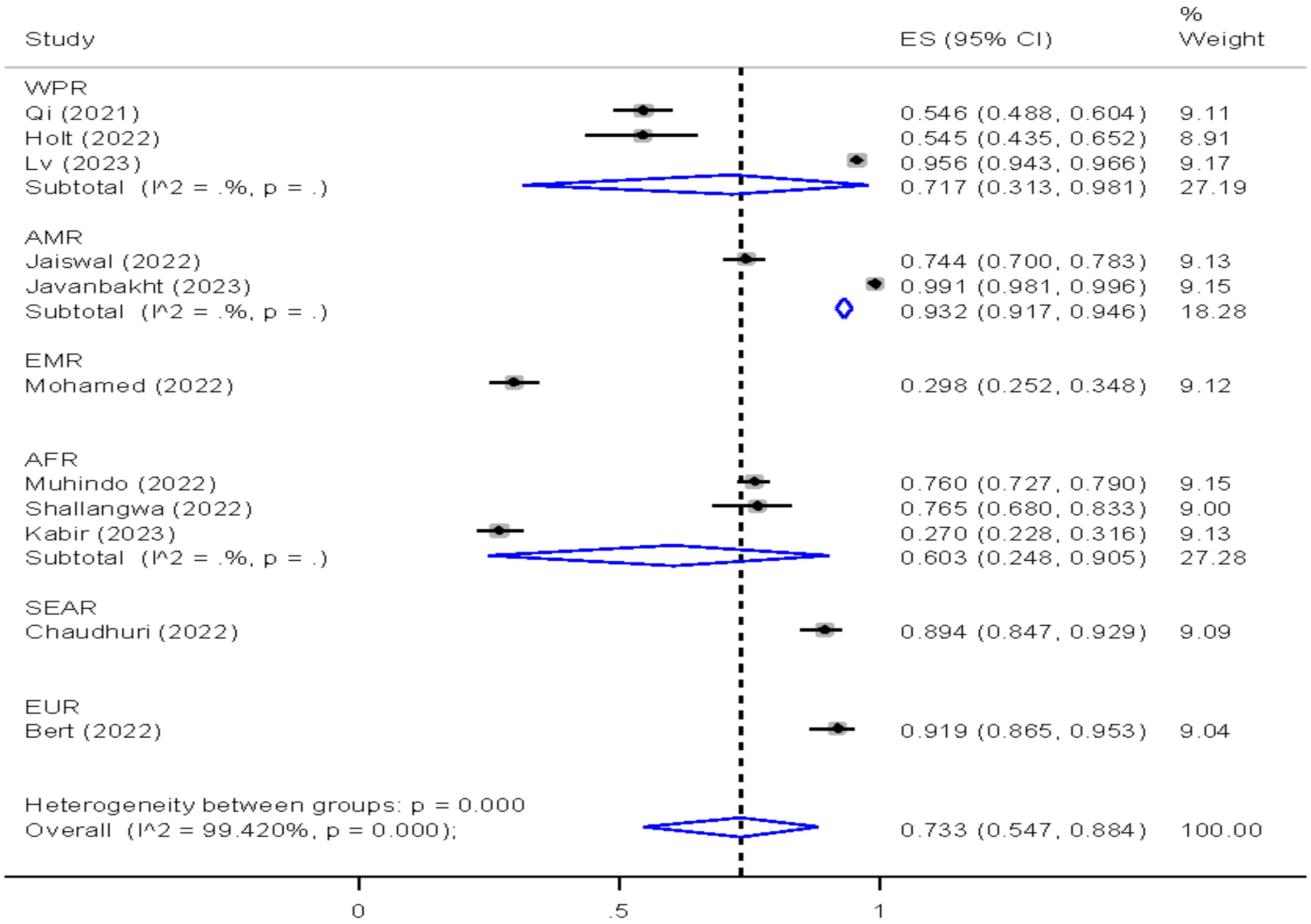

D-19 Vaccine uptake Among PLHIV Who Indicated Acceptance (By WHO Region)

Supplementary Figure 36: Forest plots of the results of random-effects model meta-analysis of the prevalence (%) of COVID-19 vaccine uptake among PLHIV who indicated acceptance according to the WHO region (n = 11 studies) pooled using inverse variance weights. Each black-colored solid square represents the effect size of each characteristic, while the ends of the adjoining horizontal lines represent lower (left) and upper (right) confidence intervals. The blue-colored hollow diamond at the bottom denotes the overall estimated effect size and the 95% CI. All statistics were based on a two-sided t-test. ES, Effect Size.

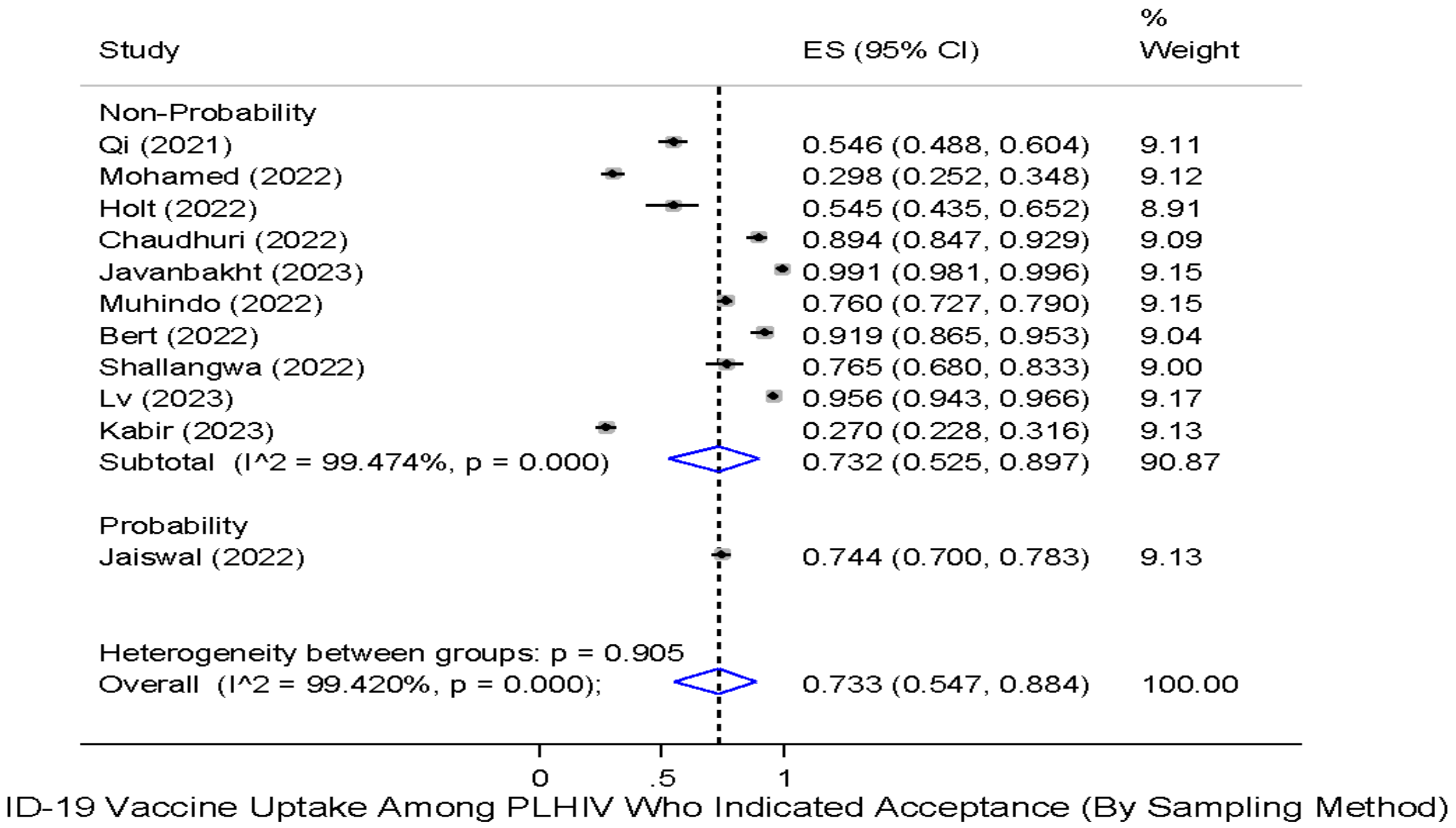

Supplementary Figure 37: Forest plots of the results of random-effects model meta-analysis of the prevalence (%) of COVID-19 vaccine uptake among PLHIV who indicated acceptance according to the sampling method employed (n = 11 studies) pooled using inverse variance weights. Each black-colored solid square represents the effect size of each characteristic, while the ends of the adjoining horizontal lines represent lower (left) and upper (right) confidence intervals. The blue-colored hollow

diamond at the bottom denotes the overall estimated effect size and the 95% CI. All statistics were based on a two-sided t-test. ES, Effect Size.

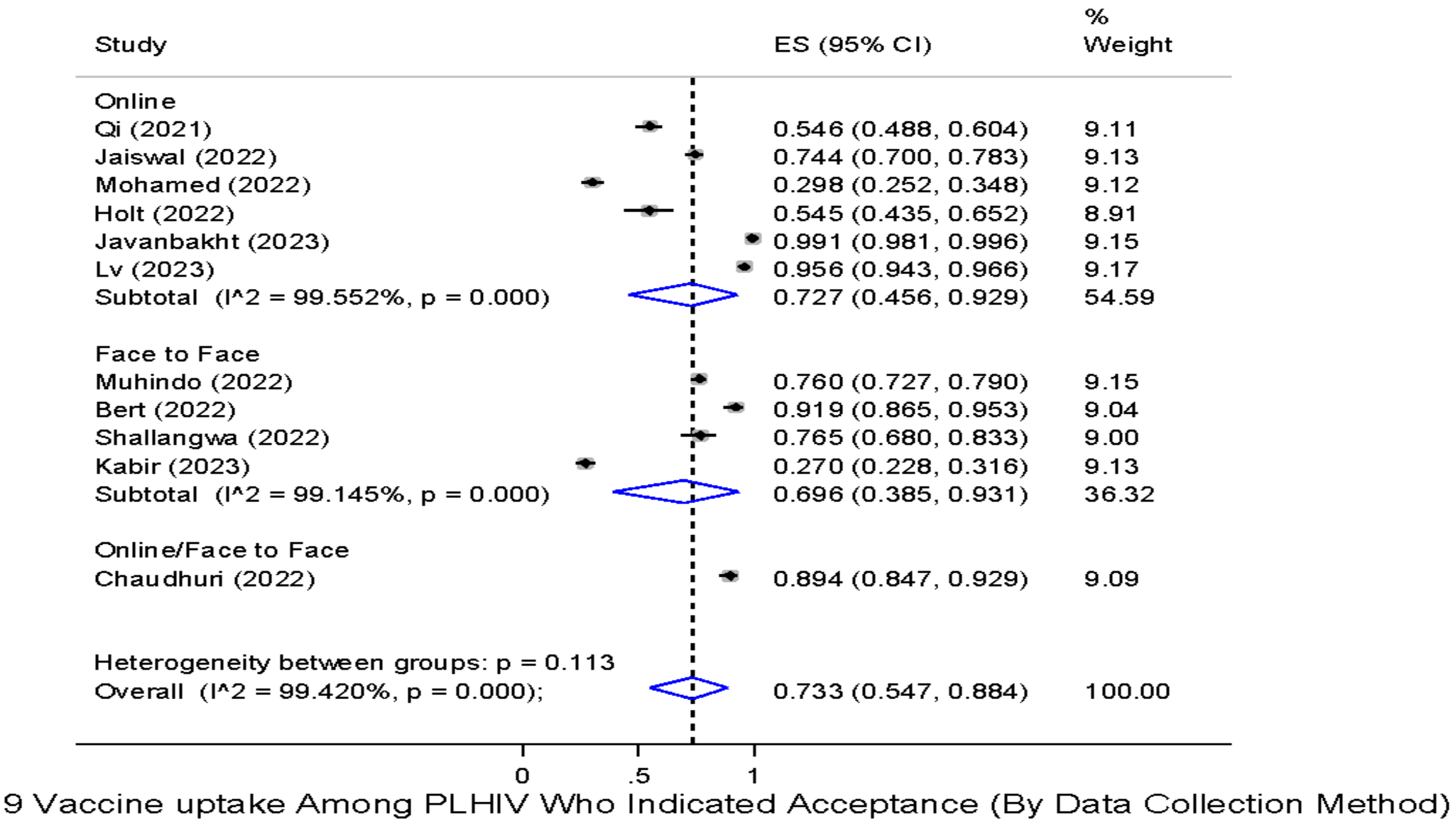

Supplementary Figure 38: Forest plots of the results of random-effects model meta-analysis of the prevalence (%) of COVID-19 vaccine uptake among PLHIV who indicated acceptance according to the data collection method (n = 11 studies) pooled using inverse variance weights. Each black-colored solid square represents the effect size of each characteristic, while the ends of the adjoining horizontal lines represent lower (left) and upper (right) confidence intervals. The blue-colored hollow diamond at the bottom denotes the overall estimated effect size and the 95% CI. All statistics were based on a two-sided t-test. ES, Effect Size.

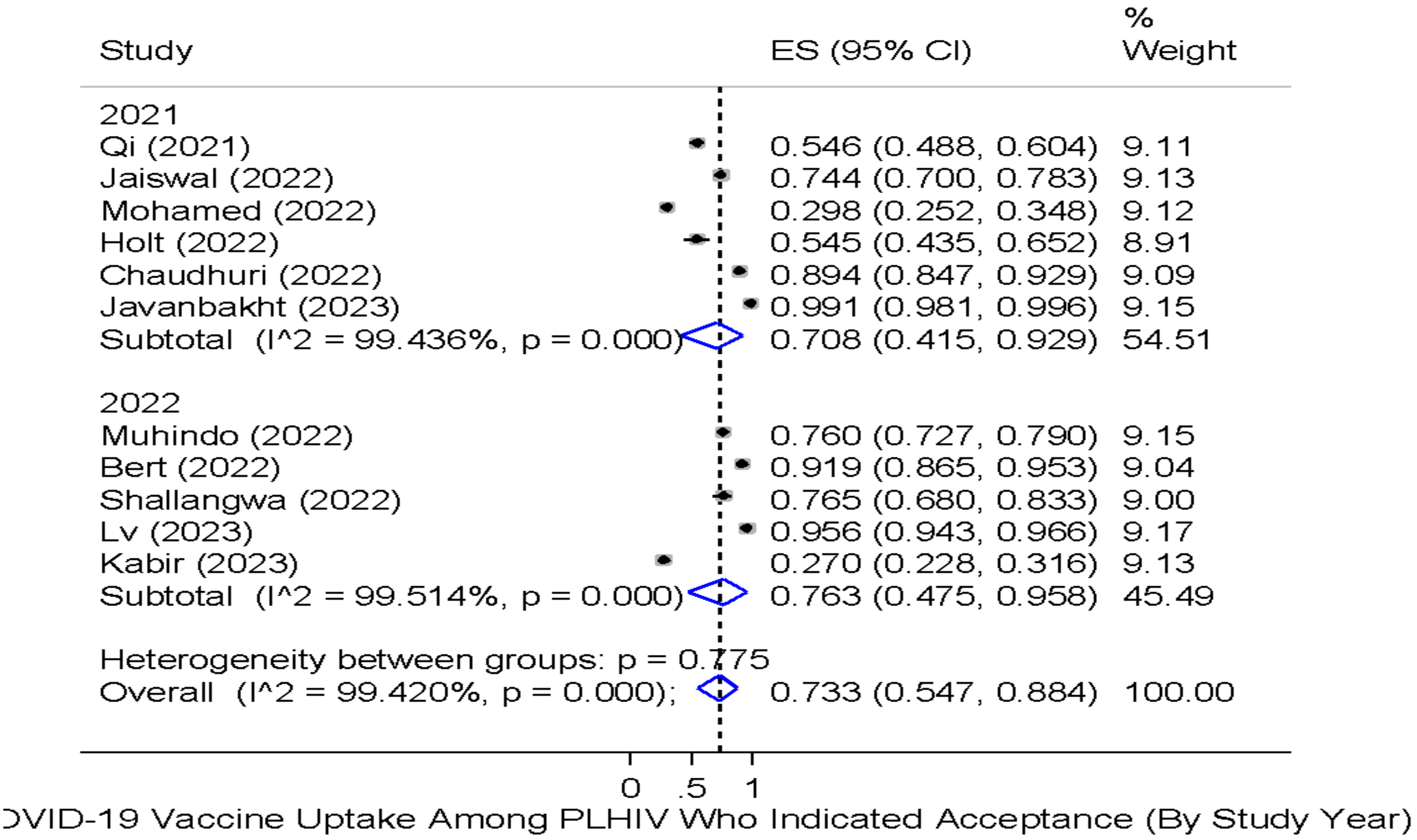

Supplementary Figure 39: Forest plots of the results of random-effects model meta-analysis of the prevalence (%) of COVID-19 vaccine uptake among PLHIV who indicated acceptance according to the study year (n = 11 studies) pooled using inverse variance weights. Each black-colored solid square represents the effect size of each characteristic, while the ends of the

adjoining horizontal lines represent lower (left) and upper (right) confidence intervals. The blue-colored hollow diamond at the bottom denotes the overall estimated effect size and the 95% CI. All statistics were based on a two-sided t-test. ES, Effect Size.

SECTION D

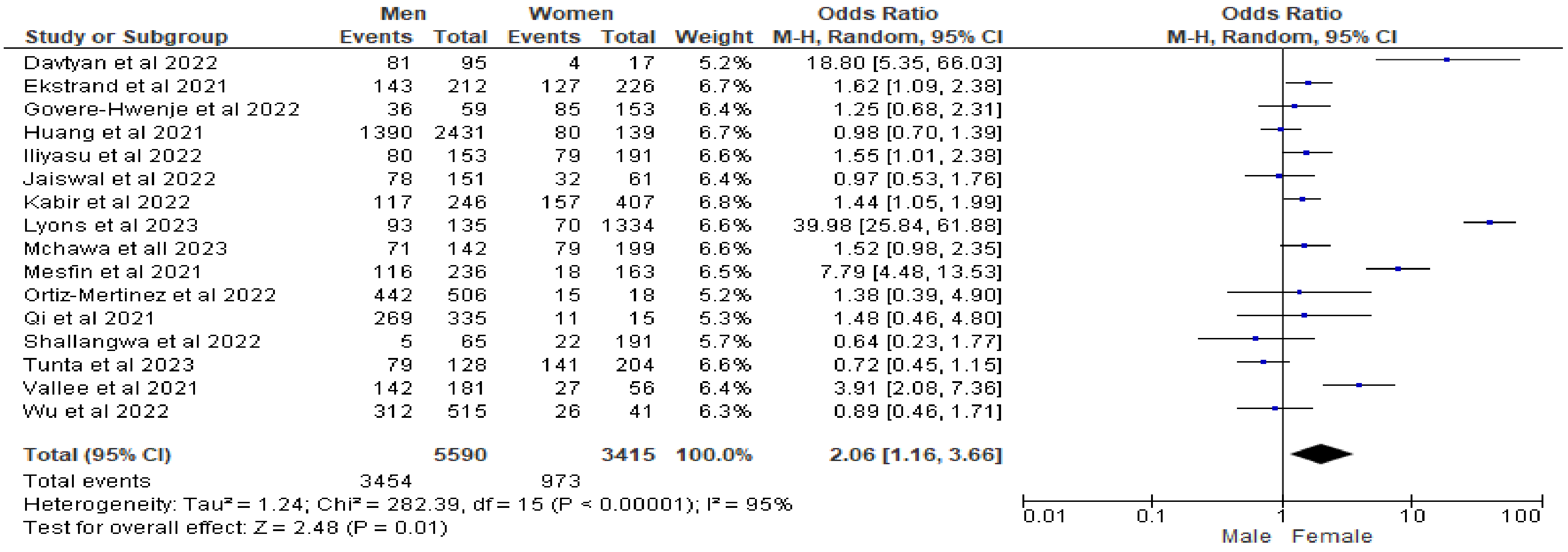

Study or Subgroup	Men Events	Men Total	Women Events	Women Total	Weight	Odds Ratio M-H, Random, 95% CI
Davtyan et al 2022	81	95	4	17	5.2%	18.80 [5.35, 66.03]
Ekstrand et al 2021	143	212	127	226	6.7%	1.62 [1.09, 2.38]
Govere-Hwenje et al 2022	36	59	85	153	6.4%	1.25 [0.68, 2.31]
Huang et al 2021	1390	2431	80	139	6.7%	0.98 [0.70, 1.39]
Iliyasu et al 2022	80	153	79	191	6.6%	1.55 [1.01, 2.38]
Jaiswal et al 2022	78	151	32	61	6.4%	0.97 [0.53, 1.76]
Kabir et al 2022	117	246	157	407	6.8%	1.44 [1.05, 1.99]
Lyons et al 2023	93	135	70	1334	6.6%	39.98 [25.84, 61.88]
Mchawa et all 2023	71	142	79	199	6.6%	1.52 [0.98, 2.35]
Mesfin et al 2021	116	236	18	163	6.5%	7.79 [4.48, 13.53]
Ortiz-Mertinez et al 2022	442	506	15	18	5.2%	1.38 [0.39, 4.90]
Qi et al 2021	269	335	11	15	5.3%	1.48 [0.46, 4.80]
Shallangwa et al 2022	5	65	22	191	5.7%	0.64 [0.23, 1.77]
Tunta et al 2023	79	128	141	204	6.6%	0.72 [0.45, 1.15]
Vallee et al 2021	142	181	27	56	6.4%	3.91 [2.08, 7.36]
Wu et al 2022	312	515	26	41	6.3%	0.89 [0.46, 1.71]
**Total (95% CI)**		**5590**		**3415**	**100.0%**	**2.06 [1.16, 3.66]**
Total events	3454		973			

Heterogeneity: Tau² = 1.24; Chi² = 282.39, df = 15 (P < 0.00001); I² = 95%
Test for overall effect: Z = 2.48 (P = 0.01)

Supplementary Figure 40: Forest plots of random-effects model meta-analysis of gender as a determinant of COVID-19 vaccine acceptance pooled using the Mantel-Haenszel (M-H) method (n = 16 studies; N = 9,005 participants). Each blue-colored solid square represents the effect size (odds ratio, OR) of each characteristic, while the ends of the adjoining horizontal lines represent

lower (left) and upper (right) confidence intervals. The isolated black-colored solid rhombus at the bottom denotes the overall estimated effect size and the 95% CI. All statistics were based on a two-sided t-test. df: degree of freedom.

	< 40 years		>= 40 years			Odds Ratio
**Study or Subgroup**	**Events**	**Total**	**Events**	**Total**	**Weight**	**M-H, Random, 95% CI**
Govere-Hwenje et al 2022	75	147	46	65	8.9%	0.43 [0.23, 0.80]
Huang et al 2021	1094	1916	376	654	17.3%	0.98 [0.82, 1.18]
Iliyasu et al 2022	51	121	81	166	11.5%	0.76 [0.48, 1.23]
Kabir et al 2022	132	339	197	363	15.0%	0.54 [0.40, 0.73]
Liu et al 2021	30	241	22	82	9.0%	0.39 [0.21, 0.72]
Mchawa et all 2023	100	243	50	98	11.6%	0.67 [0.42, 1.08]
Ortiz-Mertinez et al 2022	352	401	108	127	9.8%	1.26 [0.71, 2.24]
Qi et al 2021	200	255	80	95	8.9%	0.68 [0.36, 1.28]
Vallee et al 2021	31	46	138	191	8.0%	0.79 [0.40, 1.59]
**Total (95% CI)**		**3709**		**1841**	**100.0%**	**0.70 [0.54, 0.90]**
Total events	2065		1098			

Heterogeneity: $Tau^2 = 0.09$; $Chi^2 = 24.01$, df = 8 (P = 0.002); $I^2 = 67\%$
Test for overall effect: Z = 2.77 (P = 0.006)

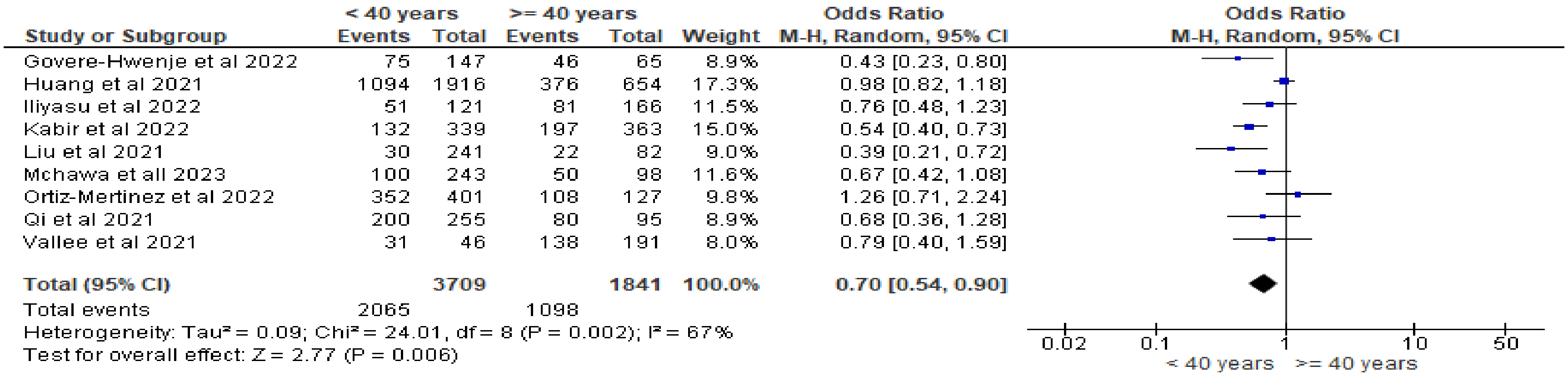

Supplementary Figure 41: Forest plots of random-effects model meta-analysis of age as a determinant of COVID-19 vaccine acceptance pooled using the Mantel-Haenszel (M-H) method (n = 9 studies; N = 5,550 participants). Each blue-colored solid square represents the effect size (odds ratio, OR) of each characteristic, while the ends of the adjoining horizontal lines represent lower (left) and upper (right) confidence intervals. The isolated black-colored solid rhombus at the bottom denotes the overall estimated effect size and the 95% CI. All statistics were based on a two-sided t-test. df: degree of freedom.

Study or Subgroup	Single/divorced/widowed Events	Total	Married/cohabiting Events	Total	Weight	Odds Ratio M-H, Random, 95% CI
Davtyan et al 2022	44	54	42	60	3.3%	1.89 [0.78, 4.55]
Huang et al 2021	993	1750	477	820	15.1%	0.94 [0.80, 1.12]
Iliyasu et al 2022	58	100	101	244	7.9%	1.96 [1.22, 3.13]
Kabir et al 2022	103	264	170	383	11.2%	0.80 [0.58, 1.10]
Liu et al 2021	81	297	20	69	6.2%	0.92 [0.51, 1.64]
Lv et al 2023	941	1080	315	344	8.9%	0.62 [0.41, 0.95]
Lyons et al 2023	112	178	51	91	7.2%	1.33 [0.80, 2.22]
Mchawa et all 2023	50	107	100	234	8.1%	1.18 [0.74, 1.86]
Mesfin et al 2021	54	165	80	233	8.8%	0.93 [0.61, 1.42]
Qi et al 2021	214	268	66	82	5.6%	0.96 [0.52, 1.79]
Shallangwa et al 2022	11	128	16	128	3.8%	0.66 [0.29, 1.48]
Tunta et al 2023	183	287	37	45	3.9%	0.38 [0.17, 0.85]
Wu et al 2022	238	395	100	161	9.8%	0.92 [0.63, 1.35]
**Total (95% CI)**		**5073**		**2894**	**100.0%**	**0.96 [0.80, 1.15]**
Total events	3082		1575			

Heterogeneity: Tau² = 0.05; Chi² = 24.61, df = 12 (P = 0.02); I² = 51%
Test for overall effect: Z = 0.47 (P = 0.64)

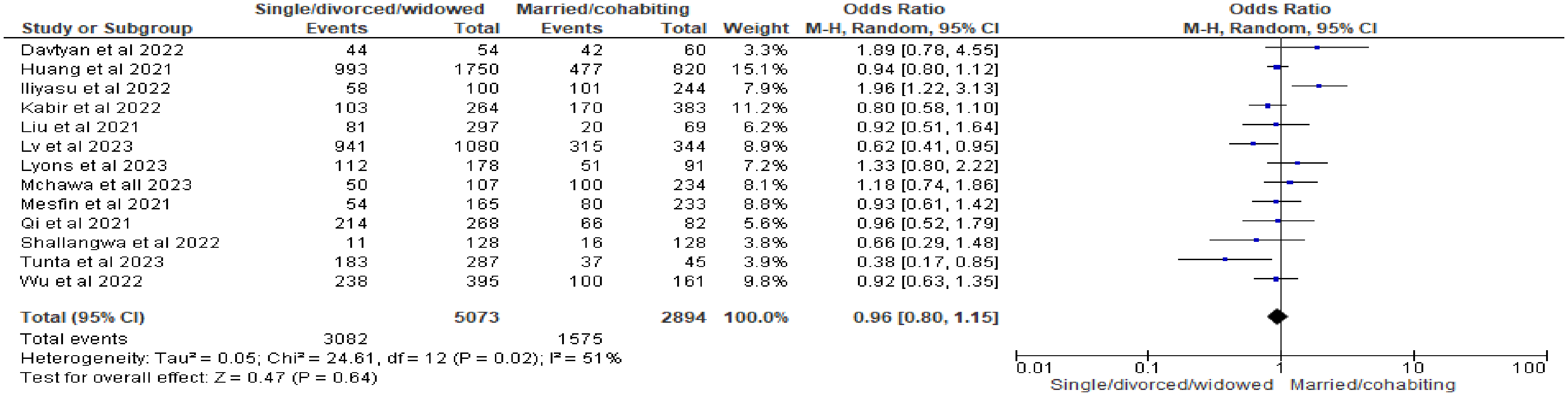

Supplementary Figure 42: Forest plots of random-effects model meta-analysis of marital status as a determinant of COVID-19 vaccine acceptance pooled using the Mantel-Haenszel (M-H) method (n = 13 studies; N = 7,967 participants). Each blue-colored solid square represents the effect size (odds ratio, OR) of each characteristic, while the ends of the adjoining horizontal lines represent lower (left) and upper (right) confidence intervals. The isolated black-colored solid rhombus at the bottom denotes the overall estimated effect size and the 95% CI. All statistics were based on a two-sided t-test. df: degree of freedom.

Study or Subgroup	Black Events	Total	White Events	Total	Weight	Odds Ratio M-H, Fixed, 95% CI
Davtyan et al 2022	7	14	74	88	38.4%	0.19 [0.06, 0.62]
Jaiswal et al 2022	32	54	44	65	61.6%	0.69 [0.33, 1.47]
**Total (95% CI)**		**68**		**153**	**100.0%**	**0.50 [0.27, 0.94]**
Total events	39		118			

Heterogeneity: Chi² = 3.28, df = 1 (P = 0.07); I² = 70%
Test for overall effect: Z = 2.14 (P = 0.03)

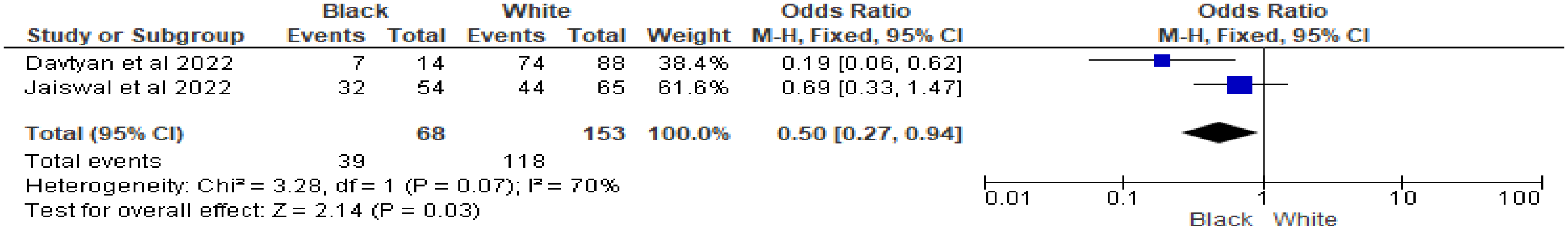

Supplementary Figure 43: Forest plots of fixed-effects model meta-analysis of race (Black vs. White) as a determinant of COVID-19 vaccine acceptance pooled using the Mantel-Haenszel (M-H) method (n = 2 studies; N = 221 participants). Each blue-colored solid square represents the effect size (odds ratio, OR) of each characteristic, while the ends of the adjoining horizontal

lines represent lower (left) and upper (right) confidence intervals. The isolated black-colored solid rhombus at the bottom denotes the overall estimated effect size and the 95% CI. All statistics were based on a two-sided t-test. df: degree of freedom.

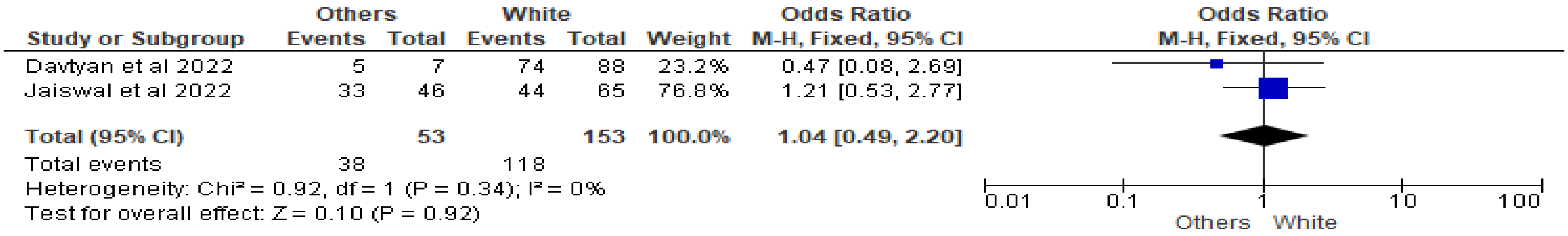

Supplementary Figure 44: Forest plots of fixed-effects model meta-analysis of race (Others [Latinx/Hispanic/Mixed race] vs. White people) as a determinant of COVID-19 vaccine acceptance pooled using the Mantel-Haenszel (M-H) method (n = 2 studies; N = 206 participants). Each blue-colored solid square represents the effect size (odds ratio, OR) of each characteristic, while the ends of the adjoining horizontal lines represent lower (left) and upper (right) confidence intervals. The isolated black-colored solid rhombus at the bottom denotes the overall estimated effect size and the 95% CI. All statistics were based on a two-sided t-test. df: degree of freedom.

Study or Subgroup	Primary and below Events	Primary and below Total	Secondary and above Events	Secondary and above Total	Weight	Odds Ratio M-H, Random, 95% CI
Ekstrand et al 2021	140	217	91	221	7.5%	2.60 [1.77, 3.82]
Huang et al 2021	249	425	1221	2145	7.9%	1.07 [0.87, 1.32]
Iliyasu et al 2022	15	54	144	290	6.7%	0.39 [0.21, 0.74]
Jaiswal et al 2022	33	50	77	106	6.4%	0.73 [0.35, 1.51]
Kabir et al 2022	187	438	87	214	7.6%	1.09 [0.78, 1.52]
Liu et al 2021	21	222	81	145	7.0%	0.08 [0.05, 0.14]
Lv et al 2023	239	273	1077	1151	7.4%	0.48 [0.31, 0.74]
Lyons et al 2023	28	40	134	228	6.4%	1.64 [0.79, 3.38]
Mchawa et all 2023	72	201	78	140	7.3%	0.44 [0.29, 0.69]
Mesfin et al 2021	62	209	72	189	7.4%	0.69 [0.45, 1.04]
Qi et al 2021	70	87	210	263	6.8%	1.04 [0.56, 1.91]
Su et al 2022	207	332	451	571	7.7%	0.44 [0.33, 0.59]
Tunta et al 2023	146	249	74	83	6.3%	0.17 [0.08, 0.36]
Wu et al 2022	119	315	139	241	7.6%	0.45 [0.32, 0.63]
**Total (95% CI)**		**3112**		**5987**	**100.0%**	**0.60 [0.40, 0.89]**
Total events	1588		3936			

Heterogeneity: $Tau^2 = 0.53$; $Chi^2 = 170.30$, df = 13 ($P < 0.00001$); $I^2 = 92\%$
Test for overall effect: Z = 2.51 (P = 0.01)

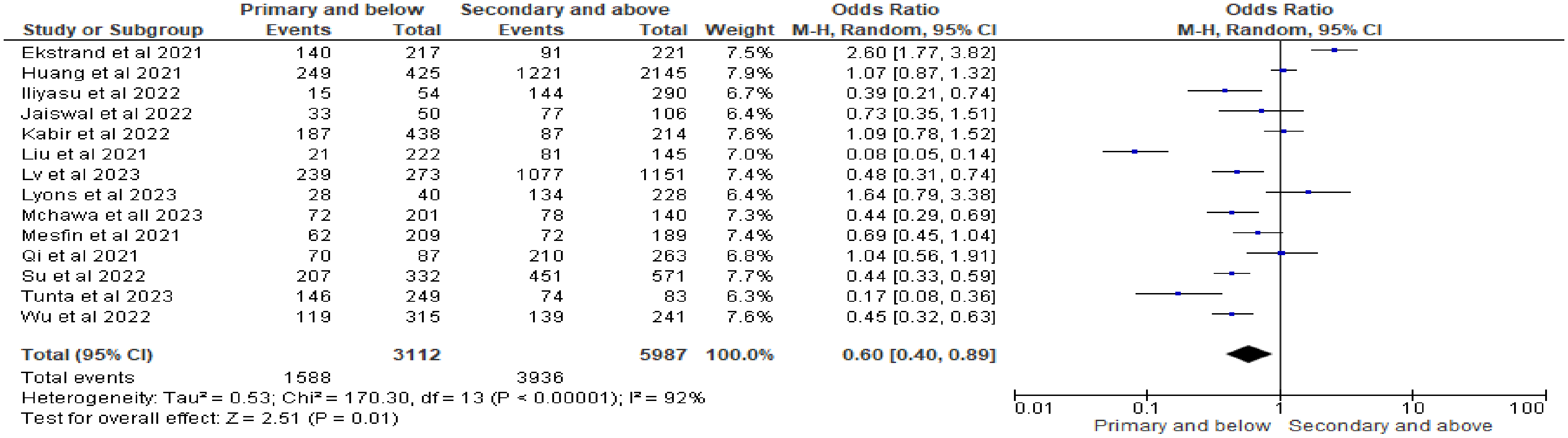

Supplementary Figure 45: Forest plots of random-effects model meta-analysis of educational level as a determinant of COVID-19 vaccine acceptance pooled using the Mantel-Haenszel (M-H) method (n = 14 studies; N = 9,099 participants). Each blue-colored solid square represents the effect size (odds ratio, OR) of each characteristic, while the ends of the adjoining horizontal lines represent lower (left) and upper (right) confidence intervals. The isolated black-colored solid rhombus at the bottom denotes the overall estimated effect size and the 95% CI. All statistics were based on a two-sided t-test. df: degree of freedom.

Study or Subgroup	Unemployed Events	Unemployed Total	Employed Events	Employed Total	Weight	Odds Ratio M-H, Random, 95% CI
Davtyan et al 2022	56	71	30	43	7.4%	1.62 [0.68, 3.84]
Huang et al 2021	448	788	1022	1782	13.4%	0.98 [0.83, 1.16]
Iliyasu et al 2022	23	64	136	280	10.1%	0.59 [0.34, 1.04]
Jaiswal et al 2022	51	77	59	89	9.3%	1.00 [0.52, 1.90]
Kabir et al 2022	210	473	62	173	12.0%	1.43 [1.00, 2.05]
Liu et al 2021	4	18	98	350	5.5%	0.73 [0.24, 2.29]
Lyons et al 2023	73	133	90	136	10.8%	0.62 [0.38, 1.02]
Mchawa et all 2023	36	59	114	282	10.0%	2.31 [1.30, 4.10]
Mesfin et al 2021	70	274	64	124	11.3%	0.32 [0.21, 0.50]
Shallangwa et al 2022	21	210	6	46	6.6%	0.74 [0.28, 1.95]
Tunta et al 2023	10	12	210	320	3.7%	2.62 [0.56, 12.16]
**Total (95% CI)**		**2179**		**3625**	**100.0%**	**0.93 [0.66, 1.31]**
Total events	1002		1891			

Heterogeneity: $Tau^2 = 0.22$; $Chi^2 = 46.08$, df = 10 ($P < 0.00001$); $I^2 = 78\%$
Test for overall effect: Z = 0.42 (P = 0.67)

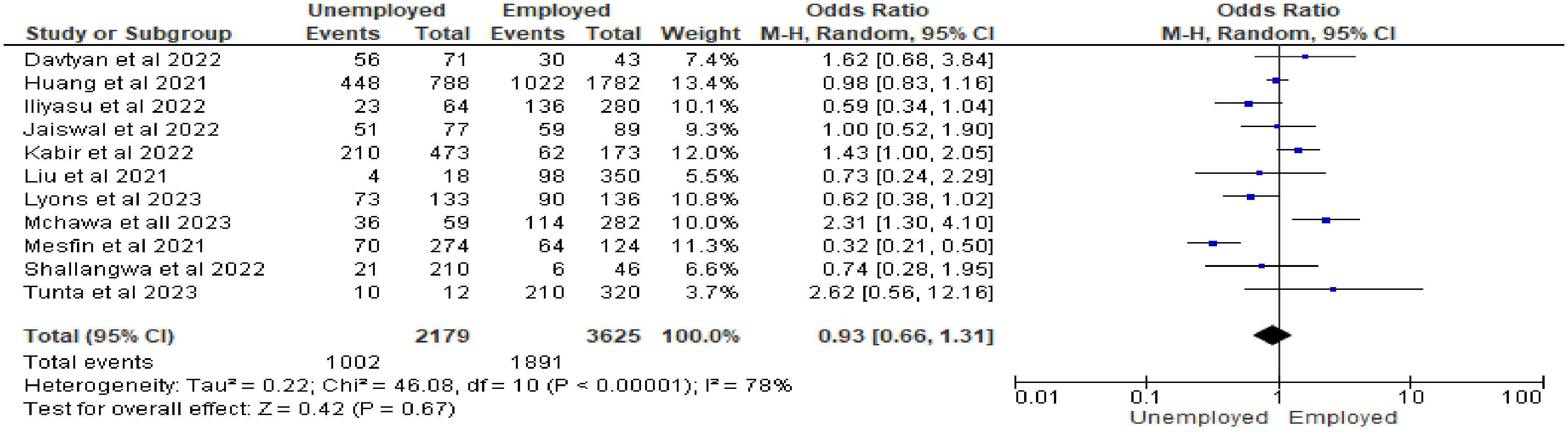

Supplementary Figure 46: Forest plots of random-effects model meta-analysis of employment status as a determinant of COVID-19 vaccine acceptance pooled using the Mantel-Haenszel (M-H) method (n = 11 studies; N = 5,804 participants). Each blue-colored solid square represents the effect size (odds ratio, OR) of each characteristic, while the ends of the adjoining horizontal lines represent lower (left) and upper (right) confidence intervals. The isolated black-colored solid rhombus at the bottom denotes the overall estimated effect size and the 95% CI. All statistics were based on a two-sided t-test. df: degree of freedom.

1. Income class

	Low		Medium/high			Odds Ratio
**Study or Subgroup**	**Events**	**Total**	**Events**	**Total**	**Weight**	**M-H, Random, 95% CI**
Davtyan et al 2022	26	27	58	85	1.4%	12.10 [1.56, 93.91]
Huang et al 2021	246	438	1224	2132	16.7%	0.95 [0.77, 1.17]
Iliyasu et al 2022	83	207	76	137	12.1%	0.54 [0.35, 0.83]
Kabir et al 2022	225	529	40	100	12.2%	1.11 [0.72, 1.72]
Lv et al 2023	662	718	654	706	13.0%	0.94 [0.63, 1.39]
Mchawa et all 2023	120	273	30	68	10.3%	0.99 [0.58, 1.70]
Mesfin et al 2021	19	76	115	322	9.8%	0.60 [0.34, 1.06]
Qi et al 2021	111	141	169	209	10.4%	0.88 [0.52, 1.49]
Wu et al 2022	191	289	147	267	14.0%	1.59 [1.13, 2.24]
**Total (95% CI)**		**2698**		**4026**	**100.0%**	**0.96 [0.74, 1.24]**
Total events	1683		2513			

Heterogeneity: Tau² = 0.09; Chi² = 24.25, df = 8 (P = 0.002); I² = 67%
Test for overall effect: Z = 0.33 (P = 0.74)

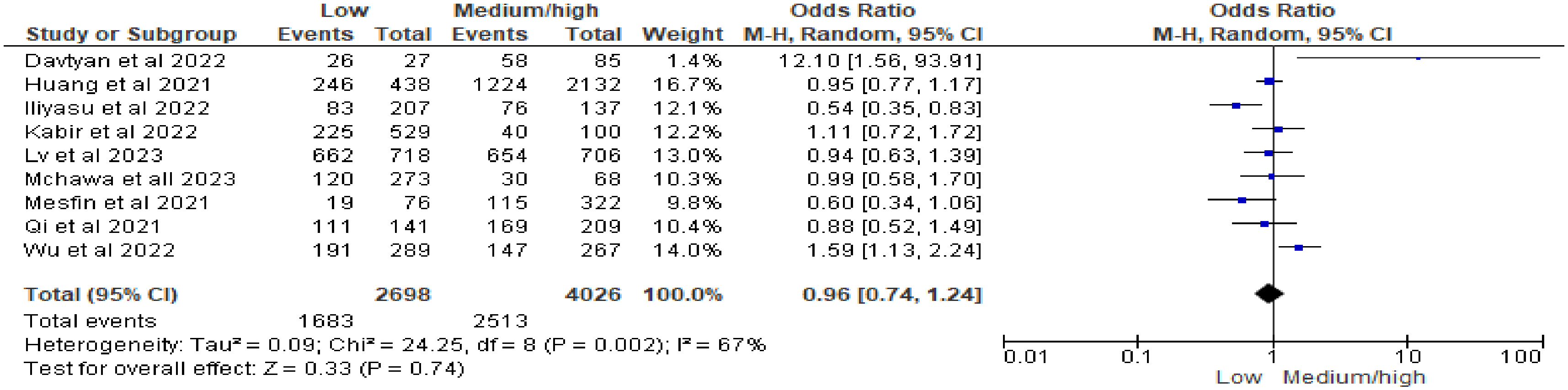

Supplementary Figure 47: Forest plots of random-effects model meta-analysis of income class as a determinant of COVID-19 vaccine acceptance pooled using the Mantel-Haenszel (M-H) method (n = 9 studies; N = 6,724 participants). Each blue-colored solid square represents the effect size (odds ratio, OR) of each characteristic, while the ends of the adjoining horizontal lines represent lower (left) and upper (right) confidence intervals. The isolated black-colored solid rhombus at the bottom denotes the overall estimated effect size and the 95% CI. All statistics were based on a two-sided t-test. df: degree of freedom.

Study or Subgroup	Rural Events	Rural Total	Urban Events	Urban Total	Weight	Odds Ratio M-H, Fixed, 95% CI
Kabir et al 2022	119	275	156	377	73.3%	1.08 [0.79, 1.48]
Mchawa et all 2023	140	326	10	15	10.7%	0.38 [0.13, 1.13]
Ortiz-Mertinez et al 2022	433	496	27	32	6.3%	1.27 [0.47, 3.43]
Tunta et al 2023	177	215	43	117	9.7%	8.02 [4.80, 13.40]
**Total (95% CI)**		**1312**		**541**	**100.0%**	**1.69 [1.33, 2.14]**
Total events	869		236			

Heterogeneity: Chi² = 50.55, df = 3 (P < 0.00001); I² = 94%

Test for overall effect: Z = 4.29 (P < 0.0001)

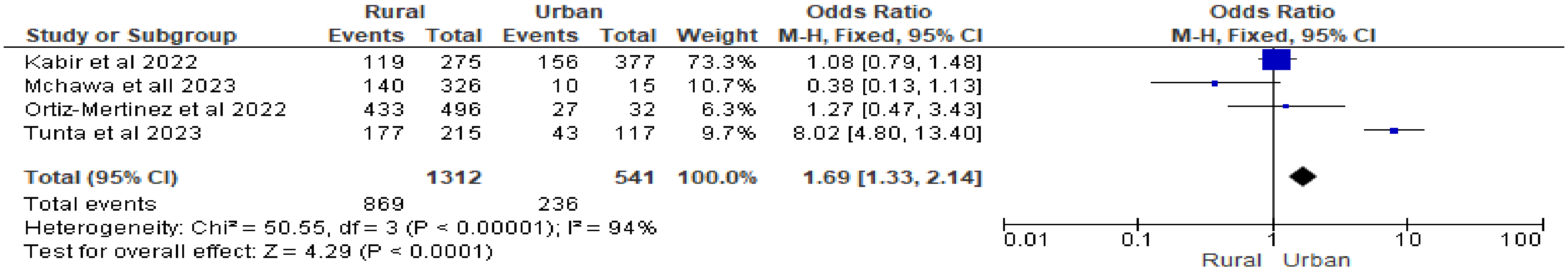

Supplementary Figure 48: Forest plots of fixed-effects model meta-analysis of residence as a determinant of COVID-19 vaccine acceptance pooled using the Mantel-Haenszel (M-H) method (n = 4 studies; N = 1,853 participants). Each blue-colored solid square represents the effect size (odds ratio, OR) of each characteristic, while the ends of the adjoining horizontal lines represent lower (left) and upper (right) confidence intervals. The isolated black-colored solid rhombus at the bottom denotes the overall estimated effect size and the 95% CI. All statistics were based on a two-sided t-test. df: degree of freedom.

Study or Subgroup	No Events	No Total	Yes Events	Yes Total	Weight	Odds Ratio M-H, Random, 95% CI
Huang et al 2021	1009	1707	461	863	12.5%	1.26 [1.07, 1.49]
Iliyasu et al 2022	104	250	55	94	11.3%	0.51 [0.31, 0.82]
Lv et al 2023	983	1048	333	376	11.7%	1.95 [1.30, 2.93]
Lyons et al 2023	118	192	42	73	11.0%	1.18 [0.68, 2.04]
Mesfin et al 2021	56	267	78	131	11.4%	0.18 [0.11, 0.28]
Ortiz-Mertinez et al 2022	404	461	56	67	10.1%	1.39 [0.69, 2.81]
Qi et al 2021	228	282	52	68	10.5%	1.30 [0.69, 2.45]
Vallee et al 2021	109	155	60	82	10.7%	0.87 [0.48, 1.58]
Wu et al 2022	279	481	59	75	10.8%	0.37 [0.21, 0.67]
**Total (95% CI)**		**4843**		**1829**	**100.0%**	**0.81 [0.49, 1.34]**
Total events	3290		1196			

Heterogeneity: Tau² = 0.51; Chi² = 93.03, df = 8 (P < 0.00001); I² = 91%

Test for overall effect: Z = 0.82 (P = 0.41)

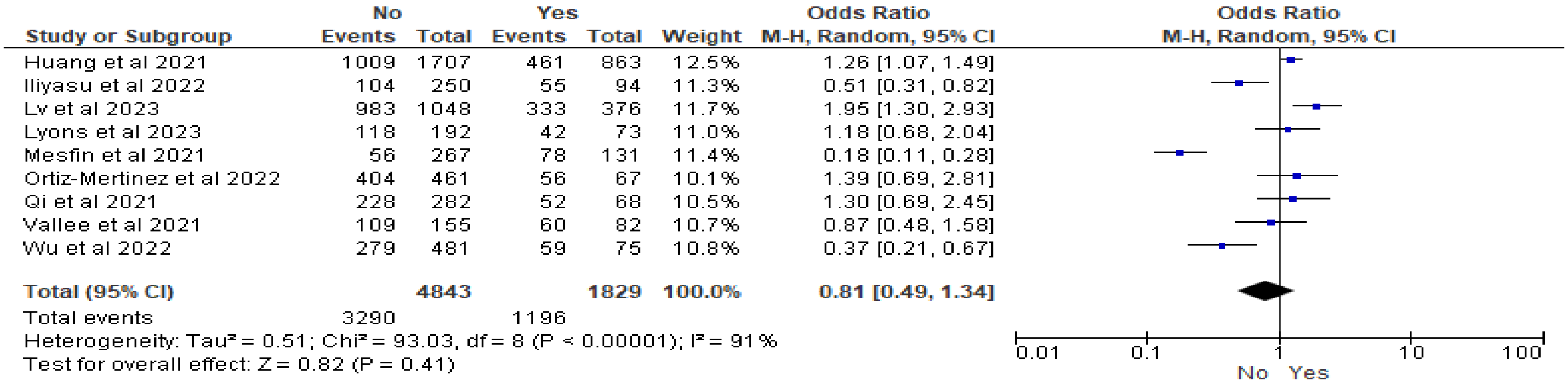

Supplementary Figure 49: Forest plots of random-effects model meta-analysis of comorbidity as a determinant of COVID-19 vaccine acceptance pooled using the Mantel-Haenszel (M-H) method (n = 9 studies; N = 6,672 participants). Each blue-colored solid square represents the effect size (odds ratio, OR) of each characteristic, while the ends of the adjoining horizontal lines represent lower (left) and upper (right) confidence intervals. The isolated black-colored solid rhombus at the bottom denotes the overall estimated effect size and the 95% CI. All statistics were based on a two-sided t-test. df: degree of freedom.

Study or Subgroup	Yes Events	Yes Total	No Events	No Total	Weight	Odds Ratio M-H, Fixed, 95% CI
Ekstrand et al 2021	41	118	229	320	42.8%	0.21 [0.13, 0.33]
Iliyasu et al 2022	98	227	61	117	24.3%	0.70 [0.45, 1.09]
Su et al 2022	567	711	5	20	1.0%	11.81 [4.22, 33.04]
Vallee et al 2021	35	88	134	149	31.9%	0.07 [0.04, 0.15]
**Total (95% CI)**		**1144**		**606**	**100.0%**	**0.41 [0.32, 0.53]**
Total events	741		429			

Heterogeneity: $Chi^2 = 78.81$, $df = 3$ ($P < 0.00001$); $I^2 = 96\%$
Test for overall effect: $Z = 6.89$ ($P < 0.00001$)

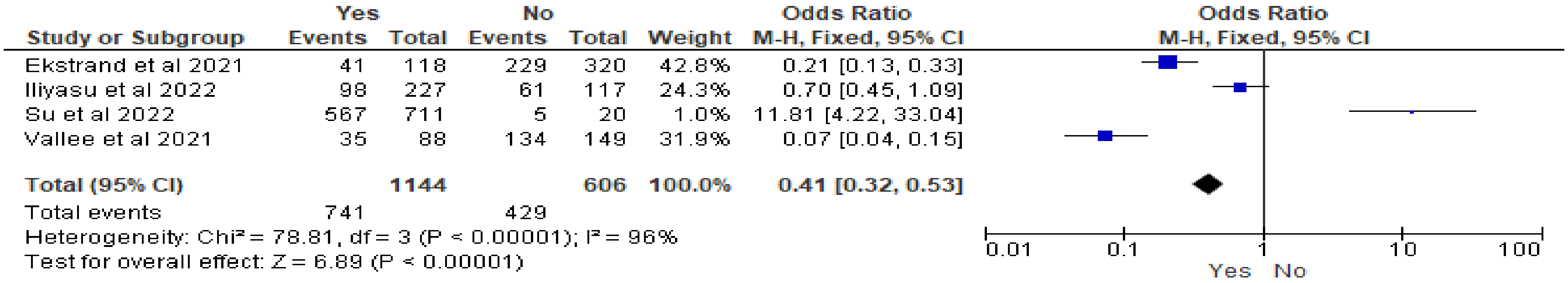

Supplementary Figure 50: Forest plots of fixed-effects model meta-analysis of concern about vaccine safety as a determinant of COVID-19 vaccine acceptance pooled using the Mantel-Haenszel (M-H) method (n = 4 studies; N = 1750 participants). Each blue-colored solid square represents the effect size (odds ratio, OR) of each characteristic, while the ends of the adjoining horizontal lines represent lower (left) and upper (right) confidence intervals. The isolated black-colored solid rhombus at the bottom denotes the overall estimated effect size and the 95% CI. All statistics were based on a two-sided t-test. df: degree of freedom.

Study or Subgroup	Yes Events	Yes Total	No Events	No Total	Weight	Odds Ratio M-H, Fixed, 95% CI
Iliyasu et al 2022	100	219	59	125	88.9%	0.94 [0.61, 1.46]
Su et al 2022	181	221	6	22	4.3%	12.07 [4.44, 32.76]
Vallee et al 2021	162	215	7	22	6.8%	6.55 [2.54, 16.92]
**Total (95% CI)**		**655**		**169**	**100.0%**	**1.80 [1.27, 2.56]**
Total events	443		72			
Heterogeneity: Chi² = 29.41, df = 2 (P < 0.00001); I² = 93%						
Test for overall effect: Z = 3.27 (P = 0.001)						

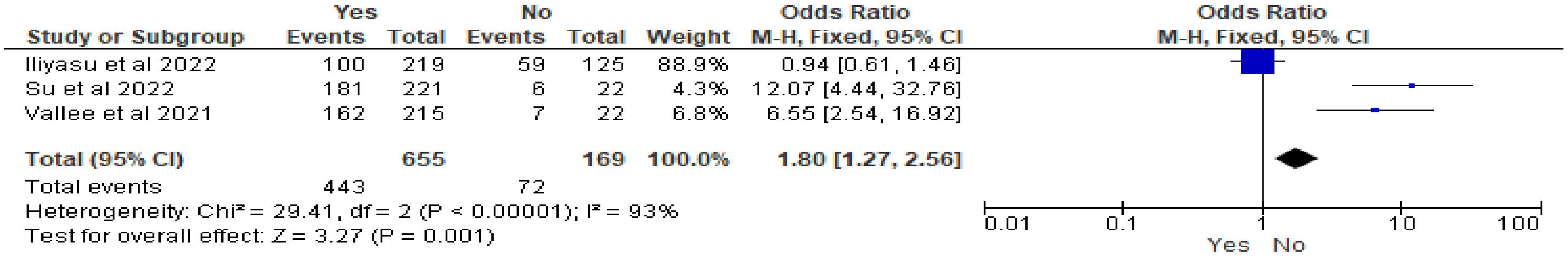

Supplementary Figure 51: Forest plots of fixed-effects model meta-analysis of perceived vaccine effectiveness as a determinant of COVID-19 vaccine acceptance pooled using the Mantel-Haenszel (M-H) method (n = 3 studies; N = 824 participants). Each blue-colored solid square represents the effect size (odds ratio, OR) of each characteristic, while the ends of the adjoining horizontal lines represent lower (left) and upper (right) confidence intervals. The isolated black-colored solid rhombus at the bottom denotes the overall estimated effect size and the 95% CI. All statistics were based on a two-sided t-test. df: degree of freedom.

Study or Subgroup	Yes Events	Yes Total	No Events	No Total	Weight	Odds Ratio M-H, Fixed, 95% CI
Ekstrand et al 2021	469	657	5	22	32.3%	8.48 [3.09, 23.32]
Lyons et al 2023	116	127	44	139	42.5%	22.77 [11.15, 46.51]
Vallee et al 2021	164	215	5	22	25.1%	10.93 [3.84, 31.10]
**Total (95% CI)**		**999**		**183**	**100.0%**	**15.17 [9.16, 25.12]**
Total events	749		54			
Heterogeneity: Chi² = 2.89, df = 2 (P = 0.24); I² = 31%						
Test for overall effect: Z = 10.57 (P < 0.00001)						

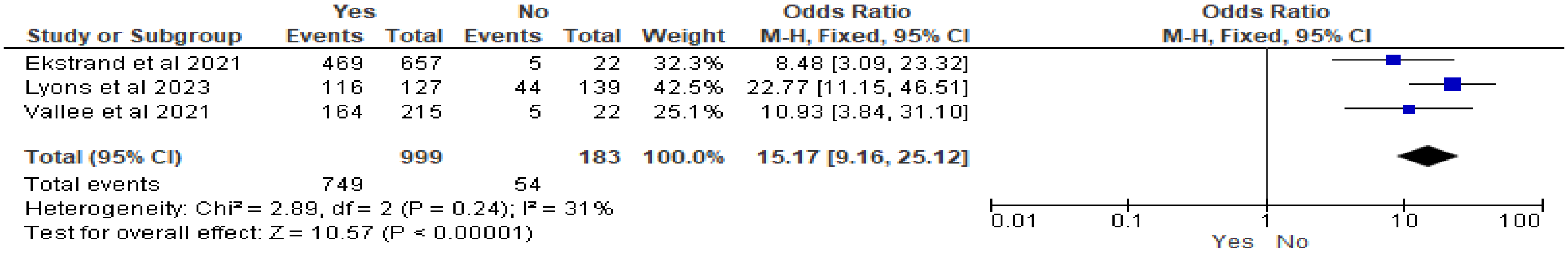

Supplementary Figure 52: Forest plots of fixed-effects model meta-analysis of vaccine trust as a determinant of COVID-19 vaccine acceptance pooled using the Mantel-Haenszel (M-H) method (n = 3 studies; N = 1,182 participants). Each blue-colored solid square represents the effect size (odds ratio, OR) of each characteristic, while the ends of the adjoining horizontal lines represent lower (left) and upper (right) confidence intervals. The isolated black-colored solid rhombus at the bottom denotes the overall estimated effect size and the 95% CI. All statistics were based on a two-sided t-test. df: degree of freedom.

2. Perceived susceptibility

Study or Subgroup	Yes Events	Yes Total	No Events	No Total	Weight	Odds Ratio M-H, Fixed, 95% CI
Kabir et al 2022	104	209	173	441	42.6%	1.53 [1.10, 2.14]
Vallee et al 2021	129	207	209	349	44.7%	1.11 [0.78, 1.58]
Wu et al 2022	59	77	110	160	12.7%	1.49 [0.80, 2.78]
**Total (95% CI)**		**493**		**950**	**100.0%**	**1.34 [1.07, 1.68]**
Total events	292		492			

Heterogeneity: $Chi^2 = 1.86$, df = 2 (P = 0.39); $I^2 = 0\%$
Test for overall effect: Z = 2.53 (P = 0.01)

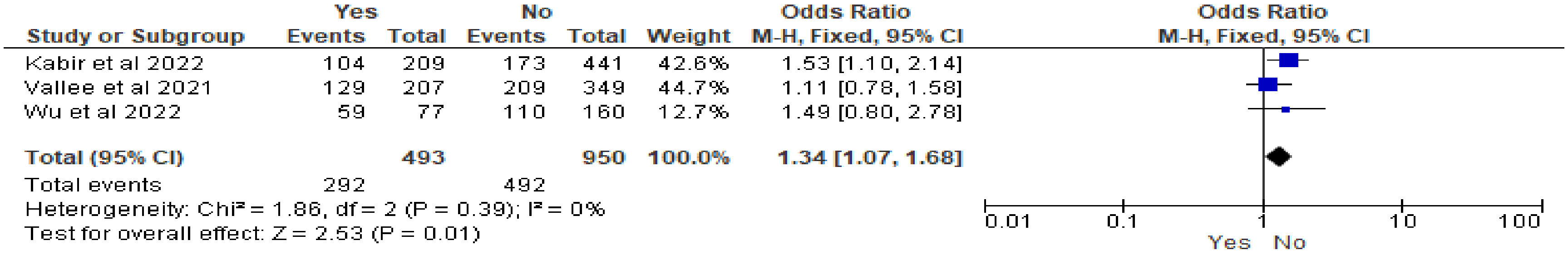

Supplementary Figure 53: Forest plots of fixed-effects model meta-analysis of perceived susceptibility to SARS-Cov-2 as a determinant of COVID-19 vaccine acceptance pooled using the Mantel-Haenszel (M-H) method (n = 3 studies; N = 1443 participants). Each blue-colored solid square represents the effect size (odds ratio, OR) of each characteristic, while the ends of the adjoining horizontal lines represent lower (left) and upper (right) confidence intervals. The isolated black-colored solid rhombus at the bottom denotes the overall estimated effect size and the 95% CI. All statistics were based on a two-sided t-test. df: degree of freedom.

Study or Subgroup	Yes Events	Yes Total	No Events	No Total	Weight	Odds Ratio M-H, Fixed, 95% CI
Govere-Hwenje et al 2022	62	117	54	90	29.0%	0.75 [0.43, 1.31]
Iliyasu et al 2022	100	149	59	195	17.0%	4.70 [2.97, 7.44]
Vallee et al 2021	123	164	34	61	12.5%	2.38 [1.29, 4.41]
Wu et al 2022	259	403	79	153	41.4%	1.68 [1.16, 2.46]
**Total (95% CI)**		**833**		**499**	**100.0%**	**2.01 [1.60, 2.54]**
Total events	544		226			

Heterogeneity: $Chi^2 = 26.36$, df = 3 (P < 0.00001); $I^2 = 89\%$
Test for overall effect: Z = 5.91 (P < 0.00001)

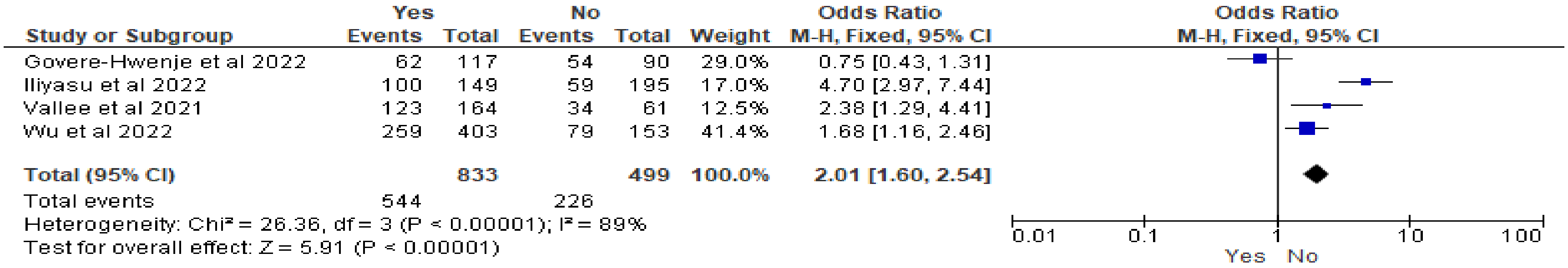

Supplementary Figure 54: Forest plots of random-effects model meta-analysis of fear of SARS-Cov-2 effect in PLHIV as a determinant of COVID-19 vaccine acceptance pooled using the Mantel-Haenszel (M-H) method (n = 4 studies; N = 1332 participants). Each blue-colored solid square represents the effect size (odds ratio, OR) of each characteristic, while the ends of

the adjoining horizontal lines represent lower (left) and upper (right) confidence intervals. The isolated black-colored solid rhombus at the bottom denotes the overall estimated effect size and the 95% CI. All statistics were based on a two-sided t-test. df: degree of freedom.

3. Receipt of influenza vaccination

Study or Subgroup	Yes Events	Yes Total	No Events	No Total	Weight	Odds Ratio M-H, Fixed, 95% CI
Govere-Hwenje et al 2022	23	32	98	180	3.7%	2.14 [0.94, 4.88]
Huang et al 2021	360	568	1110	2002	80.8%	1.39 [1.15, 1.69]
Ortiz-Mertinez et al 2022	326	365	134	163	8.9%	1.81 [1.07, 3.05]
Vallee et al 2021	100	125	69	112	6.5%	2.49 [1.39, 4.45]
**Total (95% CI)**		**1090**		**2457**	**100.0%**	**1.53 [1.29, 1.81]**
Total events	809		1411			

Heterogeneity: $Chi^2 = 4.69$, $df = 3$ ($P = 0.20$); $I^2 = 36\%$
Test for overall effect: $Z = 4.93$ ($P < 0.00001$)

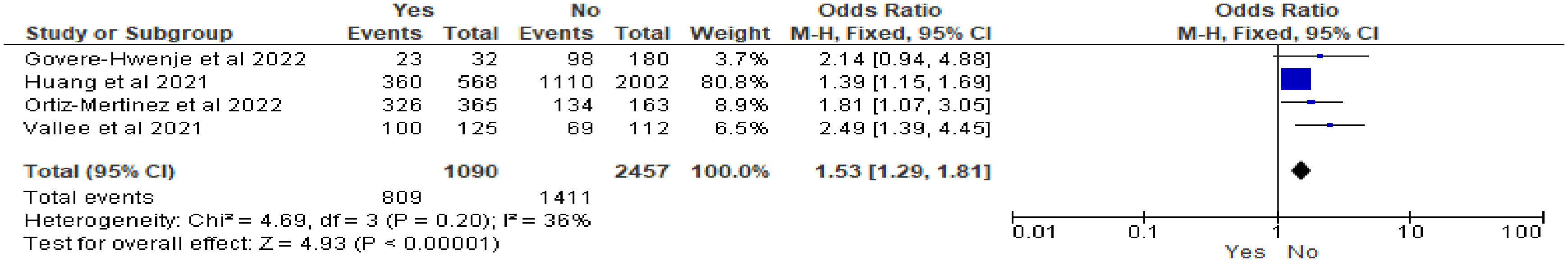

Supplementary Figure 55: Forest plots of random-effects model meta-analysis of recent influenza vaccination as a determinant of COVID-19 vaccine acceptance pooled using the Mantel-Haenszel (M-H) method (n = 4 studies; N = 3,547 participants). Each blue-colored solid square represents the effect size (odds ratio, OR) of each characteristic, while the ends of the adjoining horizontal lines represent lower (left) and upper (right) confidence intervals. The isolated black-colored solid rhombus at the bottom denotes the overall estimated effect size and the 95% CI. All statistics were based on a two-sided t-test. df: degree of freedom.

Study or Subgroup	Yes Events	Yes Total	No Events	No Total	Weight	Odds Ratio M-H, Fixed, 95% CI
Ekstrand et al 2021	36	49	234	389	37.1%	1.83 [0.94, 3.57]
Vallee et al 2021	36	57	133	180	62.9%	0.61 [0.32, 1.14]
**Total (95% CI)**		**106**		**569**	**100.0%**	**1.06 [0.68, 1.66]**
Total events	72		367			

Heterogeneity: Chi² = 5.61, df = 1 (P = 0.02); I² = 82%
Test for overall effect: Z = 0.26 (P = 0.79)

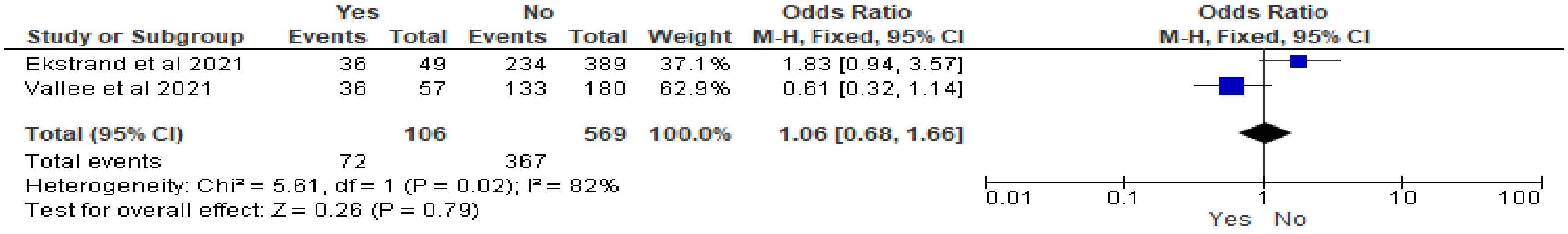

Supplementary Figure 56: Forest plots of random-effects model meta-analysis of knowing someone who died of COVID-19 as a determinant of COVID-19 vaccine acceptance pooled using the Mantel-Haenszel (M-H) method (n = 2 studies; N = 675 participants). Each blue-colored solid square represents the effect size (odds ratio, OR) of each characteristic, while the ends of the adjoining horizontal lines represent lower (left) and upper (right) confidence intervals. The isolated black-colored solid rhombus at the bottom denotes the overall estimated effect size and the 95% CI. All statistics were based on a two-sided t-test. df: degree of freedom.

SECTION E

Results of the meta-analysis of the determinants of COVID-19 vaccine uptake in PLHIV

Study or Subgroup	Men Events	Men Total	Women Events	Women Total	Weight	Odds Ratio M-H, Random, 95% CI
Al-Yammahi et al 2021	20	39	19	39	4.7%	1.11 [0.46, 2.69]
Constiniuk et all 2023	162	189	57	69	6.6%	1.26 [0.60, 2.66]
Jaiswal et al 2022	252	355	67	128	17.6%	2.23 [1.47, 3.38]
Menza et al 2022	4460	7095	483	927	54.9%	1.56 [1.36, 1.79]
Qi et al 2021	149	335	4	15	2.8%	2.20 [0.69, 7.06]
Zhao et al 2021	503	1541	24	77	13.4%	1.07 [0.65, 1.75]
**Total (95% CI)**		**9554**		**1255**	**100.0%**	**1.55 [1.27, 1.89]**
Total events	5546		654			

Heterogeneity: Tau² = 0.01; Chi² = 6.27, df = 5 (P = 0.28); I² = 20%
Test for overall effect: Z = 4.27 (P < 0.0001)

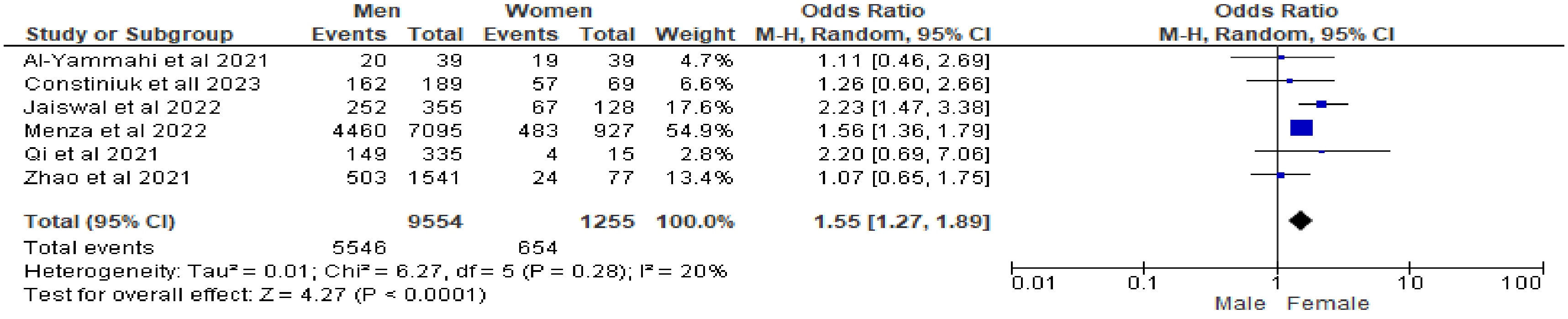

Supplementary Figure 57: Forest plots of random-effects model meta-analysis of gender as a determinant of COVID-19 vaccine uptake pooled using the Mantel-Haenszel (M-H) method (n = 6 studies; N = 10,809 participants). Each blue-colored solid square

represents the effect size (odds ratio, OR) of each characteristic, while the ends of the adjoining horizontal lines represent lower (left) and upper (right) confidence intervals. The isolated black-colored solid rhombus at the bottom denotes the overall estimated effect size and the 95% CI. All statistics were based on a two-sided t-test. df: degree of freedom.

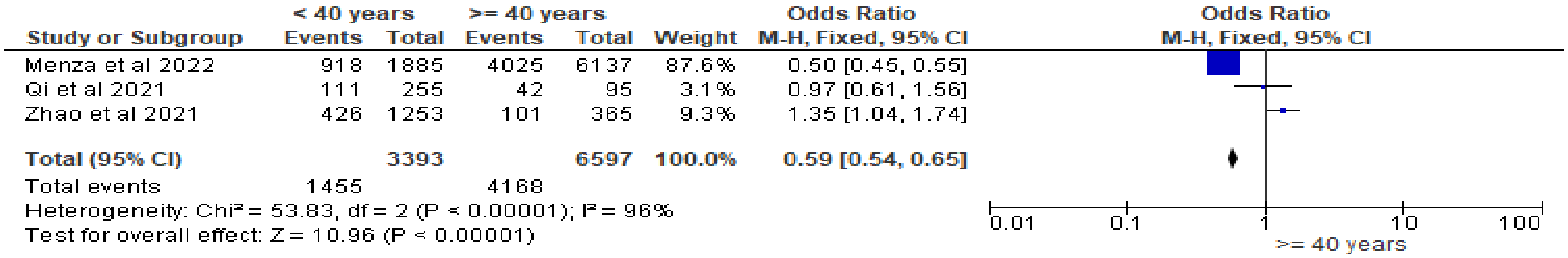

Supplementary Figure 58: Forest plots of fixed-effects model meta-analysis of age as a determinant of COVID-19 vaccine uptake pooled using the Mantel-Haenszel (M-H) method (n = 3 studies; N = 9,990 participants). Each blue-colored solid square represents the effect size (odds ratio, OR) of each characteristic, while the ends of the adjoining horizontal lines represent lower (left) and upper (right) confidence intervals. The isolated black-colored solid rhombus at the bottom denotes the overall estimated effect size and the 95% CI. All statistics were based on a two-sided t-test. df: degree of freedom.

Study or Subgroup	Single/divorced/widowed Events	Total	Married/cohabited Events	Total	Weight	Odds Ratio M-H, Fixed, 95% CI
Qi et al 2021	121	268	32	82	18.1%	1.29 [0.78, 2.13]
Zhao et al 2021	419	1330	108	288	81.9%	0.77 [0.59, 1.00]
**Total (95% CI)**		**1598**		**370**	**100.0%**	**0.86 [0.68, 1.09]**
Total events	540		140			
Heterogeneity: Chi² = 3.17, df = 1 (P = 0.08); I² = 68%						
Test for overall effect: Z = 1.25 (P = 0.21)						

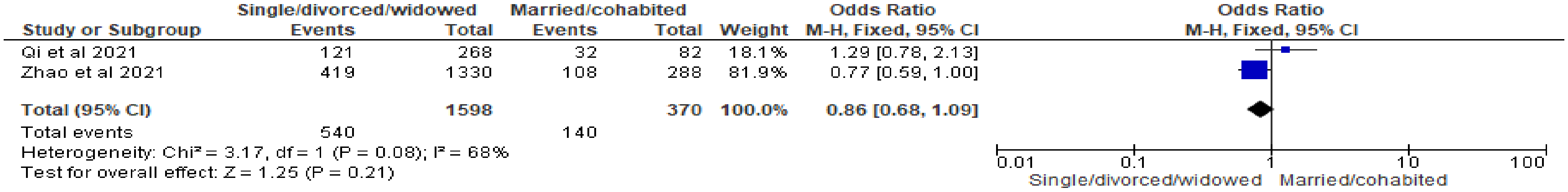

Supplementary Figure 59: Forest plots of fixed-effects model meta-analysis of marital status as a determinant of COVID-19 vaccine uptake pooled using the Mantel-Haenszel (M-H) method (n = 2 studies; N = 1,968 participants). Each blue-colored solid square represents the effect size (odds ratio, OR) of each characteristic, while the ends of the adjoining horizontal lines represent lower (left) and upper (right) confidence intervals. The isolated black-colored solid rhombus at the bottom denotes the overall estimated effect size and the 95% CI. All statistics were based on a two-sided t-test. df: degree of freedom.

Study or Subgroup	Black Events	Total	White Events	Total	Weight	Odds Ratio M-H, Fixed, 95% CI
Constiniuk et all 2023	100	115	115	139	3.2%	1.39 [0.69, 2.80]
Jaiswal et al 2022	75	129	167	233	11.9%	0.55 [0.35, 0.86]
Menza et al 2022	330	631	3782	5781	84.8%	0.58 [0.49, 0.68]
**Total (95% CI)**		**875**		**6153**	**100.0%**	**0.60 [0.52, 0.70]**
Total events	505		4064			
Heterogeneity: Chi² = 5.89, df = 2 (P = 0.05); I² = 66%						
Test for overall effect: Z = 6.57 (P < 0.00001)						

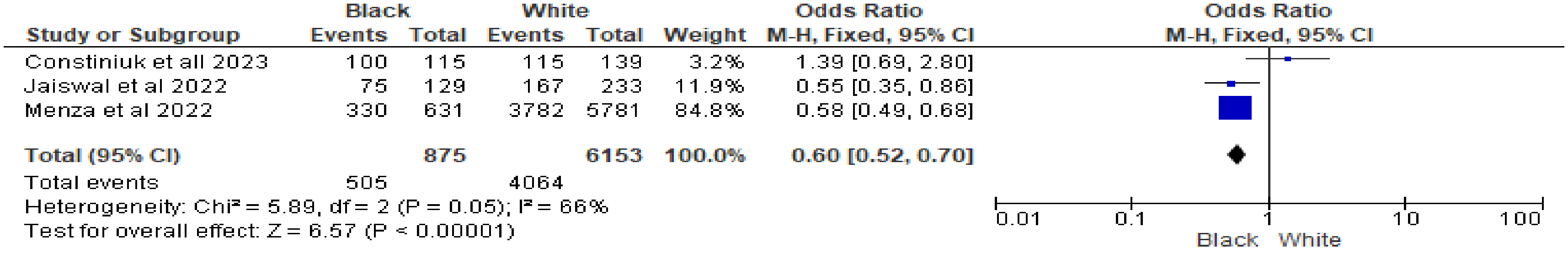

Supplementary Figure 60: Forest plots of fixed-effects model meta-analysis of race (Black vs. White) as a determinant of COVID-19 vaccine uptake pooled using the Mantel-Haenszel (M-H) method (n = 3 studies; N = 7,028 participants). Each blue-colored solid square represents the effect size (odds ratio, OR) of each characteristic, while the ends of the adjoining horizontal lines represent lower (left) and upper (right) confidence intervals. The isolated black-colored solid rhombus at the bottom denotes the overall estimated effect size and the 95% CI. All statistics were based on a two-sided t-test. df: degree of freedom.

Study or Subgroup	Others Events	Others Total	White Events	White Total	Weight	Odds Ratio M-H, Fixed, 95% CI
Jaiswal et al 2022	77	122	167	233	3.0%	0.68 [0.42, 1.08]
Menza et al 2022	825	2282	3782	5781	97.0%	0.30 [0.27, 0.33]
**Total (95% CI)**		**2404**		**6014**	**100.0%**	**0.31 [0.28, 0.34]**
Total events	902		3949			

Heterogeneity: Chi² = 11.26, df = 1 (P = 0.0008); I² = 91%
Test for overall effect: Z = 23.24 (P < 0.00001)

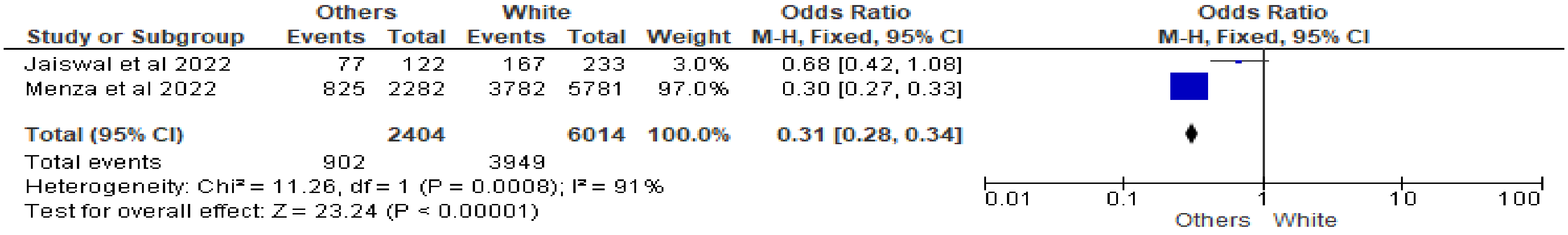

Supplementary Figure 61: Forest plots of fixed-effects model meta-analysis of Race (Others [Latinx/Hispanic/Mixed race] vs White people) as a determinant of COVID-19 vaccine uptake pooled using the Mantel-Haenszel (M-H) method (n = 2 studies; N = 8,418 participants). Each blue-colored solid square represents the effect size (odds ratio, OR) of each characteristic, while the ends of the adjoining horizontal lines represent lower (left) and upper (right) confidence intervals. The isolated black-colored solid rhombus at the bottom denotes the overall estimated effect size and the 95% CI. All statistics were based on a two-sided t-test. df: degree of freedom.

Study or Subgroup	Primary and below Events	Primary and below Total	Secondary and above Events	Secondary and above Total	Weight	Odds Ratio M-H, Fixed, 95% CI
Constiniuk et all 2023	4	5	214	252	0.6%	0.71 [0.08, 6.53]
Jaiswal et al 2022	52	102	267	383	20.3%	0.45 [0.29, 0.71]
Lv et al 2023	224	273	1034	1151	26.3%	0.52 [0.36, 0.74]
Qi et al 2021	36	87	117	263	12.6%	0.88 [0.54, 1.44]
Zhao et al 2021	37	217	490	1401	40.2%	0.38 [0.26, 0.55]
**Total (95% CI)**		**684**		**3450**	**100.0%**	**0.50 [0.41, 0.61]**
Total events	353		2122			

Heterogeneity: Chi² = 7.47, df = 4 (P = 0.11); I² = 46%
Test for overall effect: Z = 6.80 (P < 0.00001)

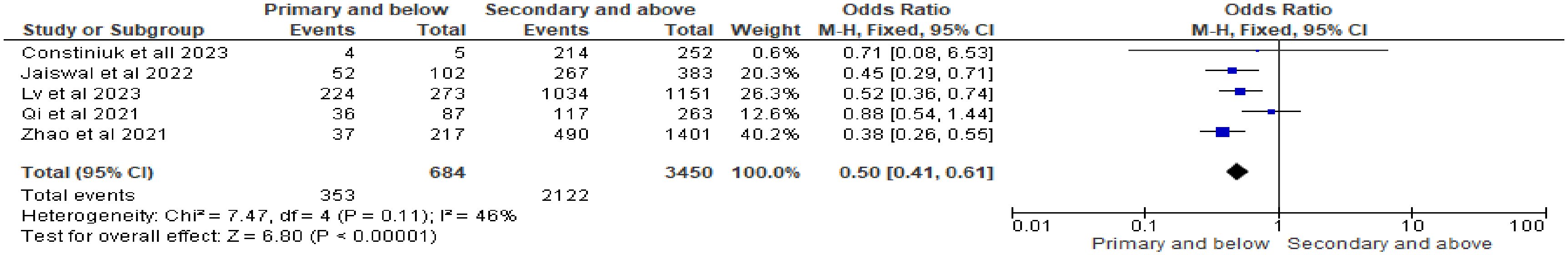

Supplementary Figure 61: Forest plots of fixed-effects model meta-analysis of educational level as a determinant of COVID-19 vaccine uptake pooled using the Mantel-Haenszel (M-H) method (n = 5 studies; N = 4,134 participants). Each blue-colored solid square represents the effect size (odds ratio, OR) of each characteristic, while the ends of the adjoining horizontal lines represent lower (left) and upper (right) confidence intervals. The isolated black-colored solid rhombus at the bottom denotes the overall estimated effect size and the 95% CI. All statistics were based on a two-sided t-test. df: degree of freedom.

Study or Subgroup	Unemployed Events	Unemployed Total	Employed Events	Employed Total	Weight	Odds Ratio M-H, Fixed, 95% CI
Al-Yammahi et al 2021	16	40	24	40	9.0%	0.44 [0.18, 1.09]
Constiniuk et all 2023	95	139	120	139	23.9%	0.34 [0.19, 0.62]
Jaiswal et al 2022	140	216	179	268	35.3%	0.92 [0.63, 1.34]
Zhao et al 2021	15	95	512	1523	31.8%	0.37 [0.21, 0.65]
**Total (95% CI)**		**490**		**1970**	**100.0%**	**0.56 [0.43, 0.73]**
Total events	266		835			

Heterogeneity: Chi² = 11.43, df = 3 (P = 0.010); I² = 74%
Test for overall effect: Z = 4.36 (P < 0.0001)

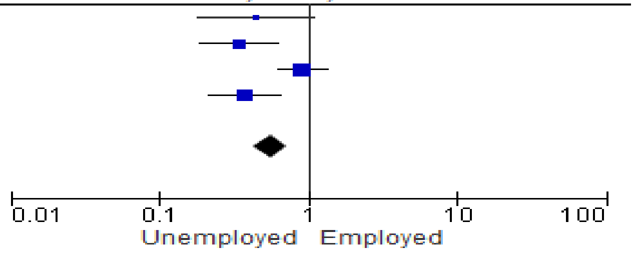

Supplementary Figure 62: Forest plots of fixed-effects model meta-analysis of employment status as a determinant of COVID-19 vaccine uptake pooled using the Mantel-Haenszel (M-H) method (n = 4 studies; N = 2,460 participants). Each blue-colored solid square represents the effect size (odds ratio, OR) of each characteristic, while the ends of the adjoining horizontal lines represent lower (left) and upper (right) confidence intervals. The isolated black-colored solid rhombus at the bottom denotes the overall estimated effect size and the 95% CI. All statistics were based on a two-sided t-test. df: degree of freedom.

Study or Subgroup	Yes Events	Yes Total	No Events	No Total	Weight	Odds Ratio M-H, Fixed, 95% CI
Menza et al 2022	3956	5065	987	2957	88.7%	7.12 [6.43, 7.88]
Zhao et al 2021	101	167	426	1451	11.3%	3.68 [2.65, 5.12]
**Total (95% CI)**		**5232**		**4408**	**100.0%**	**6.73 [6.11, 7.41]**
Total events	4057		1413			

Heterogeneity: Chi² = 14.00, df = 1 (P = 0.0002); I² = 93%
Test for overall effect: Z = 38.66 (P < 0.00001)

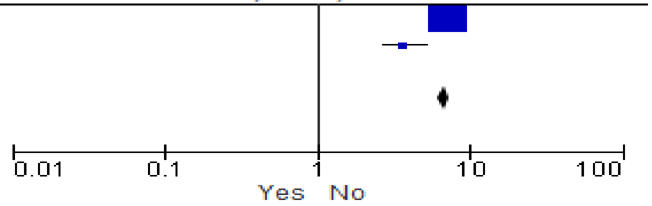

Supplementary Figure 63: Forest plots of fixed-effects model meta-analysis of influenza vaccination as a determinant of COVID-19 vaccine uptake pooled using the Mantel-Haenszel (M-H) method (n = 2 studies; N = 9,640 participants). Each blue-colored solid square represents the effect size (odds ratio, OR) of each characteristic, while the ends of the adjoining horizontal lines represent lower (left) and upper (right) confidence intervals. The isolated black-colored solid rhombus at the bottom denotes the overall estimated effect size and the 95% CI. All statistics were based on a two-sided t-test. df: degree of freedom.

SECTION F

Table 2: The Preferred Reporting Item for Systematic Reviews and Meta-analysis (PRISMA) 2020 checklist of the present work

Section and Topic	Item #	Checklist item	Location where item is reported
**TITLE**			
Title	1	Identify the report as a literature review.	1
**ABSTRACT**			
Abstract	2	Provide a structured summary including, as applicable: background; objectives; data sources; study eligibility criteria, participants, and interventions; study appraisal and synthesis methods; results; limitations; conclusions and implications of key findings. See the PRISMA 2020 for Abstracts checklist for the complete list.	1
**INTRODUCTION**			
Rationale	3	Describe the rationale for the review in the context of existing knowledge, i.e., what is already known about your topic.	2, 3
Objectives	4	Provide an explicit statement of the objective(s) or question(s) the review addresses with reference to participants, interventions, comparisons, outcomes, and study design (PICOS).	3
**METHODS**			
Eligibility criteria	5	Specify the inclusion and exclusion criteria for the review and how studies were grouped for the syntheses with study characteristics (e.g., PICOS, length of follow-up) and report characteristics (e.g., years considered, language, publication status) used as criteria for	21

Section and Topic	Item #	Checklist item	Location where item is reported
		eligibility, giving rationale.	
Information sources	6	Specify all databases, registers, websites, organisations, reference lists and other sources searched or consulted to identify studies. Specify the date when each source was last searched or consulted.	20
Search strategy	7	Present the full search strategies for all databases, registers and websites, including any filters and limits used.	20
Selection process	8	State the process for selecting studies (i.e., screening, eligibility). Specify the methods used to decide whether a study met the inclusion criteria of the review, including how many reviewers screened each record and each report retrieved, whether they worked independently, and if applicable, details of automation tools used in the process.	20,21
Study risk of bias assessment	11	Specify the methods used to assess risk of bias in the included studies, including details of the tool(s) used, how many reviewers assessed each study and whether they worked independently, and if applicable, details of automation tools used in the process.	23
**RESULTS**			
Study selection	16a	Describe the results of the search and selection process, from the number of records identified in the search to the number of studies included in the review, ideally using a flow diagram.	6, 7
	16b	Cite studies that might appear to meet the inclusion criteria, but which were excluded, and explain why they were excluded.	6
Study characteristics	17	Cite each included study and present its characteristics (e.g., study size, PICOS, follow-up period).	7 - 9
Risk of bias in studies	18	Present assessments of risk of bias for each included study.	8,9
Results of individual studies	19	For all outcomes, present, for each study: (a) summary statistics for each group (where appropriate) and (b) an effect estimate and its precision (e.g. confidence/credible interval), ideally using structured tables or plots.	9 - 14

Section and Topic	Item #	Checklist item	Location where item is reported
**DISCUSSION**			
Discussion	23a	Provide a general interpretation of the results in the context of other evidence.	14 - 19
	23b	Discuss any limitations of the evidence included in the review.	19
	23c	Discuss any limitations of the review processes used.	19
	23d	Discuss implications of the results for practice, policy, and future research.	20
**OTHER INFORMATION**			
Registration and protocol	24a	Provide registration information for the review, including register name and registration number, or state that the review was not registered.	21
	24b	Indicate where the review protocol can be accessed, or state that a protocol was not prepared.	20
	24c	Describe and explain any amendments to information provided at registration or in the protocol.	21 to 25
Support	25	Describe sources of financial or non-financial support for the review, and the role of the funders or sponsors in the review.	26
Competing interests	26	Declare any competing interests of review authors.	26
Availability of data, code, and other materials	27	Report which of the following are publicly available and where they can be found: template data collection forms; data extracted from included studies; data used for all analyses; analytic code; any other materials used in the review.	26

Reference source: Page MJ, McKenzie JE, Bossuyt PM, Boutron I, Hoffmann TC, Mulrow CD, et al. The PRISMA 2020 statement: an updated guideline for reporting systematic reviews. BMJ 2021;372:n71. doi: 10.1136/bmj.n71

SECTION G

Table 3. The literature search strategy used across all databases used

**PUBMED**<br><br>Key concepts<br><br>**Concept 1: COVID-19**<br><br>("COVID-19"[Mesh] OR "SARS-CoV-2"[Mesh] OR covid* OR coronavirus* OR 2019-ncov OR sars-cov-2 OR cov-19)<br><br>**Concept 2: Vaccine**<br><br>("Vaccines"[Mesh] OR "COVID-19 Vaccines"[Mesh] OR vaccin* OR immuniz*)<br><br>**Concept 3: Hesitancy, Uptake, Refusal, Acceptance**<br><br>("Vaccination Hesitancy"[Mesh] OR "Vaccination Refusal"[Mesh] OR hesitan* OR uptake OR accept* OR refus* OR declin* OR intension* OR intend* OR intent* OR willing*)<br><br>**Concepts 4: Determinants**<br><br>(determinant* OR factor* OR cause* OR predictor* OR "risk factor*")<br><br>**Concept 5: PLHIV**<br><br>**("HIV"[Mesh] OR** "people living with hiv" OR "patients living with hiv" OR plwh OR plwha OR plhiv)<br><br>**Combination**<br><br>**1 AND 2 AND (3OR 4) AND 5**
**COCHRANE**<br><br>Cochrane Database of Systematic Reviews, Cochrane Central Register of Controlled Trials, APA PsycInfo, CINAHL – (covid* OR coronavirus* OR 2019-ncov OR sars-cov-2 OR cov-19) AND (vaccin* OR immuniz*) AND (hesitan* OR uptake OR

accept* OR refus* OR declin* OR determinant* OR factor* OR cause* OR predictor* OR "risk factor*" OR intension* OR intend* OR intent* OR willing*) AND ("people living with hiv" OR "patients living with hiv" OR plwh OR plwha OR plhiv)
**SCOPUS**<br><br>TITLE-ABS-KEY (covid* OR coronavirus* OR 2019-ncov OR sars-cov-2 OR cov-19) AND TITLE-ABS-KEY (vaccin* OR immuniz*) AND TITLE-ABS-KEY (hesitan* OR uptake OR accept* OR refus* OR declin* OR determinant* OR factor* OR cause* OR predictor* OR "risk factor*" OR intension* OR intend* OR intent* OR willing*) AND TITLE-ABS-KEY ("people living with hiv" OR "patients living with hiv" OR plwh OR plwha OR plhiv)
**GOOGLE SCHOLAR**<br><br>**EMBASE**<br><br>**Concept 1:**<br><br>("severe acute respiratory syndrome coronavirus 2*" OR ("SARS-CoV-2*" NEAR/3 "corona*") OR "coronavirus disease 2019*") OR "COVID-19*") OR "2019-ncov*")kw.<br><br>**Concept 2:**<br><br>("vaccin*" OR "vaccination*" OR "immunization*" OR "immunisation*" OR "vaccination hesitancy*" OR "reluctance*" OR "resistance*" OR "refusal*" OR "acceptance*" OR "willingness*" OR "behavi*" OR "uptake*" OR "coverage*" OR "barriers*" OR "factor*" OR "opinion*" OR "determinant*" OR respons* OR "predictor*" OR "intention*" OR "intention*" OR "decline*" OR "decision*")kw<br><br>**Concept 3:** 1 AND 2
**WEB OF SCIENCE**<br><br>**Concept 1:**<br><br>TI= ("(severe acute respiratory syndrome coronavirus 2*" OR ("SARS-CoV-2*" NEAR/3 "corona*") OR "coronavirus disease 2019*") OR "COVID-19*") OR "2019-ncov*") AB= ("severe acute respiratory syndrome coronavirus 2*" OR ("SARS-CoV-2*" NEAR/3 "corona *") OR "coronavirus disease 2019*") OR "COVID-19*") OR "2019-ncov*") OR AK= ("severe acute respiratory

syndrome coronavirus 2*” OR (“SARS-CoV-2*” NEAR/3 “corona *”) OR “coronavirus disease 2019*”) OR “COVID-19*”) OR ”2019-ncov*”).

Concept 2:

TI=(“vaccin*” OR “vaccination*” OR “immunization*” OR “immunisation*” OR “vaccination hesitancy*” OR “reluctance*” OR “resistance*” OR “refusal*” OR “acceptance*” OR “willingness*” OR “behavi*” OR “uptake*” OR “coverage*” OR “barriers*” OR “factor*” OR “opinion*” OR “determinant*” OR respons* OR “predictor*” OR “intention*” OR “intention*” OR “decline*” OR “decision*”) OR AB= (“vaccin*” OR “vaccination*” OR “immunization*” OR “immunisation*” OR “vaccination hesitancy*” OR “reluctance*” OR “resistance*” OR “refusal*” OR “acceptance*” OR “willingness*” OR “behavi*” OR “uptake*” OR “coverage*” OR “barriers*” OR “factor*” OR “opinion*” OR “determinant*” OR respons* OR “predictor*” OR “intention*” OR “intention*” OR “decline*” OR “decision*”) OR AK= =(“vaccin*” OR “vaccination*” OR “immunization*” OR “immunisation*” OR “vaccination hesitancy*” OR “reluctance*” OR “resistance*” OR “refusal*” OR “acceptance*” OR “willingness*” OR “behavi*” OR “uptake*” OR “coverage*” OR “barriers*” OR “factor*” OR “opinion*” OR “determinant*” OR respons* OR “predictor*” OR “intention*” OR “intention*” OR “decline*” OR “decision*”)

Concept 3:

T1= (“HIV*” OR "people living with hiv*" OR "patients living with hiv*" OR “plwh*” OR “plwha*” OR “plhiv*”) OR AB= (“HIV*” OR "people living with hiv*" OR "patients living with hiv*" OR “plwh*” OR “plwha*” OR “plhiv*”) OR AK= (“HIV*” OR "people living with hiv*" OR "patients living with hiv*" OR “plwh*” OR “plwha*” OR “plhiv*”)

Concept 4: 1 AND 2 AND 3

SECTION H

Quality assessment (critical appraisal) of all the included studies

Table 4. Risk of bias for included cross-sectional studies in the systematic review.

**Author Reference**	**Was the sample frame appropriate to address the target population?**	**Were study participants sampled in an appropriate way?**	**Was the sample size adequate?**	**Were the study subjects and the setting described in detail?**	**Was the data analysis conducted with sufficient coverage of the identified sample?**	**Were valid methods used for the identification of the condition?**	**Was the condition measured in a standard, reliable way for all participants?**	**Was there appropriate statistical analysis?**	**Was the response rate adequate, and if not, was the low response rate managed appropriately?**	**Total (%)**	**Quality level (High >50%, low <50%)**
Qi et al	1	1	1	1	1	1	1	1	NC	88.89	High
Liu	1	1	1	1	1	NC	1	1	1	88.89	High
Yang	1	1	1	1	1	1	1	1	1	100	High
Wu et al	1	1	1	1	1	NA	1	1	1	88.90	High
Vallée et al	1	1	1	0	1	NA		1	0	66.70	High
Shrestha et al	1	1	1	1	1	1	1	1	1	100	High
Menza et al	1	1	1	1	1	NA	1	1	1	88.90	High

O’Regan et al	1	0	0	1	1	NA	1	1	NC	55.56	High
Davtyan et al	1	1	1	1	1	NA	1	1	NC	77.78	High
Mesfin et al	1	1	1	1	1	1	1	1	1	100	High
Kaida et al	1	1		1	1	1	1	1	1	100	High
Su et al	1	1	1	1	1	1	1	1	1	100	High
Megan et al	1	1	1	0	1	0	1	1	1	77.78	High
Iliyasu et al	1	1	1	1	1	1	1	1	1	100	High
Chai et al	1	1	1	1	1	1	1	1	1	100	High
Jaiswa et al	1	1	1	1	1	1	1	1	NC	88.89	High
Jones et al	1	1	1	1	1	1	1	1	1	100	High
Zhao et al	1	1	1	1	1	NC	1	1	1	88.89	High
Zheng et al	1	1	1	1	1	1	1	1	1	100	High
Govere-Hwenje et al	1	1	NC	1	1	1	1	1	1	88.89	High
Huang et al	1	1	1	1	1	1	1	1	1	100	High

Ortiz-Mart´ınez et al	1	1	1	1	1	1	1	1	NC	88.89	High
Fulda et al	1	1	1	0	1	1	1	1	1	88.89	High
Mohammed et al	1	1	1	1	1	1	1	1	1	100	High
Kabir et al	1	1	1	1	1	1	1	1	1	100%	High
Donohue et al	1	1	0	0	1	1	1	1	0	66.67%	High
Holt et al	1	1	1	1	1	1	1	1	1	100%	High
Muhindo et al	1	1	1	1	1	1	1	1	1	100%	High
Swendeman et al	1	1	1	1	1	1	1	1	1	100%	High
Wickersham et al	1	1	1	0	1	1	1	1	1	88.89%	High
Prestage	1	1	1	1	1	1	1	1	1	100%	High
Folayan et al	1	1	1	1	1	1	1	1	1	100%	High
Bogart et al	1	1	0	1	1	1	1	1	1	88.89%	High
Lyons et al	1	1	NC	1	1	1	1	1	NC	77.78%	High

Madzima et al	1	1	1	1	1	1	1	1	1	100%	High
Chaudhuri et al	1	1	1	1	1	1	1	1	1	100%	High
Prestage	1	1	1	1	1	1	1	1	1	100%	High
Siewe Fodjo et al	1	1	1	1	1	1	1	1	NC	88.89%	High
Bert et al	1	1	NC	0	1	1	1	1	NC	66.67%	High
Shallangwa et al	1	1	1	1	1	1	1	1	1	100%	High
Costiniuk et al	1	1	1	1	1	1	1	1	1	100	High
Cummings et al	1	1	1	1	1	1	1	1	1	100	High
Singh et al	1	1	1	1	1	1	0	1	1	88.89	High
Berhie	1	1	0	0	1	0	1	1	1	66.67	High
LV et al	1	1	1	1	1	1	1	1	1	100	High
Sun	1	1	1	0	1	1	1	1	NC	77.78	High
Mchawa et al	1	1	1	1	1	1	1	1	1	100	High

Tunta et al	1	1	1	1	1	1	1	1	1	100	High
Wicaksana et al	1	1	1	1	1	1	1	1	1	100	High

Table 5. Risk of bias for included cohort studies in the systematic review.

**Author Reference**	**Were the two groups similar and recruited from the same population?**	**Were the exposures measured similarly to assign people to both exposed and unexposed groups?**	**Was the exposure measured in a valid and reliable way?**	**Were confounding factors identified?**	**Were strategies to deal with confounding factors stated?**	**Were the groups/ participants free of the outcome at the start of the study (or at the moment of exposure)?**	**Were the outcomes measured in a valid and reliable way?**	**Was the follow up time reported and sufficient to be long enough for outcomes to occur?**	**Was follow up complete, and if not, were the reasons to loss to follow up described and explored?**	**Were strategies to address incomplete follow-up utilized?**	**Total (%)**	**Quality level (High >50%, low <50%)**
Baker et al	1	1	1	1	0	0	1	1	1	0	70	High

Hechter et al												
Rosenthal et al	1	1	1	1	NC	1	1	1	1	NA	80	High
Strathdee et al	1	1	1	1	1	1	1	1	1	1	100	High
Javanbakht et al	1	1	1	1	1	1	1	1	1	NC	90	High
