# Supplementary material for: A systematic review and meta-analysis of the global prevalence and determinants of COVID-19 vaccine acceptance and uptake in people living with HIV
Source: Nat Hum Behav. 2023 Oct 30;8(1):100–14. doi: 10.1038/s41562-023-01733-3 (PMC10810755; doi:10.1038/s41562-023-01733-3)
Supplement: Supplementary file 2 — Reporting Summary [file 41562_2023_1733_MOESM2_ESM.pdf]

nature portfolio

Corresponding author(s): Sahab Kabir Sulaiman

Last updated by author(s): Sep 17, 2023



# Reporting Summary

Nature Portfolio wishes to improve the reproducibility of the work that we publish. This form provides structure for consistency and transparency in reporting. For further information on Nature Portfolio policies, see our Editorial Policies and the Editorial Policy Checklist.

## Statistics

For all statistical analyses, confirm that the following items are present in the figure legend, table legend, main text, or Methods section.

| n/a | Confirmed | |
|---|---|---|
| ☐ | ☒ | The exact sample size (*n*) for each experimental group/condition, given as a discrete number and unit of measurement |
| ☐ | ☒ | A statement on whether measurements were taken from distinct samples or whether the same sample was measured repeatedly |
| ☐ | ☒ | The statistical test(s) used AND whether they are one- or two-sided<br>*Only common tests should be described solely by name; describe more complex techniques in the Methods section.* |
| ☐ | ☒ | A description of all covariates tested |
| ☐ | ☒ | A description of any assumptions or corrections, such as tests of normality and adjustment for multiple comparisons |
| ☐ | ☒ | A full description of the statistical parameters including central tendency (e.g. means) or other basic estimates (e.g. regression coefficient) AND variation (e.g. standard deviation) or associated estimates of uncertainty (e.g. confidence intervals) |
| ☐ | ☒ | For null hypothesis testing, the test statistic (e.g. *F*, *t*, *r*) with confidence intervals, effect sizes, degrees of freedom and *P* value noted<br>*Give P values as exact values whenever suitable.* |
| ☒ | ☐ | For Bayesian analysis, information on the choice of priors and Markov chain Monte Carlo settings |
| ☒ | ☐ | For hierarchical and complex designs, identification of the appropriate level for tests and full reporting of outcomes |
| ☐ | ☒ | Estimates of effect sizes (e.g. Cohen's *d*, Pearson's *r*), indicating how they were calculated |

*Our web collection on statistics for biologists contains articles on many of the points above.*

## Software and code

Policy information about availability of computer code

| | |
|---|---|
| Data collection | Zotero software (version 6.0.15). The codes are available in the Supporting Information file and and the Open Source Framework page: https://osf.io/536qx/. |
| Data analysis | Stata Version 15IC (StataCorp, College Station, Texas USA), RevMan-5 Software (Version 5.4.1, The Nordic Cochrane Centre, Copenhagen) and Microsoft Excel and PowerPoint were used for data analysis and presentation. The direct outputs from these softwares are available in the Supporting Information file and and the Open Source Framework page: https://osf.io/536qx/. |

For manuscripts utilizing custom algorithms or software that are central to the research but not yet described in published literature, software must be made available to editors and reviewers. We strongly encourage code deposition in a community repository (e.g. GitHub). See the Nature Portfolio guidelines for submitting code & software for further information.

## Data

Policy information about availability of data

All manuscripts must include a data availability statement. This statement should provide the following information, where applicable:

- Accession codes, unique identifiers, or web links for publicly available datasets
- A description of any restrictions on data availability
- For clinical datasets or third party data, please ensure that the statement adheres to our policy

All data data supporting the findings of work presented here can be accessed via the Open Science Framewrok link, https://osf.io/536qx/.

## Human research participants

Policy information about studies involving human research participants and Sex and Gender in Research.

| | |
|---|---|
| Reporting on sex and gender | Carefully, we reported the findings based on gender as appropriate based on what was extracted from the included studies. |
| Population characteristics | See above |
| Recruitment | This was a systematic review and meta-analysis of available literature on the research subject at hand. |
| Ethics oversight | The study was pre-registered in the International Prospective Register for Systematic Review and Meta-analysis (PROSPERO), and the study has adhere to the Preferred Reporting Items For Systematic Review and Meta-analysis statement. |

Note that full information on the approval of the study protocol must also be provided in the manuscript.

# Field-specific reporting

Please select the one below that is the best fit for your research. If you are not sure, read the appropriate sections before making your selection.

☐ Life sciences ☒ Behavioural & social sciences ☐ Ecological, evolutionary & environmental sciences

For a reference copy of the document with all sections, see nature.com/documents/nr-reporting-summary-flat.pdf

# Behavioural & social sciences study design

All studies must disclose on these points even when the disclosure is negative.

| | |
|---|---|
| Study description | This was a systematic review and meta-analyses of quantitative studies reported any of the outcomes of interest. |
| Research sample | Global population of adult people living with HIV as reported in original studies done online/onsite in any place in the world. |
| Sampling strategy | No limitation regarding sampling strategy in our inclusion criteria so long as the study fulfilled all the inclusion criiteria |
| Data collection | Two researches (SKS and MSM) used the Joanna Briggs Institute data extraction form to extract relevant data from a;; included studies. |
| Timing | All studies published until 25th August 2023 were eligible for inclusion. |
| Data exclusions | 154 We only included original full-text articles, preprints, and abstracts evaluating any of our outcomes of interest (prevalence rates or factors associated with COVID-19 vaccine acceptability in PLHIV) that were reported in the English language |
| Non-participation | Not applicable. |
| Randomization | Not applicable |

# Reporting for specific materials, systems and methods

We require information from authors about some types of materials, experimental systems and methods used in many studies. Here, indicate whether each material, system or method listed is relevant to your study. If you are not sure if a list item applies to your research, read the appropriate section before selecting a response.

## Materials & experimental systems

| n/a | Involved in the study | |
|---|---|---|
| ☒ | ☐ | Antibodies |
| ☒ | ☐ | Eukaryotic cell lines |
| ☒ | ☐ | Palaeontology and archaeology |
| ☒ | ☐ | Animals and other organisms |
| ☒ | ☐ | Clinical data |
| ☒ | ☐ | Dual use research of concern |

## Methods

| n/a | Involved in the study | |
|---|---|---|
| ☒ | ☐ | ChIP-seq |
| ☒ | ☐ | Flow cytometry |
| ☒ | ☐ | MRI-based neuroimaging |
